# Digital resilience interventions for healthcare workers: a systematic review

**DOI:** 10.3389/fpsyt.2025.1519670

**Published:** 2025-09-10

**Authors:** Malin Larsson, Dominic M. Ho, Matthias Kirschner, Erich Seifritz, Andrei Manoliu

**Affiliations:** ^1^ Department of Psychiatry, Psychotherapy and Psychosomatics, Psychiatric University Hospital, University of Zurich, Zurich, Switzerland; ^2^ Department of Psychiatry, University Hospitals Geneva, Geneva, Switzerland; ^3^ Faculty of Medicine, University of Geneva, Geneva, Switzerland

**Keywords:** resilience, mental health, digital intervention, healthcare workers, healthcare students

## Abstract

**Introduction:**

Burnout among healthcare professionals is rising, exacerbated by increased workloads and the Covid-19 pandemic. Affected individuals face stress, depression, and anxiety, adversely impacting both personal well-being and patient care. Resilience has emerged as a key focus for targeted interventions, with online delivery gaining relevance due to the digital transformation and the need for flexibility in busy healthcare schedules.

**Methods:**

A systematic review was conducted by searching PubMed, Embase, and Web of Science for eligible studies from April 2014 to April 2024, using search terms related to resilience, online/blended interventions, and healthcare professionals. A total of 7,619 records were identified and screened by two independent reviewers (ML, AM). Final inclusion was based on predefined criteria for online or blended interventions aimed at enhancing resilience in healthcare professionals. The Effective Public Health Practice Project (EPHPP) assessed risk of bias. PRISMA guidelines were followed.

**Results:**

Fifty-five studies were selected, employing various interventions such as psychoeducation, meditation, mindfulness, and elements of cognitive-behavioral therapy (CBT) and acceptance and commitment therapy (ACT). Interventions were delivered online through websites, apps, audio files, etc. or in blended formats complementing in-person sessions. Most studies reported significant improvements in resilience, alongside reductions in stress, burnout, depression, and anxiety. However, only three studies in the online group involving mindfulness or CBT interventions received a strong global rating in the risk of bias assessment by fulfilling the methodological quality criteria. Among these, mindfulness, compared to a waitlist control or a psychoeducational brochure, significantly improved resilience and reduced burnout, while the CBT intervention, compared to bibliotherapy, led to a significant reduction in stress. Compared to the other studies, these three stood out due to minimal selection bias, low attrition rates, a robust study design, and at least partial blinding.

**Discussion:**

This review indicates that digital interventions may enhance resilience and associated factors in healthcare personnel. However, caution is advised due to the heterogeneity of interventions and varied measurement methods. Only three studies met methodological quality criteria, limiting the reliability of other findings. Future research should standardize resilience concepts and adhere to methodological criteria to ensure valid conclusions.

**Systematic review registration:**

https://www.crd.york.ac.uk/PROSPERO/view/CRD42024542758 PROSPERO, identifier CRD42024542758.

## 1 Introduction

Burnout, defined by emotional exhaustion (EE), depersonalization (DP), and reduced sense of accomplishment (PA), is a well-described and increasing issue in the healthcare sector worldwide. Various studies indicate burnout prevalence of 50% and higher in healthcare settings (1–4), a significantly higher rate compared to the general population (4–6).

Factors such as increasing workloads, extended working hours, demanding situations, personnel shortages, escalating bureaucracy, and the added impact of the COVID-19 pandemic, contribute to these challenging circumstances. Symptoms of overload and burnout are increasingly being reported, manifesting as depression, anxiety, fractured relationships, a rise in substance abuse and even an increased likelihood of subsequent suicidal ideation (7–9).

Burnout also has physical consequences, including heart disease, chronic pain, gastrointestinal issues, and even mortality (2) and further leads to absenteeism, resignations, turnover in personnel and a generally decreased job satisfaction (10).

However, it is not only the healthcare workers who are affected. Studies show that these issues also impact patient well-being and safety, leading to lower quality of care, reduced patient satisfaction, and higher rates of medical errors (2, 11).

Organizationally, declining productivity and increased employee absenteeism exacerbate the shortage of skilled professionals, further straining the remaining workforce. This is compounded by elevated turnover rates and reduced productivity, leading to financial consequences for healthcare institutions.

These impacts highlight the importance of mental health for healthcare employees. Consequently, there has been a rapid increase in interest in interventions aimed at preserving and promoting mental health. As therapy is often resource-intensive and costly, there is a growing recognition of the value of preventive measures. Accordingly, various studies have explored multiple interventions to enhance mental well-being (12).

A concept that has gained prominence in this context is resilience. Currently, there is no consensus on a precise definition, making objective measurement challenging (**Supplementary Figure 1** in **Supplementary Material**). In brief, resilience can be described as the ability to maintain psychological and physical health despite exposure to stressors, to rebound, recover, and grow when faced with adversity, and is influenced by genetic, epigenetic, developmental, neurochemical, and psychosocial factors (13, 14).

However, within organizational contexts, resilience is conceptualized at multiple levels, including individual, team, and organizational dimensions. Individual-focused models emphasize personal traits and coping mechanisms that enable employees to adapt to stress and adversity. These approaches highlight the importance of psychological resources such as self-efficacy and emotional regulation but may overlook the influence of organizational structures and culture. In contrast, organizational resilience frameworks consider systemic factors, such as leadership, communication, and adaptive capacity, recognizing that resilience emerges from the interaction between individuals and their work environment. While these models provide a broader understanding, they can be challenging to operationalize and measure. Integrating insights from both perspectives is essential for designing effective interventions that address the complex, multilevel nature of resilience in healthcare settings.

Several studies demonstrate the impact of resilience on mental health, showcasing its role in stress management and the prevention of burnout (3, 6, 15–19). As a result, numerous interventions have been developed to enhance resilience and psychological well-being. These include psychotherapeutic techniques, such as mindfulness-based therapy, emotional supportive coping, cognitive-behavioral therapy (CBT), acceptance and commitment therapy (ACT), attention and interpretation therapy, problem-solving therapy and coping strategies (15, 20, 21). Most interventions were delivered face-to-face in workshops and group-meetings. For instance, Kunzler et al. analyzed 44 randomized controlled trials on resilience-enhancing interventions, finding a positive effect on resilience and related outcomes. However, there was low certainty evidence for improvements, further limited by intervention heterogeneity (15). Another systematic review by Angelopoulou et al. included eleven studies on mindfulness techniques and emotional-supportive coping, showing small but significant positive effects on the resilience of physicians (21).

Although these interventions reported as effective in promoting mental well-being, scheduling in-person training within the already tight schedules of healthcare professionals can be challenging and may intensify stress. Additionally, online training is generally more cost-effective than in-person sessions (22–25). It also provides better accessibility, allowing healthcare workers to participate at their convenience, thus reducing barriers to access and participate. Therefore, offering an online intervention that can be conducted at preferred times is considered meaningful, efficient, and appreciated.

Several studies have examined interventions delivered through an online format. For example, Ladino et al. (20) investigated the effect of internet-based psychosocial interventions on professional burnout in a systematic review comprising four articles. In this review, findings could not demonstrate a significant post-intervention difference compared to the control group. Two studies underwent a meta-analysis, which failed to provide evidence of the interventions’ effectiveness in reducing burnout.

Another systematic review by Henshall et al. (26) explored the effectiveness of web-based interventions on the resilience of healthcare professionals, including eight studies indicating post-intervention enhancements in resilience. All eight studies demonstrated an improvement in resilience levels and associated symptoms following the web-based interventions.

Furthermore, a systematic review by Lopez-Del-Hoyo (27) investigated the effectiveness of eHealth interventions on stress reduction and improvement in the overall mental health of healthcare workers. In this review, 22 studies with different program types were compared, with 13 studies showing significant enhancements in stress, depression, anxiety, burnout, resilience, and mindfulness.

The aim of this study was to compile existing research on online resilience interventions in the healthcare sector and assess their effectiveness. Although there is already a wide variety of resilience interventions, unified approaches are lacking. By comparing different interventions and delivery formats, this review contributes to progress in this area.

By including the most current studies in this rapidly evolving field, this systematic review complements existing literature, further contributing to the preservation and promotion of resilience and overall mental health among healthcare professionals. Covering 55 studies that incorporate diverse psychological approaches and interventions across various healthcare professions, this review summarizes their impacts on resilience, stress, burnout, depression, anxiety, and well-being. It also compares purely digital delivery formats with blended versions to advance the implementation of these interventions.

## 2 Methods

This systematic review was conducted according to the Preferred Reporting Items for Systematic Review and Meta-Analyses (PRISMA) 2020 Checklist.

The systematic review protocol has been registered in the International Prospective Register of Systematic Reviews (PROSPERO) under registration number CRD42024542758.

### 2.1 Eligibility criteria

To be included, the studies should meet several criteria.

Regarding the population, all studies are included in which the intervention group (IG) consisted of individuals working in the healthcare sector, comprising physicians, nurses, paramedics, physiotherapists, and other unspecified healthcare professionals, along with students of healthcare sciences.

All studies incorporating an intervention, regardless of the presence of a control group (CG), whether randomized or non-randomized are included. The requirement is that both baseline and post-intervention data are collected.

The studies must be written in English or German.

Another criterion is that the studies involve interventions aiming at maintaining or promoting resilience or related factors. The specific content of the intervention is not predetermined.

All studies offering resilience interventions digitally or in a blended format incorporating both online and in-person aspects are taken into consideration.

An additional inclusion criterion concerns the measurements. Included are studies comprising quantitative measurements of resilience or related outcomes such as stress, burnout, depression, anxiety, or well-being.

The eligibility criteria are summarized below according to the PICOS scheme (**Table 1**).

**Table 1:** PICOS.

PICOS	Definition
Population	Healthcare personnel
Intervention	Digital/Blended Resilience/related Interventions
Comparison	Baseline, Control Groups, or between Intervention Groups
Outcome	Quantitative Measures of Resilience, Stress, Burnout, Depression/Anxiety, Well-Being
Study design	Interventional studies with a pre- post design, non-randomized and randomized controlled trials

All studies that do not meet the above criteria are excluded. This encompasses studies with a target population that is either not further specified or not employed in the healthcare sector; all reviews, study protocols, or similar works lacking an intervention design; and studies published in languages other than English or German. Additionally, interventions that are unrelated to resilience or related symptoms, interventions exclusively offering in-person or remote training, and studies with only qualitative outcome assessments and lacking a quantitative measurement method are also excluded.

### 2.2 Search strategy and data sources

For the systematic literature search, three databases were explored: PubMed, Embase, and Web of Science. The search strategy was based on three key terms: health care, resilience, and online or blended interventions. All articles published until the end of April 2024 were considered. In total, the search yielded 8271 search results.

### 2.3 Screening and study selection

All articles found from the search were extracted to Endnote and duplicates were deleted. The remaining studies were classified by relevance using an algorithm and sorted accordingly in an Excel spreadsheet. The AI (artificial intelligence) algorithm is based on the AS Review program. It is a language model that categorizes and prioritizes studies in the dataset under investigation based on their likely relevance using titles, sentences, and words. Hand-selected studies meeting the inclusion criteria serve as the reference (28).

Based on title and abstract, the majority of irrelevant studies were excluded in a first step. In a second step, the remaining studies were screened using the full text and ultimately included if the inclusion criteria were met. The detailed protocol can be found in the appendix (screening protocol).

More studies that met the inclusion criteria were identified from the reference lists of relevant studies and were added to the study pool.

This analysis was performed by two independent authors (ML, AM) and subsequently discussed until a final list of included studies was generated on Excel by joint consensus.

### 2.4 Data extraction and analysis

During the data extraction process, included studies were divided into two groups. The first group consisted of studies with purely digital, asynchronous interventions. The second group comprised studies that offered interventions in blended formats, combining both digital and in-person components.

From all included studies, the key information was extracted and presented in an Excel spreadsheet. This included the publication details (title, authors, year of publication, journal, location), population (number of participants, profession, age, proportion of female participants, attrition), intervention (content, delivery, duration of the intervention, frequency), outcome (what are the outcomes, how and when are they measured), study design and control group, results (resilience, stress, burnout, depression, anxiety, well-being), and correlations if investigated.

Given the significant heterogeneity between the two groups (online *vs*. blended), a subgroup analysis was conducted for better comparability, focusing on study characteristics, sample size and population characteristics, and intervention characteristics. A descriptive analysis was then performed to summarize the effectiveness and significate of the measured outcomes.

### 2.5 Risk of bias assessment

To assess the risk of bias, The Effect Public Health Practice Project (EPHPP) has been utilized. This involved considering selection bias, the study design, confounders, the blinding, the data collection method, and withdrawals and dropouts.

After capturing all the aforementioned factors, a global rating was created to illustrate the risk of bias.

### 2.6 Ethics

Ethics approval was not required for this study, as no primary data were utilized, and neither humans nor animals were directly involved. 

## 3 Results

### 3.1 Search outcomes

**Figure 1:**
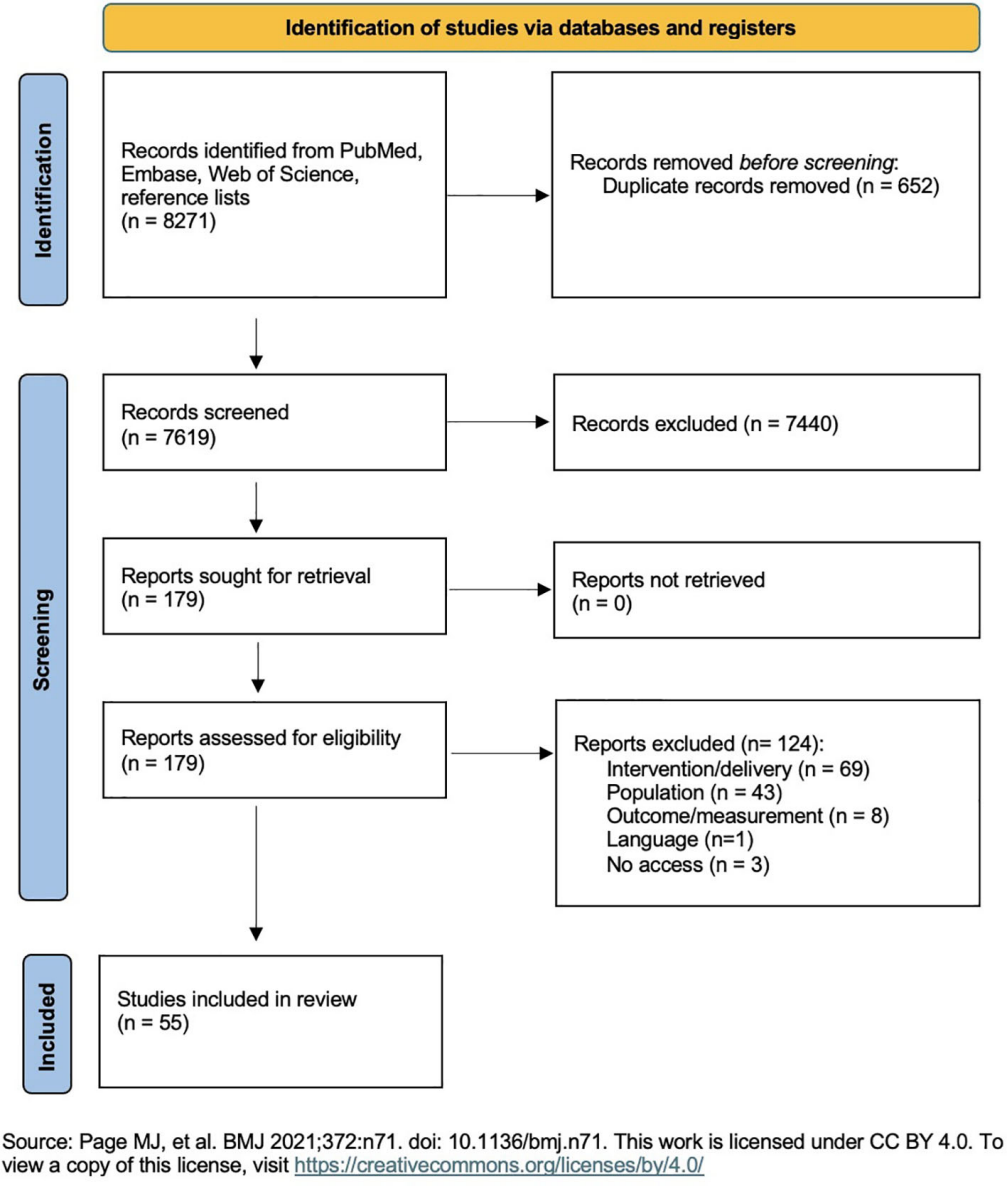
PRISMA 2020 flow chart.

See **Figure 1** for flow chart.

### 3.2 Online group

In total, 39 studies were assigned to the online intervention group (29–67).

#### 3.2.1 Study characteristics

The included studies, published between 2015 and 2024, comprised

14 randomized controlled trials (29, 41, 43–45, 48, 50, 51, 55, 59, 63–65, 67), 4 controlled trials (30, 35, 38, 42), and 20 with a pre-posttest design (31–34, 36, 37, 39, 46, 47, 49, 52–54, 56–58, 60–62, 66). One study focused solely on correlations post-intervention (40) (**Table 2**).

**Table 2:** Study characteristics of the online group.

Title	Year, publication	location	Study design	Control group
Long-term beneficial effects of an online mind-body training program on stress and psychological outcomes in female healthcare providers: A non-randomized controlled study.	2020 Wolters Kluwer Health	Seoul, Korea	Pre- and postintervention design, controlled trial	Yes
Acute Effects of Online Mind-Body Skills Training on Resilience, Mindfulness, and Empathy	2015 Journal of Evidence-Based Complementary & Alternative Medicine	Ohio, USA	Pre-posttest	No
Building Personal Resilience following an Online Resilience Training Program for BScN Students.	2022 Western Journal of Nursing Research	Western Canada	Pre- posttest	No
Randomized controlled trial of the “WISER” intervention to reduce healthcare worker burnout.	2021 Journal of Perinatology	Massachusetts, North Carolina, Tennessee, Texas, New Mexico, California	RCT (3-armed parallel design)	Yes (Waitlist control
Resilience Training for Work-Related Stress Among Health Care Workers: Results of a Randomized Clinical Trial Comparing In-Person and Smartphone-Delivered Interventions.	2018 Journal of Occupational and Environmental Medicine	Mayo Clinic, Arizona	RCT (3-armed parallel design)	Yes
Promoting resilience in healthcare workers during the COVID-19 pandemic with a brief online intervention.	2022 Journal of Psychiatric Research	Boston, Massachusetts	Pre-posttest, pragmatic, non-randomized	No
Brief Online Focused Attention Meditation Training: Immediate Impact.	2017 Journal of Evidence-Based Complementary & Alternative Medicine	Ohio, USA	Pre-posttest	No
What Is the Impact of Online Training in Mind-Body Skills?	2015 Journal of Evidence-Based Complementary & Alternative Medicine	Ohio, USA	Pre-posttest	Yes
Effectiveness of a bite-sized web-based intervention to improve healthcare worker wellbeing: A randomized clinical trial of WISER.	2022 Journal Frontiers in Public Health, Public mental health section	USA	RCT	Yes (waitlist control)
Online Training in Mind-Body Therapies: Different Doses, Long-term Outcomes	2017 Journal of Evidence-Based Complementary & Alternative Medicine	Ohio	Correlation of doses and responses	No
Impact of App-Delivered Mindfulness Meditation on Functional Connectivity, Mental Health, and Sleep Disturbances Among Physician Assistant Students: Randomized, Wait-list Controlled Pilot Study	2021 Journal of Medical Internet Research	Atlanta, USA	RCT	Yes (waitlist control)
Smartphone-based home workout program for shift-work nurses working during the COVID-19 pandemic.	2022 Nursing & Health Sciences, WILEY	Korea	Pre-posttest	Yes (non-equivalent control group)
Improving Healthcare Worker Resilience and Well-Being During COVID-19 Using a Self-Directed E-Learning Intervention.	2021 Frontiers in Psychology	South Africa	Pre- posttest	No
Effectiveness of self-help plus (SH+) in reducing anxiety and post-traumatic symptomatology among care home workers during the COVID-19 pandemic: a randomized controlled trial.	2021 Royal Society Open Science	Northern Italy	RCT (Prospective, parallel-group study	Yes
A Smartphone App to Reduce Burnout in the Emergency Department: A Pilot Randomized Controlled Trial.	2023 SAGE journals, Workplace Health & Safety	Calgary, Canada	RCT	Yes (waitlist control)
Evaluation of a Web-Based Holistic Stress Reduction Pilot Program Among Nurse-Midwives	2018 Journal of Holistic Nursing	Maryland, Virginia, USA	Pre-posttest	No
Help in hand after traumatic events: a randomized controlled trial in health care professionals on the efficacy, usability, and user satisfaction of a self-help app to reduce trauma-related symptoms	2020 European Journal of Psychotraumatology	Amsterdam, The Netherlands	RCT	Yes (no access to app)
The Nurse Empowerment Program for Nurses in Direct Care Positions	2022 The Journal of Nursing Administration	USA (& Canada)	Pre-posttest, pragmatic, non-randomized	No
Can stoic training develop medical student empathy and resilience? A mixed-methods study.	2022 BMC Medical Education	York, UK	Pre-posttest	No
Three good things: Promote work-life balance, reduce burnout, enhance reflection among newly licensed RNs	2022 Nursing Forum, an independent voice for nursing, Wiley	USA	Pre- posttest	No
Reduction of Burnout in Mental Health Care Providers Using the Provider Resilience Mobile Application.	2017 Community Mental Health Journal	USA	Pre- posttest	No
Well-being in Residency: Impact of an Online Physician Well-being Course on Resiliency and Burnout in Incoming Residents.	2021 Family Medicine	Arizona, USA	Pre- posttest	No
Decreasing Burnout and Improving Work Environment: The Impact of “Firgun” on a Pediatric Hematopoietic Cell Transplant Team.	2023 Journal of Clinical Oncology	Tennessee, USA	Pre- posttest	No
The Effects of an Online Mind-Body Training Program on Stress, Coping Strategies, Emotional Intelligence, Resilience and Psychological State	2016 PLoS One	Seoul National University Hospital, Republic of Korea	Non-randomized controlled trial	Yes
Exploring the effects of an online asynchronous mindfulness meditation intervention with nursing students on stress, mood, and cognition: a descriptive study	2016 Nurse Education Today	Pittsburgh, Pennsylvania, USA	Descriptive, exploratory study	No
Building personal resilience in paramedic students	2017 Journal of Community safety & well-being	British Columbia, Canada	RCT	yes
Building personal resilience in primary care paramedic students, and subsequent skill decay	2020 Australasian Journal of Paramedicine	British Columbia, Canada	Pre- posttest	No
Forty-five good things: a prospective pilot study of the Three Good Things well-being intervention in the USA for healthcare worker emotional exhaustion, depression, work–life balance and happiness	2019 BMJ Open	North Carolina, USA	Pre-Posttest	No
The Feasibility and Effectiveness of Online Guided Imagery Training for Health Professionals	2017 Journal of Evidence-Based Complementary & Alternative Medicine	Ohio, USA	Pre- posttest	No
Effectiveness of an online positive psychology intervention among Tunisian healthcare students on mental health and study engagement during the Covid-19 pandemic	2021 The International Association of Applied Psychology, Applied Psychology: Health & Wellbeing	Tunisia	2 armed RCT	Yes (waiting list)
Leadership Link: Evaluation of an Online Leadership Curriculum for Certified Midwives and Certified Nurse-Midwives	2023 Journal of Midwifery & Women’s Health	USA	cohort design (pre-post design)	No
Brief tele-mindfulness-based intervention: A multicenter randomized controlled trial.	2023 Journal of Family and Community Medicine	Egypt	RCT	Yes (different intervention groups)
A Mindfulness-Based Intervention for Acute Care Nursing Staff: A Pilot Study	2024 Journal of Holistic Nursing	USA	Pre-posttest	No
Impact of an online training tool on individual and organizational resilience and mindfulness among radiological personnel in Norway	2023 BMC	Norway	Pre-posttest	No
Feasibility, acceptability and preliminary efficacy of a mental health self-management app in clinicians working during the COVID-19 pandemic: A pilot randomised controlled trial	2023 Psychiatry Research	South Africa	RCT	Yes (waitlist control)
mHealth Gratitude Exercise Mindfulness App for Resiliency Among Neonatal Intensive Care Unit Staff: Three-Arm Pretest-Posttest Interventional Study.	2024 Journal of Medical Internet Research Nursing	United States	Pre-posttest interventional study	No
Efficacy of the my health too online cognitive behavioral therapy program for healthcare workers during the COVID-19 pandemic: A randomized controlled trial.	2024 Internet Interventions, Elsevier	France (Strasbourg, Colmar, Mulhouse, Nancy, Besançon, Dijon)	RCT	Yes (experimental vs. Active control group (bibliography)
Improving Resiliency in US Air Force Healthcare Personnel: A Randomized Preventive Trial	2024 Military Medicine	USA	Randomized, preventive trial	Yes (different intervention groups)
Guided self-help mindfulness-based intervention for increasing psychological resilience and reducing job burnout in psychiatric nurses: A randomized controlled trial.	2023 International Journal of Nursing Practice	Daqing, China	RCT	Yes

#### 3.2.2 Sample size and population characteristics

Across all studies, nearly 10’000 participants provided baseline data, with around 6600 of them receiving interventions. Post-intervention surveys were completed by approximately 3800 individuals, and around 2200 provided follow-up data. Most participants were women, primarily doctors, nurses, and students in these fields. Other professions included midwives, paramedics, social workers, psychologists, nutritionists, dentists, pharmacists, and physiotherapists (**Table 3**).

**Table 3:** Population characteristics of the online group.

Title	n	Profession	% Female
Long-term beneficial effects of an online mind-body training program on stress and psychological outcomes in female healthcare providers: A non-randomized controlled study.	56 MBT: 25 Control group: 31	Healthcare providers in Seoul National University Hospital	100%
Acute Effects of Online Mind-Body Skills Training on Resilience, Mindfulness, and Empathy	513	a) trainee (students, residents, fellows) vs. Practicing professional b) categorized as dietitians/nutritionists, nurses, physicians, social workers, health researchers, and others (psychologists, dentists, lab technicians, physical & occupational therapists, and others)	x
Building Personal Resilience following an Online Resilience Training Program for BScN Students.	70 resilience analysis: 32 depression, anxiety analysis: 36	Nursing students (in Bachelor of Science in Nursing curriculum)	Not reported
Randomized controlled trial of the “WISER” intervention to reduce healthcare worker burnout.	415 Cohort 1: 182 Cohort 2: 233	HCWs, nurses (62%), physicians, physician assistant, nurse practitioner	83%
Resilience Training for Work-Related Stress Among Health Care Workers: Results of a Randomized Clinical Trial Comparing In-Person and Smartphone-Delivered Interventions.	60 MBRT intervention: 22 Smartphone intervention: 23 Control group: 15	Employees at the Mayo Clinic in Arizona	86.70%
Promoting resilience in healthcare workers during the COVID-19 pandemic with a brief online intervention.	baseline sample: 554 longitudinal sample: 148	Physicians (13.9%), Nurses (26.9%), administrators, pharmacists, researchers, therapists, technicians, medical assistants	87%
Brief Online Focused Attention Meditation Training: Immediate Impact.	379	nurse (31%) medical assistants, advanced practice nurses, physicians (21%), other (48%: social worker, psychologist, etc.)	85%
What Is the Impact of Online Training in Mind-Body Skills?	103 (60 participated in MBS-training, 43 did not)	dietetics (n = 26), medicine (77), nursing (29), social work (52), others	MBS: 78% No MBS: 77%
Effectiveness of a bite-sized web-based intervention to improve healthcare worker wellbeing: A randomized clinical trial of WISER.	546 C1: 272 C2: 274	HCWs, nurses (62%), physicians, physician assistance, nurse practitioner	C1: 78.3% C2: 74.1%
Online Training in Mind-Body Therapies: Different Doses, Long-term Outcomes	149	nurses (38%), physicians (21%), others (social workers, psychologists, dietitians, researchers, etc.)	80%
Impact of App-Delivered Mindfulness Meditation on Functional Connectivity, Mental Health, and Sleep Disturbances Among Physician Assistant Students: Randomized, Wait-list Controlled Pilot Study	14	Physician Assistant students	IG: 86% CG: 71%
Smartphone-based home workout program for shift-work nurses working during the COVID-19 pandemic.	49	Shift-work nurses	100%
Improving Healthcare Worker Resilience and Well-Being During COVID-19 Using a Self-Directed E-Learning Intervention.	750	55.7% Primary care	77.6%
Effectiveness of self-help plus (SH+) in reducing anxiety and post-traumatic symptomatology among care home workers during the COVID-19 pandemic: a randomized controlled trial.	238 IG: 119 CG: 119	Workers of residential nursing & care homes Nursing & Care Homes = NCH): 75.63% HCW	88.24%
A Smartphone App to Reduce Burnout in the Emergency Department: A Pilot Randomized Controlled Trial.	20	Multidisciplinary health care professionals working in the pediatric emergency department (physician, nurses, respiratory therapists, social workers)	81.6%
Evaluation of a Web-Based Holistic Stress Reduction Pilot Program Among Nurse-Midwives	10	Midwives	100%
Help in hand after traumatic events: a randomized controlled trial in health care professionals on the efficacy, usability, and user satisfaction of a self-help app to reduce trauma-related symptoms	259	Healthcare Professionals (nurses, physicians, paramedics, ambulance drivers)	52.9%
The Nurse Empowerment Program for Nurses in Direct Care Positions	69 (pretest, posttest, follow-up) 170 (pretest, posttest)	Nurses	89%
Can stoic training develop medical student empathy and resilience? A mixed-methods study.	24	3rd-year medical students	62.5%
Three good things: Promote work-life balance, reduce burnout, enhance reflection among newly licensed RNs	19 participated for the entire 14d 49 engaged in any study activities	Newly licensed nurses	Not reported
Reduction of Burnout in Mental Health Care Providers Using the Provider Resilience Mobile Application.	30	Outpatient mental health providers psychologists (43%), social workers (30%), psychiatric nurses (13%), psychiatrists (7%) etc.	Not reported
Well-being in Residency: Impact of an Online Physician Well-being Course on Resiliency and Burnout in Incoming Residents.	53	Incoming postgraduate year-1 residents (PGY-1) in multiple residencies	45.3%
Decreasing Burnout and Improving Work Environment: The Impact of *Firgun* on a Pediatric Hematopoietic Cell Transplant Team.	25	Pediatric oncology physicians, nurses, allied health professionals	85.7%
The Effects of an Online Mind-Body Training Program on Stress, Coping Strategies, Emotional Intelligence, Resilience and Psychological State	87 IG: 42 CG: 45	HCW mostly nurses (or in jobs associated with caring for, or supporting, patients or members of the hospital)	100%
Exploring the effects of an online asynchronous mindfulness meditation intervention with nursing students on stress, mood, and cognition: a descriptive study	26	Nursing students	Not reported
Building personal resilience in paramedic students	138 IV: 81 CG: 57	Paramedic students	36.2%
Building personal resilience in primary care paramedic students, and subsequent skill decay	34	Paramedic students	Responders: 64.7% Non-responders: 47.2%
Forty-five good things: a prospective pilot study of the Three Good Things well-being intervention in the USA for healthcare worker emotional exhaustion, depression, work–life balance and happiness	121	HCW (19.8% nurses)	79.3%
The Feasibility and Effectiveness of Online Guided Imagery Training for Health Professionals	273	Nurses (34%), physicians (20%), social workers (14%), others	84%
Effectiveness of an online positive psychology intervention among Tunisian healthcare students on mental health and study engagement during the Covid-19 pandemic	324	Healthcare students (podiatry, emergency care, operating instrumentation, pediatric care, research master)	94%
Leadership Link: Evaluation of an Online Leadership Curriculum for Certified Midwives and Certified Nurse-Midwives	186	Midwives	98.9%
Brief tele-mindfulness-based intervention: A multicenter randomized controlled trial.	125 MBI: 64 PMR: 61	Healthcare providers (medicine/Surgery, nursing, respiratory therapy)	Medicine/surgery: 45.5% Nursing: 90.7% Respiratory therapy: 80%
A Mindfulness-Based Intervention for Acute Care Nursing Staff: A Pilot Study	31	Cardiovascular acute care nursing staff nurses (87.1%), nursing care technicians (6.5%), and advanced practice providers (6.5%)	83.9%
Impact of an online training tool on individual and organizational resilience and mindfulness among radiological personnel in Norway	68	Radiological personnel	Not reported
Feasibility, acceptability and preliminary efficacy of a mental health self-management app in clinicians working during the COVID-19 pandemic: A pilot randomized controlled trial	34 IG: 16 CG: 18	Clinicians working in government healthcare facilities	58.8%
mHealth Gratitude Exercise Mindfulness App for Resiliency Among Neonatal Intensive Care Unit Staff: Three-Arm Pretest-Posttest Interventional Study.	65	Neonatal Intensive Care Unit Staff	100%
Efficacy of the my health too online cognitive behavioral therapy program for healthcare workers during the COVID-19 pandemic: A randomized controlled trial.	147 experimental condition: 70 active control condition: 77	Healthcare workers (nurses, nursing students, doctors, physiotherapists, midwives, psychologists)	Experimental group: 84.3% Active control group: 84.4%
Improving Resiliency in US Air Force Healthcare Personnel: A Randomized Preventive Trial	49	Air Force Health personnel	66%
Guided self-help mindfulness-based intervention for increasing psychological resilience and reducing job burnout in psychiatric nurses: A randomized controlled trial.	99 IG: 52 CG: 48	Psychiatric nurses	IG: 88.5% CG: 87.2%

#### 3.2.3 Intervention characteristics

The interventions varied significantly in terms of content, format, duration, frequency, and intensity, reflecting the broad and complex nature of resilience. Generally, they aimed to enhance resilience and address symptoms like stress, burnout, depression, anxiety, and improve overall well-being. Participants were introduced to resilience concepts through educational materials, taught coping strategies, and engaged in practice-based learning to strengthen mental health.

Many studies emphasized mindfulness and mind-body skills using meditation, breathing exercises, relaxation, guided imagery, autogenic training, journaling, and yoga. Common themes included gratitude, kindness, social support, relationships, sleep, productivity, positivity, compassion, hardiness, and empathy.

The interventions, delivered online, utilized various resources like educational materials, quizzes, videos, audio files, podcasts, and self-paced workshops.

Fourteen interventions were entirely self-paced (29, 33, 36, 48, 49, 52, 53, 55–57, 61, 62, 64, 66), allowing individuals to decide the intensity of their practice. Fifteen interventions were pre-designed modules with a specified length that needed to be completed (31, 34, 37–39, 41–43, 45, 46, 50, 51, 58, 63, 65), and ten studies recommended a specific duration or frequency of training over a defined period (30, 32, 35, 40, 44, 47, 54, 59, 60, 67) (**Table 4**).

**Table 4:** Intervention characteristics of the online group.

Title	Content	Delivery	Time period, duration
Long-term beneficial effects of an online mind-body training program on stress and psychological outcomes in female healthcare providers: A non-randomized controlled study.	Mind-body training content and skills	Online (videos)	10min per session 1x per day, 5x per week for 8 weeks
Acute Effects of Online Mind-Body Skills Training on Resilience, Mindfulness, and Empathy	Mind-body skills training; Curriculum: Mind-Body Skills Training for Resilience, Effectiveness, and Mindfulness	Online (videos)	12x 1h, with no deadlines
Building Personal Resilience following an Online Resilience Training Program for BScN Students.	ORR = Online Resilience Resource (self-guided online training program which includes readings, videos, exercises, self-assessment tools)	Web-based/online	6-8h intervention, spread over 3 months
Randomized controlled trial of the “WISER” intervention to reduce healthcare worker burnout.	6 guided well-being modules	Electronically	10-20min per module Cohort 1: 10 days/month for 6 months Cohort 2: 28 days in 1 month
Resilience Training for Work-Related Stress Among Health Care Workers: Results of a Randomized Clinical Trial Comparing In-Person and Smartphone-Delivered Interventions.	Mindfulness-based resilience training. Smartphone app: sleep, happiness and positivity, energy and focus, productivity; mindfulness and feeling less stressed.	In person vs. Smartphone app	MBRT: 120min each session, 1x per week for 6 weeks.
Promoting resilience in healthcare workers during the COVID-19 pandemic with a brief online intervention.	Online resilience-enhancement course focused on mindfulness, mentalization and self-compassion.	Online (videos, available via an online platform HealthStreamTM)	3x 12-19min
Brief Online Focused Attention Meditation Training: Immediate Impact.	Online focused attention meditation training	Online	3x 1h with no deadlines
What Is the Impact of Online Training in Mind-Body Skills?	Mind-Body Skills training (focused attention meditation, mindfulness meditation, positive affect meditation, guided imagery/hypnosis)	Online	12 weeks with one 1h module weekly
Effectiveness of a bite-sized web-based intervention to improve healthcare worker wellbeing: A randomized clinical trial of WISER.	WISER intervention (Web-based Implementation for the Science of Enhancing Resilience): a positive psychology program	Web-based	10 days, 10-20min per module
Online Training in Mind-Body Therapies: Different Doses, Long-term Outcomes	Mind-Body Skills Training for Resilience, Effectiveness, and Mindfulness	Online course	HDS: 14h, MBST: 12h for ca. 8 weeks
Impact of App-Delivered Mindfulness Meditation on Functional Connectivity, Mental Health, and Sleep Disturbances Among Physician Assistant Students: Randomized, Wait-list Controlled Pilot Study	Mobile app-delivered mindfulness meditation	App	1-year subscriptions to the app, program: 8 weeks. 12min per day. Modules: 4.20 - 13.22min each
Smartphone-based home workout program for shift-work nurses working during the COVID-19 pandemic.	Smartphone-based home workouts	Smartphone app	3-5x per week for 18 weeks
Improving Healthcare Worker Resilience and Well-Being During COVID-19 Using a Self-Directed E-Learning Intervention.	Psychological well-being, self-care (Physical, Mind, Relationships, Emotions, Work), mindfulness and meditation	E-learning (narrated presentation slides, additional resources: journal articles, websites)	5 modules needed to be completed within a month, self-paced
Effectiveness of self-help plus (SH+) in reducing anxiety and post-traumatic symptomatology among care home workers during the COVID-19 pandemic: a randomized controlled trial.	SH+: an individual self-help audio-visual tool: Doing what matters in times of stress. 5 core components: grounding, unhooking, acting on your values, being kind, making room.	Online (self-help audio-visual tool, pre-recorded audio course, illustrated self-help book)	5 weeks intervention, self-paced
A Smartphone App to Reduce Burnout in the Emergency Department: A Pilot Randomized Controlled Trial.	Resilience curriculum on 6 domains: mindfulness, self-expertise, mental fitness, mental health, hardiness, energy management	Smartphone application (includes text, images, videos, audio media)	3 months with 8-10h of total instructional content
Evaluation of a Web-Based Holistic Stress Reduction Pilot Program Among Nurse-Midwives	Web-based holistic stress reduction program using yoga, mindfulness-based stress reduction techniques and meditation	Web-based (interactive webpages, photographic demonstration, audio files, videos, written instructions)	4x per week for 4 weeks, 5-30min per module
Help in hand after traumatic events: a randomized controlled trial in health care professionals on the efficacy, usability, and user satisfaction of a self-help app to reduce trauma-related symptoms	SUPPORT COACH app: psychoeducation about trauma, PTSS, professional care; support section to facilitate contact with one’s personal network and professional care; self-test section: PTSD checklist for DSM-5; calendar section; manage symptoms section: exercises to self-manage PTSS	Online via app	1 month access to app, self-paced
The Nurse Empowerment Program for Nurses in Direct Care Positions	The Nurse Empowerment Program: LinkedIn learning, nursing continuing professional development, critical conversations, asynchronous discussion platform	Online courses (via Sigma’s learning management system LMS)	program accessible for 3.5 months, 34 - 84min per LinkedIn course; 1.6 - 5.4h per Sigma course
Can stoic training develop medical student empathy and resilience? A mixed-methods study.	Stoicism informed online training package: guided reflective diary	Online	15-20min daily for 12 days
Three good things: Promote work-life balance, reduce burnout, enhance reflection among newly licensed RNs	3 good things - regarding joys, work, self-efficacy, self-care, relationships	Web-based (daily text reminder via SMS: 3GT prompts, E-consent, daily recording of 3GTs)	14 days, daily, self-paced
Reduction of Burnout in Mental Health Care Providers Using the Provider Resilience Mobile Application.	The Provider Resilience (PR) mobile app includes 2 assessment tools to help increase self-awareness of current levels of burnout: Professional Quality of Life Scale and the Burnout Visual Analog Scale. The app screen provides an overall graphic of the user’s current resilience rating, customizable resilience builders and killers (encourages to be aware of factors that in-/decrease their resilience). Tools to help enhance resilience and reduce burnout, f.e. humorous cartoons, physical exercises, inspirational cards with motivational quotes), videos of consumers indication how their treatment impacted their lives, and video information on compassion fatigue.	Smartphone app	1 month, self-paced usage per week
Well-being in Residency: Impact of an Online Physician Well-being Course on Resiliency and Burnout in Incoming Residents.	Physician Well-being Course (PWC): well-being (resilience, sleep, nutrition, mindfulness, exercise); resilience activities (gratitude, meditation, finding meaning)	Online	10 min activity daily, 4.5h online course for 14 days
Decreasing Burnout and Improving Work Environment: The Impact of **“**Firgun**”** on a Pediatric Hematopoietic Cell Transplant Team.	Meaningful recognition: creating a culture of recurring altruistic complements related to job performance.	Web-based	8 weeks, weekly self-paced training
The Effects of an Online Mind-Body Training Program on Stress, Coping Strategies, Emotional Intelligence, Resilience and Psychological State	MBT on resilience, coping strategies, anger, stress, emotional intelligence, positive/negative affect. Movement-based meditation.	Online	10min, 5x per week for 8weeks
Exploring the effects of an online asynchronous mindfulness meditation intervention with nursing students on stress, mood, and cognition: a descriptive study	Online mindfulness intervention: Mindfulness-based stress reduction (MBSR) model components	Online	24 weeks - intervention: 8 weeks, mind- 1x per week. Self-paced
Building personal resilience in paramedic students	Online resiliency training program	self-paced online, blended with a practicum	2 weeks, 6-8h self-paced online couse
Building personal resilience in primary care paramedic students, and subsequent skill decay	ORR: Online Resilience Resource	Online	3/6/9 months (depending on follow-up timepoint), self-paced training, approx. 6-8h
Forty-five good things: a prospective pilot study of the Three Good Things well-being intervention in the USA for healthcare worker emotional exhaustion, depression, work–life balance and happiness	3 Good Things intervention	Online	2 weeks intervention, self-paced, follow-up: 12 months
The Feasibility and Effectiveness of Online Guided Imagery Training for Health Professionals	Brief online guided imagery training	Online	3x 1h
Effectiveness of an online positive psychology intervention among Tunisian healthcare students on mental health and study engagement during the Covid-19 pandemic	The CARE program: Coherence, Attention, Relationship, Engagement	Internet-based	8 sessions with 45min each, 1x weekly for 8 weeks
Leadership Link: Evaluation of an Online Leadership Curriculum for Certified Midwives and Certified Nurse-Midwives	The Leadership Link Program is an educational platform that aims to build leadership capacity and consists of 2 components: (1) 10 LinkedIn Learning leadership courses; (2) associated ACNM (American College of Nurse-Midwives) videos. The program improves leadership knowledge and skills to work effectively. Leadership domains: Understanding the context, professional expertise, self-awareness and self-development, communication as a change agent, operationalization and execution, transforming the future of midwifery.	Online (video-based courses)	10 weeks, self-paced, course 1: 11h, course 2: 10x8min
Brief tele-mindfulness-based intervention: A multicenter randomized controlled trial.	Brief Mindfulness-based interventions (MBI) and progressive muscle relaxation (PMR). MBI: 7 mediation commands. PMR: tensions and relaxing specific muscle groups in sequence.	Website with audio recording sessions, MP3	MBI: 20min daily, PMR: 20min daily sessions for 2 weeks
A Mindfulness-Based Intervention for Acute Care Nursing Staff: A Pilot Study	Mindfulness-based: Headspace consisted of modules with different meditations, Insight Timer comprised guided meditation with topics like presence and grounding awareness.	Smartphone application (headspace/Insight Timer)	30days with 5min daily
Impact of an online training tool on individual and organizational resilience and mindfulness among radiological personnel in Norway	Mindfulness and resilience training with 3 resilience exercises three good things, upside of stress and self-compassion), 3 mindfulness exercises (breath as anchor, body-scan, and walking meditation. Background Information on mindfulness, stress and stress management was delivered.	Online tool (comprising the WordPress platform, videos, audio-guides and documents)	Self-paced training, baseline: July-October 2022, follow-up: November 2022 - February 2023
Feasibility, acceptability and preliminary efficacy of a mental health self-management app in clinicians working during the COVID-19 pandemic: A pilot randomised controlled trial	COVID Coach: self-management app comprising the following topics: Manage stress, Learn, Mood check, and find resources. These courses teach how to manage with mental wellbeing using relaxation and mindfulness exercises, deliver psychoeducational information and support by offering help links.	Mental health app with an audio guided format	1 month, self-paced
mHealth Gratitude Exercise Mindfulness App for Resiliency Among Neonatal Intensive Care Unit Staff: Three-Arm Pretest-Posttest Interventional Study.	mHealth intervention using a smartphone app: gratitude, exercise, and mindfulness smartphone app (GEM app). 3 evidence-based resilience interventions (a daily gratitude journal, regular exercise, or mindfulness meditation) were delivered, of which participants could choose one.	Smartphone app	3 weeks, daily self-paced training
Efficacy of the my health too online cognitive behavioral therapy program for healthcare workers during the COVID-19 pandemic: A randomized controlled trial.	Cognitive Behavioral therapy via the online MyHealthToo app. The app consisted of 7 sessions: (1) psychoeducation, (2) functional behavioral and cognitive coping strategies, (3) mindfulness, (4) acceptance, (5) promoting action toward values, (6) addressing barriers and motivation to use self-compassion as a psychological gift, (7) sleep problems and problem-solving strategies.	Online	7 online sessions, 20min each, for 8 weeks
Improving Resiliency in US Air Force Healthcare Personnel: A Randomized Preventive Trial	Stress Management and Resilience Training (SMART). Online Training with 4 modules	2h training session in-person/face-to-face or video teleconference training, or via a self-paced, computer-based training	online modules take less than 60min each, 1–2 weeks to complete each online session. for 4–8 weeks
Guided self-help mindfulness-based intervention for increasing psychological resilience and reducing job burnout in psychiatric nurses: A randomized controlled trial.	The self-help mindfulness intervention aimed to guide participants to focus on the present moment and to learn how to practice in a non-judgmental fashion during stressful experiences. The program included didactic instructions and practice with topics like Mindfulness, Concentration, and Awareness in Sports. It comprised principles of identifying habitual reaction patterns and cultivating non-judgmental awareness and acceptance of the present. The practice included body scan, mindful walking, breathing meditation, and transposition exercise.	didactic information: recordings (audio files), text material WeChat on mobile phones to send audio recordings and text materials	didactic instructions: 8x 20-30min, 5x per week for 8 weeks

#### 3.2.4 Outcomes

The following table summarizes the results of individual studies on resilience, stress, burnout, depression, anxiety, and well-being, including any investigated correlations (**Table 5**). In each study, a p-value < 0.05 was considered significant.

**Table 5:** Results of the online group.

Title	Outcomes
Long-term beneficial effects of an online mind-body training program on stress and psychological outcomes in female healthcare providers: A non-randomized controlled study.	Resilience: Significant time x group across time found for T0-T1 and T0-T2. Greater increase in total resilience score in the MBT group that sustained over 1 month time. However, significant results achieved only for 1 subscale (strength for overcoming stress). Stress: No time x group interaction effect in occupational stress. Significant main effect of time (stress increased in both groups). No significant main effect of group (no difference between groups at baseline or follow-up). Significant time x group interaction in total stress when comparing T0 vs. T1/T2 (MBT group with a greater decrease in stress response than the control group). Depression: MBT group with a significantly greater decrease in depression compared to the control group from T0 to T1 and T0 to T2 when analyzing time x group interaction.
Acute Effects of Online Mind-Body Skills Training on Resilience, Mindfulness, and Empathy	Resilience: Significant improvement for Mindfulness in daily life, no significant improvement for Introduction to Stress and Resilience: non-significant improvement. Stress: Significant reduction in stress for Introduction to Stress and Resilience and autogenic training.
Building Personal Resilience following an Online Resilience Training Program for BScN Students.	Resilience: Significant improvement in resilience from baseline to post-training, 1-month post-, 3-months post-intervention Depression, Anxiety: Non-significant decrease
Randomized controlled trial of the “WISER” intervention to reduce healthcare worker burnout.	Burnout (EE): Significant improvement at 1 and 6 months Depression: Significant improvement at 1 and 6 months
Resilience Training for Work-Related Stress Among Health Care Workers: Results of a Randomized Clinical Trial Comparing In-Person and Smartphone-Delivered Interventions.	Stress: Smartphone group: no significant decrease at 6 weeks and 3 months, MBRT group: significant decrease at 6 weeks and 3 months, control group: no significance Burnout: Smartphone group: No significant improvements at 6 weeks and 3 months, MBRT group: significant EE improvements at 6 weeks and 3 months, control group: no significance Depression: Smartphone group: No significant improvements, MBRT group: no significant improvements, control group: no significant improvements Anxiety: Smartphone group: No significant improvements, MBRT group: no significant improvements, control group: no significant improvements Well-Being: Smartphone group: Significant improvements at 3 months, not at 6 weeks, MBRT group: Significant improvements at 3 months, not at 6 weeks, control group: no significance Correlation: Significant between number of sessions attended and increases in well-being scores.
Promoting resilience in healthcare workers during the COVID-19 pandemic with a brief online intervention.	Resilience: No significant changes at 1month postintervention. Significant improvement at 2 months postintervention Emotional distress: Significant decrease in emotional distress (PHQ-4) at 1 month and 2 months post-intervention. Correlations: Greater levels of baseline resilience were correlated with lower levels of emotional distress. After completion of the intervention, improvements in resilience were associated with lower levels of emotional distress.
Brief Online Focused Attention Meditation Training: Immediate Impact.	Resilience: Significant improvement between pre- and post-intervention. Stress: Significant reduction in stress between pre- and post-intervention. Burnout: 60% met at least one criterion for burnout. No follow-up data.
What Is the Impact of Online Training in Mind-Body Skills?	Resilience: No significant differences in resilience score between groups. Stress: Significant decrease in perceived stress after completion of MBS training. Increase in stress in those who did not engage in MBS.
Effectiveness of a bite-sized web-based intervention to improve healthcare worker wellbeing: A randomized clinical trial of WISER.	Resilience: Significant improvements in emotional thriving and emotional recovery at all timepoints. Burnout (EE): Significant reductions at all timepoints. Depressive symptoms: Significant reductions at all timepoints
Online Training in Mind-Body Therapies: Different Doses, Long-term Outcomes	Correlations: Mind-Body Skill practice frequency was associated with resilience levels. Negative association between the frequency of mind-body practice and perceived stress. Perceived Stress was associated with work missed in the past 30 days and burnout. Correlation between the frequency of mind-body training and stress, burnout, resilience.
Impact of App-Delivered Mindfulness Meditation on Functional Connectivity, Mental Health, and Sleep Disturbances Among Physician Assistant Students: Randomized, Wait-list Controlled Pilot Study	Burnout: No significant improvement Depression, Anxiety: No significant improvements
Smartphone-based home workout program for shift-work nurses working during the COVID-19 pandemic.	Resilience: Significant group-by-time difference that was increased in the intervention group. No significance in control group. Non-significant differences in those who exercised 3–5 days a week and those who do less or stopped exercise.
Improving Healthcare Worker Resilience and Well-Being During COVID-19 Using a Self-Directed E-Learning Intervention.	Resilience: Significant increase between pre- and posttraining. Well-Being: Significant increase between pre- and posttraining. Correlations: Significant positive associations between changes in behavior, coping mechanisms for stress, resilience scores, and well-being, with increased well-being and coping positively affecting resilience scores
Effectiveness of self-help plus (SH+) in reducing anxiety and post-traumatic symptomatology among care home workers during the COVID-19 pandemic: a randomized controlled trial.	Resilience: No significant improvement at post-intervention/follow-up. Stress: No significant improvement at post-intervention/follow-up. Anxiety: No significant improvements at post-intervention/follow-up. Well-Being: No significant improvement at post-intervention/follow-up.
A Smartphone App to Reduce Burnout in the Emergency Department: A Pilot Randomized Controlled Trial.	Resilience: No significant improvement post-intervention. No changes in the control group. Burnout: Significant decrease in EE. No significant improvements in DP or PA. No significant reduction in the control group.
Evaluation of a Web-Based Holistic Stress Reduction Pilot Program Among Nurse-Midwives	Stress: 25% reduction in the perceived stress score
Help in hand after traumatic events: a randomized controlled trial in health care professionals on the efficacy, usability, and user satisfaction of a self-help app to reduce trauma-related symptoms	Resilience: Significant difference between group with the intervention group scoring a greater increase than the control group from T1 to T3. No significant differences between groups from T1 to T2. Significant increase from T1 to T2 and from T1 to T3 in the intervention group Correlations: No relationship between number of performed exercises and any of the outcome measures or changes in T2-T1 and T3-T1 delta scores.
The Nurse Empowerment Program for Nurses in Direct Care Positions	Resilience: Significant increase between pre- and posttest. No significant differences between the follow-up and the pre-/posttest scores.
Can stoic training develop medical student empathy and resilience? A mixed-methods study.	Resilience: Significant increase. Correlations: Significant between resilience and empathy, no significant correlation between resilience and stoic ideation change.
Three good things: Promote work-life balance, reduce burnout, enhance reflection among newly licensed RNs	Resilience: Significant improvement in emotional thriving between baseline and T1. Improvements made at T1 in emotional thriving decreased by T2. Significant improvement in emotional recovery at T2, but not at T1. BUT: When only participants that participated for 14 days were included, evidence was weaker. Burnout: Significant improvement between baseline and T1. No significant improvement at T2 sustained. Correlations: Positive correlation between number of days on which exercises carried out and improvements in burnout scores.
Reduction of Burnout in Mental Health Care Providers Using the Provider Resilience Mobile Application.	Resilience: No significant improvements Burnout: Significant decrease
Well-being in Residency: Impact of an Online Physician Well-being Course on Resiliency and Burnout in Incoming Residents.	Resilience: Significant improvements Burnout: Significant improvement in burnout (EE, DP). Decline in the PA scale.
Decreasing Burnout and Improving Work Environment: The Impact of *Firgun* on a Pediatric Hematopoietic Cell Transplant Team.	Stress: No significant changes Burnout: No significant improvements Well-being: No significant improvements Correlations: No correlations found for any paired differences in total scale scores from pre- to posttest.
The Effects of an Online Mind-Body Training Program on Stress, Coping Strategies, Emotional Intelligence, Resilience and Psychological State	Resilience: Significant time x group interaction following the intervention: Stress: Significant time x group interaction following the intervention. Depression: Significant time x group interaction in depression. Correlation: Negative correlation between stress and emotional intelligence in the intervention group.
Exploring the effects of an online asynchronous mindfulness meditation intervention with nursing students on stress, mood, and cognition: a descriptive study	Stress: Significant reduction Depression: No significant reduction Anxiety: Significant reduction when training was performed weekly to daily. Correlations: between stress reduction and frequency of meditation practice.
Building personal resilience in paramedic students	Resilience: Significant improvement in total resilience and each of the subscores except meaningfulness.
Building personal resilience in primary care paramedic students, and subsequent skill decay	Resilience: No significant improvement from baseline to 3-months follow-up. Significant decrease at 6 or 9 months compared to baseline assessment. GROUPS: Completion of all three phases (baseline, intervention, follow-up): Statistically significant reduction in resilience scores from baseline to follow-up. 3-months: Non-significant improvement from baseline to follow-up at 3 months. 6-months: Significant decrease from baseline to follow-up. 9-months: Significant decrease from baseline to follow-up.
Forty-five good things: a prospective pilot study of the Three Good Things well-being intervention in the USA for healthcare worker emotional exhaustion, depression, work–life balance and happiness	Burnout (EE): Significant reduction from baseline to follow-up at 1 month, 6 months, 12 months. Depression: Significant improvement at 1,6, 12 months Correlation: No significant correlations were found between change scores and number of completed 3GT days. (p > 0.05).
The Feasibility and Effectiveness of Online Guided Imagery Training for Health Professionals	Stress: Significant reduction following completion of the autogenic training module. Anxiety: Significant reduction following completion of the autogenic training module.
Effectiveness of an online positive psychology intervention among Tunisian healthcare students on mental health and study engagement during the Covid-19 pandemic	Stress: Significant change from baseline to posttest and follow-up. No changes in the control group. Depression, Anxiety: Significant change from baseline to posttest and follow-up. No changes in the control group. Well-being: Significant change from baseline to posttest and follow-up. No changes in the control group.
Leadership Link: Evaluation of an Online Leadership Curriculum for Certified Midwives and Certified Nurse-Midwives	Resilience: Significant change from baseline to posttest and follow-up.
Brief tele-mindfulness-based intervention: A multicenter randomized controlled trial.	Resilience: No significant improvement. Anxiety: Significant reduction of state anxiety. No significant improvement in trait anxiety. Well-being: Significant improvement. The MBI group achieved significantly bigger improvements in well-being than the PMR group.
A Mindfulness-Based Intervention for Acute Care Nursing Staff: A Pilot Study	Resilience: No statistically significant chances across the three timepoints. Stress: Significant reductions from baseline to both midpoint and follow-up. Burnout: Significant reductions in personal burnout from baseline to midpoint and follow-up. No statistically significant reduction in work-related burnout.
Impact of an online training tool on individual and organizational resilience and mindfulness among radiological personnel in Norway	Resilience: Statistically significant lower resilience scores at follow-up compared to baseline in individual resilience.
Feasibility, acceptability and preliminary efficacy of a mental health self-management app in clinicians working during the COVID-19 pandemic: A pilot randomized controlled trial	Resilience: No significant improvement. Stress: No significant improvement. Burnout: No significant improvement. Anxiety: Significant reduction from pre to postintervention. Well-being: No significant improvement.
mHealth Gratitude Exercise Mindfulness App for Resiliency Among Neonatal Intensive Care Unit Staff: Three-Arm Pretest-Posttest Interventional Study.	Burnout: Significant reduction in the gratitude intervention group
Efficacy of the my health too online cognitive behavioral therapy program for healthcare workers during the COVID-19 pandemic: A randomized controlled trial.	Resilience: No significant changes Stress: Significant stress reduction post-therapy at 8, 12, 24 weeks Depression: No significant changes
Improving Resiliency in US Air Force Healthcare Personnel: A Randomized Preventive Trial	Resilience: Significant improvements at all timepoints (12w, 18w, 24w). No significant differences between groups (online vs. In-person) Stress: Significant reductions all timepoints (12w, 18w, 24w). No significant differences between groups (online vs. In-person). Anxiety: Significant reductions at all timepoints. No significant differences between groups (in-person vs. Online)
Guided self-help mindfulness-based intervention for increasing psychological resilience and reducing job burnout in psychiatric nurses: A randomized controlled trial.	Resilience: Significant main effect of intervention, time, and a significant interaction between intervention and time. No changes over time in the control group. Burnout: Significant main effect of intervention, time, and a significant interaction between intervention and time. No changes over time in the control group. Correlations: Significant correlation between resilience and job burnout: score reduction of CD-RISC was negatively correlated with the score reduction of MBI.

##### 3.2.4.1 Resilience

A total of 29 studies ( (29–40, 42, 44, 47, 48, 50, 54–57, 59–65, 67) examined resilience as an outcome. Of these, 19 studies (29–37, 39, 42, 47, 50, 54, 55, 61–63, 67) achieved significant results, while 10 (38, 44, 48, 56, 57, 59, 60, 64, 65) did not. One study focused solely on correlations and provided no numerical data (40). Three studies demonstrated a significant time x group interaction (30, 35, 42) following mind-body training or home workouts.

Post-intervention, 16 studies (29–33, 35–37, 39, 42, 47, 50, 55, 61, 63, 67) showed significant improvements in resilience, while five did not (44, 48, 60, 64, 65). Long-term follow-ups revealed significant results in eight studies (33, 42, 50, 54, 55, 61–63), but three did not show sustained improvements (31, 48, 65). One study found significant results for a resilience enhancement course only after two months, with no significance at one month (34). Another study utilizing the Online Resilience Resource (ORR) intervention achieved non-significant improvements after three months, with a decrease in resilience after six and nine months (56). The Nurse Empowerment Program achieved significance post-intervention, but results were not sustained at follow-up (31).

In summary, interventions such as mind-body and skills training, physical home workouts, 3 good things, mindfulness and self-compassion exercises, focused attention meditation and programs such as WISER, SMART, the Leadership Link program, the Support Coach app, the ORR course and the PWC course achieved significant results. Conversely, interventions like the headversity app, the Self-Help + (SH+) program or the COVID coach app did not show significant improvements.

Measurement tools included the Korean version of the Connor-Davidson Resilience Scale (CD-RISC) (42), Smith’s (6-item) Brief Resilience Scale (37–40), Resilience Scale for Adults (RSA) (54, 56), Connor-Davidson Resilience Scale (CD-RISC) (30, 31, 35, 36, 44, 47, 48, 57, 59, 61–65, 67), Brief Resilience Scale (BRS) (32, 34, 60), Resilience Evaluation Scale (55), 8-item Resilience Scales (emotional thriving, emotional recovery) (33), Dispositional Resilience Scale (47), and the Resilience scale (RS) (29).

##### 3.2.4.2 Stress

17 studies (35, 37–43, 46, 48, 52, 53, 58, 60, 63–65) examined stress, with 12 reporting significant results (35, 37–39, 41, 42, 46, 52, 58, 60, 63, 65), and four not achieving significance (43, 48, 53, 64). One study only examined correlations and did not provide any numerical data (40). Two studies showed a significant group x time interaction (35, 42) with mind-body training.

Post-intervention, 11 studies reported significant stress reductions (37–39, 41, 42, 46, 52, 58, 60, 63, 65), 4 did not (43, 48, 53, 64). Among seven studies that examined follow-up results (41–43, 48, 52, 63, 65), five achieved significant results (41, 42, 52, 63, 65), two did not (43, 48).

Overall, mind-body and skills training, positive psychology, focused attention and mindfulness meditation, guided imagery training, mindfulness via the Headspace app, CBT, as well as the care program, Stress Management and Resilience Training (SMART) program, and the holistic stress reduction program, all brought significant improvements in stress. Conversely, SH+ and Firgun, the COVID coach app, as well as MBSR (Mindfulness-based stress reduction) and ACT intervention, did not achieve significant stress reduction. Long-term success in stress reduction was observed with the Care program, CBT, SMART, mind-body training, and mindfulness meditation.

Measurement tools included the Korean Occupational Stress Scale (KOSS) (35, 42), Stress Response Inventory (Stress, depression) (42), Perceived Stress scale (PSS) (37, 39, 46, 48, 52, 53, 58, 60, 63–65), and the Cohen’s 10-item Perceived Stress Scale (38, 40).

##### 3.2.4.3 Burnout

16 studies (33, 39, 40, 43–45, 47, 49–51, 53, 57, 60, 64, 66, 67) recorded the outcome burnout. 11 studies achieved significant relevance (33, 44, 45, 47, 49, 50, 53, 57, 60, 66, 67), 3 did not (43, 51, 64). One study measured burnout numerically only once, preventing comparisons (39), and another focused solely on correlations without numerical data (40).

Post-intervention, nine studies showed statistically relevant results (33, 44, 47, 49, 50, 53, 57, 60, 66, 67), two did not (51, 64). Two studies (headversity app and Firgun intervention) achieved significant improvements only in EE, with DP and PA remaining non-significant (44, 53). In follow-up surveys, three studies showed significant relevance (45, 49, 50), two did not (33, 43). One study implementing the 3 good things intervention showed relevance post-interventional, but the effect could not be maintained until follow-up (33).

In conclusion, the headversity app, the WISER intervention, the three good things (3GT) intervention, mindfulness and gratitude exercises, the physician well-being course, the Firgun program, the Headspace app and the Provider Resilience Mobile Application all achieved significant improvements in burnout. In contrast, a MBSR and ACT intervention, the COVID coach app, as well as a mindfulness meditation intervention, did not result in significant burnout reduction.

Measurement tools included the Maslach Burnout Inventory (EE, DP, PA) (34, 39, 44, 45, 47, 49, 50, 53, 67), MBI-Human Services Survey (MBI-HSS) (43), EE (33), 7-item Mayo Clinic Physician Well-Being Index (PWBI) (40), School Burnout Inventory (51), Professional Quality of Life Scale (ProQOL) (57, 66), Copenhagen Burnout Inventory (CBI) (60, 64).

##### 3.2.4.4 Depression/anxiety

17 studies (34, 35, 41–43, 45, 46, 48–52, 54, 59, 63–65) measured depression and/or anxiety, with 12 achieving statistical significance (34, 35, 41, 42, 45, 46, 49, 50, 52, 59, 63, 64) and 5 not (43, 48, 51, 54, 65). Two studies utilizing positive psychology or resilience interventions focusing on mindfulness, meditation, and self-compassion, showed significant reductions in depressive and anxious symptoms (34, 41). Five studies reported significant improvement in depressive symptoms (35, 42, 45, 49, 50), and 5 in anxiety symptoms (46, 52, 59, 63, 64). Two studies analyzing mind-body training found a significant time x group interaction in depression (35, 42).

Post-intervention, seven studies reached statistical significance (35, 42, 46, 50, 52, 63, 64), three did not (43, 48, 51, 65). One study achieved a significant improvement in state anxiety following a brief mindfulness intervention, whereas trait anxiety showed no significant change (59). In the follow-up surveys, eight studies achieved statistical relevance (34, 41, 42, 45, 49, 50, 52, 63), four did not (43, 48, 54, 65).

Overall, interventions such as positive psychology, the WISER intervention, the SMART program, the COVID coach app, mind-body training, guided imagery training, 3GT, mindfulness meditation, and a mindfulness, mentalization, and self-compassion program yielded significant reductions in symptoms of depression and anxiety. Specifically, the WISER intervention, mind-body training, and 3GT brought about improvements in depressive symptoms, while guided imagery training and mindfulness meditation showed an effect on anxiety. The SH+, ORR, CBT, and MBSR interventions did not have a significant effect on depression or anxiety.

Measurement tools included the Generalized Anxiety Disorder 7-items (GAD-7) (48, 54, 63), The Patient Health Questionnaire (PHQ) (34, 54, 65), Center for Epidemiological Studies Depression Scale 10-item version (CES-D10) (45, 49, 50, 64), Depression, Anxiety and Stress Scale (DASS-21) (41, 43, 51), The Hospital Anxiety and Depression scale (HADS) (52), Patient Reported Outcomes Measurement Information System – Anxiety Scale (46), Stress Response Inventory (depression) (35), and the State-Trait Anxiety Scale (59, 64).

##### 3.2.4.5 Well-being

7 studies (36, 41, 43, 48, 53, 59, 64) measured the outcome well-being, with four reporting significance (36, 41, 43, 59) and three not (48, 53, 64).

Post-interventional, 2 studies showed significant improvements (36, 59), 3 did not (48, 53, 64). 2 studies achieved significant relevance in the follow-up measurements (41, 43), one of which was relevant after 3 months, but the results were not yet significant at 6 weeks (43). Another study did not reach statistical significance at follow-up (48).

Interventions that demonstrated a significant positive influence on well-being included positive psychology, a MBSR and ACT intervention, and a mindfulness and meditation intervention. The SH+, the COVID coach app and Firgun interventions remained without a significant relevance.

Measurement tools included the WHO-5 Well-Being Index (WHO-5) (36, 43, 48, 53, 59, 64) and the Warwick-Edinburgh Mental Well-being Scale (WEMWBS) (41).

In summary, the WISER program could improve resilience and reduce burnout, the SMART program demonstrated significant stress reduction and improved resilience, and the Leadership Link program showed effectiveness in enhancing resilience. The Three Good Things (3GT) intervention led to improvements in both resilience and burnout, while mindfulness practices and self-compassion exercises were effective in enhancing resilience as well. The Headspace app significantly reduced stress and burnout, but the Headversity app, Self-Help + (SH+), and the COVID Coach did not show significant improvements in any outcomes. Mind-body training and guided imagery effectively improved resilience, as well as symptoms of depression and anxiety. The Firgun intervention failed to achieve significant benefits across the measured outcomes.

These findings are based on studies that predominantly employed quantitative tools to assess resilience, such as various versions of the Connor-Davidson Resilience Scale (CD-RISC), the Brief Resilience Scale (BRS), and the Resilience Scale for Adults (RSA). While these instruments differ somewhat in conceptual orientation, most assess resilience as a dynamic, modifiable capacity rather than a fixed trait. For instance, the CD-RISC and BRS conceptualize resilience as an individual’s current ability to adapt and recover from stress, which can be enhanced through targeted interventions. In contrast, measures such as the Dispositional Resilience Scale also incorporate more stable trait-like elements, capturing a person’s general predisposition to cope with adversity. Overall, the use of primarily state-oriented measurement tools aligns with the interventions’ goals of promoting improvements in psychological adaptability and recovery.

A detailed table with the results for each outcome is located in the appendix (**Supplementary Table 10**).

##### 3.2.4.6 Correlations

In 13 studies (31–36, 40, 43, 49, 52, 53, 55, 67), various correlations were analyzed. Significant relationships were found between outcomes such as the number of sessions attended, and improvements in well-being scores (43), stress (40, 52), burnout (33, 40) and resilience (40). Resilience was also linked emotional distress (34) and empathy (32). Positive associations were observed between changes in resilience and well-being, as well as coping mechanisms for stress and improvements in well-being and resilience (36). Additionally, a positive correlation was found between job demand and perceived stress, while stress negatively correlated with emotional intelligence (35). Lastly, resilience was linked to job burnout (67).

##### 3.2.4.7 Students

Seven (29, 32, 37, 41, 52, 54, 65) out of nine studies (29, 32, 37, 41, 51, 52, 54, 56, 65) examining the effects of interventions on students found that mind-body skills, psychoeducation, CBT, and positive psychology had a positive impact on resilience, burnout, stress, depression, anxiety, and well-being.

### 3.3 Blended group

A total of 16 studies were allocated to the blended group (68–83).

#### 3.3.1 Study characteristics

All included studies were published between 2016 and 2024, comprising 7 randomized controlled trials (68, 69, 77, 79–81, 83), one uncontrolled trial (78), and 8 studies with a pre-posttest interventional design (70–76, 82) (**Table 6**).

**Table 6:** Study characteristics of the blended group.

Title	Year, Publication	Location	Study design	Control group
Decreasing Stress and Burnout in Nurses: Efficacy of Blended Learning With Stress Management and Resilience Training Program.	2017 Wolters Kluwer Health, JONA (The Journal of Nursing Administration)	Rochester, Minnesota	1-group baseline to postintervention	No
Impact of a Blended Web-Based Mindfulness Programme for General Practitioners: a Pilot Study	2018 Springer Science + Business Media New York	Madrid, Spain	open, uncontrolled trial, pre- posttest (different groups depending on the number of sessions made)	No
Implementation of a Web-Based Resilience Enhancement Training for Nurses: Pilot Randomized Controlled Trial.	2023 Journal of Medical Internet Research	South of England	1:1 two-armed pilot randomized trial	Yes (waitlist control)
The impact of Stress Management and Resailience Training (SMART) on academic physicians during the implementation of a new Health Information System: An exploratory randomized controlled trial.	2022 PLOS ONE	Ottawa, Canada	RCT	yes
Mind-Body Skills Training for Resident Wellness: A Pilot Study of a Brief Mindfulness Intervention.	2018 Journal of Medical Education and Curricular Development	Ohio, USA	Pre- postintervention	No
Interventions to reduce burnout and improve resilience: Impact on a health system**’**s outcomes	2019 Clinical Obstetrics and Gynecology, Wolters Kluwer Health	Ohio, USA	Pre- posttest	No
Sustained resiliency building and burnout reduction for healthcare professionals via organizational sponsored mindfulness programming.	2022 Explore (Elsevier)	Ohio, USA	Pre-posttest	No
The Community Resiliency Model¬Æ to promote nurse well-being.	2019 Nursing Outlook, Elsevier	Atlanta, USA	RCT, parallel design	Yes (nutrition education control group)
Brief video-module administered mindfulness program for physicians: a pilot study	2016 Explore (Elsevier)	Tacoma, Washington USA	single-sample, pre-posttest	no
Training on mind-body skills: Feasibility and effects on physician mindfulness, compassion, and associated effects on stress, burnout, and clinical outcomes	2020 The Journal of Positive Psychology	Ohio, USA	Prospective cohort study	Yes (originally no control group but comparison of intervention group to those who didn’t complete one module)
Effects of the Brief Simha Kriya Breathing Practice for Health Care Workers During the COVID-19 Pandemic.	2024 Journal of Integrative and Complementary Medicine	USA	Prospective, single-arm trial	No
A comparative study of well-being, resilience, mindfulness, negative emotions, stress, and burnout among nurses after an online mind-body based intervention during the first COVID-19 pandemic crisis	2023 Frontiers in Psychology	Mexico	Uncontrolled trial	No
A compassion-based program to reduce psychological distress in medical students: A pilot randomized clinical trial	2023 PLOS ONE	Madrid, Spain	RCT	Yes (waitlist control)
Effectiveness of an online mental health strengthening module to build resilience and overcome stress for transitional aged medical students	2023 Frontiers in Digital Health	Indonesia	RCT	Yes
Feasibility and acceptability of a culturally adapted psychological first aid training intervention (Preparing Me) to support the mental health and well-being of front-line healthcare workers in China: a feasibility randomized controlled trial	2024 European Journal of Psychotraumatology	China	RCT	Yes
Efficacy of a Text-Based Mental Health Coaching App in Improving the Symptoms of Stress, Anxiety, and Depression: Randomized Controlled Trial	2023 Journal of Medical Internet Research	Malaysia	RCT	Yes

#### 3.3.2 Sample size and population characteristics

In total, over 2800 individuals provided baseline data, with nearly 2400 participating in the intervention and about 1500 submitting post-intervention or follow-up data.

Most participants were nurses and physicians, and two studies exclusively involved medical students (79, 81). The majority of participants across the studies were female (**Table 7**).

**Table 7:** Population characteristics of the blended group.

Title	n	Profession	% Female
Decreasing Stress and Burnout in Nurses: Efficacy of Blended Learning With Stress Management and Resilience Training Program.	50	Nurses	92%
Impact of a Blended Web-Based Mindfulness Programme for General Practitioners: a Pilot Study	290	General Practitioners	77.5%
Implementation of a Web-Based Resilience Enhancement Training for Nurses: Pilot Randomized Controlled Trial.	107 IG: 56 CG: 51)	Nurses	88.8%
The impact of Stress Management and Resilience Training (SMART) on academic physicians during the implementation of a new Health Information System: An exploratory randomized controlled trial.	32	Physicians (Department of Medicine)	IV: 35% CG: 45%
Mind-Body Skills Training for Resident Wellness: A Pilot Study of a Brief Mindfulness Intervention.	10	Pediatric resident physicians	70%
Interventions to reduce burnout and improve resilience: Impact on a health system’s outcomes	CRM: >7500 MIM: unknown Mind-body skills: unknown Gabbe: 70 flipped classroom: 66	Emergency medicine, internal medicine, surgeons; 49% physicians, 24% nurses	Not reported
Sustained resiliency building and burnout reduction for healthcare professionals via organizational sponsored mindfulness programming.	66	Healthcare professionals (28% physicians, 24% nurses, etc.)	83%
The Community Resiliency Model¬Æ to promote nurse well-being.	77 IG: 40 CG: 37	Nurses (emergency department, operating room, intensive care unit, specialty units, out-patient clinics, medical-surgical units)	95%
Brief video-module administered mindfulness program for physicians: a pilot study	19	Physicians (internal medicine, surgery, sleep medicine, obstetrics/gynecology, anesthesia)	39.1%
Training on mind-body skills: Feasibility and effects on physician mindfulness, compassion, and associated effects on stress, burnout, and clinical outcomes	50	28% ED Physicians 40% IM physicians 32% surgery physicians	48%
Effects of the Brief Simha Kriya Breathing Practice for Health Care Workers During the COVID-19 Pandemic.	100	Healthcare workers	88%
A comparative study of well-being, resilience, mindfulness, negative emotions, stress, and burnout among nurses after an online mind-body based intervention during the first COVID-19 pandemic crisis	643	Nurses	83%
A compassion-based program to reduce psychological distress in medical students: A pilot randomized clinical trial	40 IG: 18 CG: 22	Medical students	92.5%
Effectiveness of an online mental health strengthening module to build resilience and overcome stress for transitional aged medical students	105 IG: 52 CG: 53	Medical students	60.9%
Feasibility and acceptability of a culturally adapted psychological first aid training intervention (Preparing Me) to support the mental health and well-being of front-line healthcare workers in China: a feasibility randomized controlled trial	96 (48 in each group)	Healthcare workers (doctors, midwives, nurses, pharmacists)	IG: 89.7% CG: 90.2%
Efficacy of a Text-Based Mental Health Coaching App in Improving the Symptoms of Stress, Anxiety, and Depression: Randomized Controlled Trial	334 (167 in each group)	Trainee physicians, students, faculty members, and corporate staff at International Medical University, Malaysia.	64.2%

#### 3.3.3 Intervention characteristics

The included studies offered interventions focused on stress management, mindfulness and awareness, mind-body skills, meditation, and yoga as resilience training, delivered in a blended format. All studies featured an online, asynchronous component for independent completion, alongside workshops conducted in-person or remotely via platforms like Zoom, providing content explanations, addressing questions, facilitating practice exercises, and enabling group discussions. The online components varied in duration, intensity, and frequency; five studies supplemented in-person interventions with self-paced online content (68, 70, 71, 78, 79), while eight combined in-person training with structured online modules (69, 72–76, 81, 83). Two studies provided online training with remote support (80, 82), and one made online training entirely optional (77) (**Table 8**).

**Table 8:** Intervention characteristics of the blended group.

Title	Content	Delivery	Time period, duration
Decreasing Stress and Burnout in Nurses: Efficacy of Blended Learning With Stress Management and Resilience Training Program.	SMART: Stress Management and Resiliency Training	Web-based format, independent reading, facilitated discussion/combination; access to online content + a copy of the book (participants could choose whatever fitted best for them)	8 weeks, self-paced
Impact of a Blended Web-Based Mindfulness Programme for General Practitioners: a Pilot Study	Mindfulness intervention based on the standard program by Kabat-Zinn	blended: 1 face-to-face meeting, 8 online trainings	10h (4h in person, 6h online) over a 1month period, in-person meeting: 4h, online training: practices required 45min per session, 2x per week for 4 weeks = 8x in total recommended
Implementation of a Web-Based Resilience Enhancement Training for Nurses: Pilot Randomized Controlled Trial.	The REsOluTioN Program: Resilience Enhancement Online Training for Nurses	Web-based: blended (Teams/online) synchronous & asynchronous learning approach: web-based large-group facilitated sessions on the weekly modules; modules: preparatory learning on module topics prior to the group-sessions; small group mentoring sessions	August 2021 to May 2022, training: 4 weeks, Online modules: 4x30min; mentoring sessions: 30-60min, 2x/w. Web-based, large group sessions: 4x120min;
The impact of Stress Management and Resailience Training (SMART) on academic physicians during the implementation of a new Health Information System: An exploratory randomized controlled trial.	SMART: The Stress Management and Resilience Training program. Principles: gratitude, acceptance, compassion, higher meaning, forgiveness	blended: 1 mandatory in-person workshop, optional online program	24 weeks in total, 2h in-person workshop mandatory, optional online program for 24 weeks: 4-week TRAIN (4 modules, each 45min, 1x per week), 20 weeks SUSTAIN (20modules, 10-15min, 1x per week)
Mind-Body Skills Training for Resident Wellness: A Pilot Study of a Brief Mindfulness Intervention.	MBST: Mind-Body Skills Training for Resilience, Effectiveness, and Mindfulness	blended: in-person peer-led training supported by online modules	1 month, optional: 6 months. 4x 90min MBST sessions, 8 online modules, optional maintenance sessions once per month in-person/remote.
Interventions to reduce burnout and improve resilience: Impact on a health system**’**s outcomes	CRM: Crew Resource Management MIM: Mindfulness in Motion training, Gabbe health and wellness program, Mind-body skills	flipped classroom: independent learning & sharing with peers during interactive sessions; 4 online modules, 3 interactive discussion sessions	CRM: 4h, MIM: 1h/week for 8 weeks (8h in total), followed by 1h monthly booster sessions for 6months
Sustained resiliency building and burnout reduction for healthcare professionals via organizational sponsored mindfulness programming.	Mindfulness Based Intervention (MBI): Mindfulness in Motion (MIM)	Website, group sessions	1h group session weekly, online interventional part self-paced for 8 weeks
The Community Resiliency Model¬Æ to promote nurse well-being.	Community Resiliency Model training (CRM): sensory awareness techniques to improve emotional balance	blended: 3h class CRM (in person)	3h class, access to the CRM ichill app
Brief video-module administered mindfulness program for physicians: a pilot study	Mindfulness training, body scan, guided meditations	8 video-modules, that were continuously available 3 live sessions, teleconferences	3x 90min in-person trainings, weekly online video-module trainings (5-7min each), weekly teleconference coaching calls for 8 weeks
Training on mind-body skills: Feasibility and effects on physician mindfulness, compassion, and associated effects on stress, burnout, and clinical outcomes	Online Mind-Body Skills Training and interactive discussion sessions	4 online modules on MBST, 3 interactive discussion sessions length of modules: 5-20min each	7h: 4h online, 3h interactive discussion sessions
Effects of the Brief Simha Kriya Breathing Practice for Health Care Workers During the COVID-19 Pandemic.	A brief pranayama yoga practice: breathwork practice. Simha Kriya yoga: forced exhalation with tongue sticking out, 21x at a normal pace; forced exhalation with tongue rolled upward, 21x, at a normal pace; breath retention for 30-60s; followed by a 2min meditation. Weekly support sessions via zoom.	Online prerecorded video, weekly support sessions via zoom	5 min, 1-2x/day for 4 weeks
A comparative study of well-being, resilience, mindfulness, negative emotions, stress, and burnout among nurses after an online mind-body based intervention during the first COVID-19 pandemic crisis	Mind-body based intervention that comprises 36 mind-body based micro practices. Nurse leaders provided support by sharing the exercises 3x per week in a group setting. Videos and audio files that teach techniques including relaxation response, mindfulness-based stress reduction, single-focus meditation, self-regulation exercises, breathing practices, awareness practices, spiritually and reframing strategies based of existential positive psychology, and journaling, were offered.	Online using a web platform, Zoom	self-paced at home, weekly sessions via zoom for 12 weeks
A compassion-based program to reduce psychological distress in medical students: A pilot randomized clinical trial	Compassion Cultivation Training (CCT): meditation program that aims to cultivate compassion and empathy to reduce psychological distress and promote well-being. The program includes the following 6 steps: (1) learning to focus and settle the mind; (2) experiencing compassion and loving-kindness for a loved one; (3) experiencing compassion and loving-kindness for oneself; (4) experiencing compassion towards others, premised in common humanity and interconnectedness; (5) experiencing compassion towards all beings; and (6) “active compassion” practice.	Group format with discussions, practical exercises, pedagogical instructions, guided group meditations.	2h online sessions weekly, 30 min daily home practice for 8 weeks
Effectiveness of an online mental health strengthening module to build resilience and overcome stress for transitional aged medical students	The Transition and Adaptation towards Resiliency module consists of 4 submodules: (1) Changes in life to become more independent and adapting to a new environment; (2) stress and ways to overcome stress; (3) mental health problems and symptoms of mental disorders; (4) mental health help-seeking	Website, module was guided online by 5 facilitators, online discussion sessions, Zoom meetings	2 meetings à 90min, self-paced learning online for 4 weeks
Feasibility and acceptability of a culturally adapted psychological first aid training intervention (Preparing Me) to support the mental health and well-being of front-line healthcare workers in China: a feasibility randomized controlled trial	Preparing me is a psychological first aid training intervention. READ-Y PFA (psychological first aid) training program consisted of elements of trauma recovery principles, specific techniques and case scenarios. READ-Y PFA: Rapport, Evaluation, Aid, Disposition, Care for Yourself and others.	Face-to-face PFA training 4 online group PFA sessions	1d training: 5x 75min in-person group sessions, 4 online group supervision self-paced sessions
Efficacy of a Text-Based Mental Health Coaching App in Improving the Symptoms of Stress, Anxiety, and Depression: Randomized Controlled Trial	ThroughFullChat: One-on-one, asynchronous, text-based coaching and self-guided tools	Online app	3 months, self-paced

#### 3.3.4 Outcomes

The following table summarizes the outcomes for each study, with a significance level set at p<0.05 (**Table 9**).

**Table 9:** Results of the blended group.

Title	Outcomes
Decreasing Stress and Burnout in Nurses: Efficacy of Blended Learning With Stress Management and Resilience Training Program.	Resilience: Significant improvement at week 24, not significant at week 8 and 12. Stress: Significant reduction at week 24. Burnout: Personal Burnout: Significant reduction in personal and work-related burnout at week 8,12, 24. Significant decrease in client related burnout at week 12 and 24, but not at week 8. Anxiety: Significant decrease at week 8, 12,24.
Impact of a Blended Web-Based Mindfulness Programme for General Practitioners: a Pilot Study	Resilience: No significant improvements when practicing one or twice per week. Burnout: No significant decrease.
Implementation of a Web-Based Resilience Enhancement Training for Nurses: Pilot Randomized Controlled Trial.	Resilience: No significance. Well-being: No significance.
The impact of Stress Management and Resilience Training (SMART) on academic physicians during the implementation of a new Health Information System: An exploratory randomized controlled trial.	Resilience: No significant improvements at 3- and 6-months follow-up. Stress: No significant improvements at 3- and 6-months follow-up. Anxiety: No significant improvements at 3- and 6-months follow-up.
Mind-Body Skills Training for Resident Wellness: A Pilot Study of a Brief Mindfulness Intervention.	Resilience: Significant improvement between T1 and T2. No significant changes between T1 and T3. Stress: Significant improvement between T1 and T2. No significant changes between T1 and T3. Burnout: Significant improvement in PA between T1 and T2. No significant changes in PA between T1 and T3. No significant changes in EE and DP.
Interventions to reduce burnout and improve resilience: Impact on a health system’s outcomes	Stress: No significant improvements after completion of at least 1 intervention Burnout: Significant improvement in EE and DP from pre- to postintervention when at least 1 intervention is done. Well-being: Significant improvement from pre- to postintervention when at least 1 intervention is done
Sustained resiliency building and burnout reduction for healthcare professionals via organizational sponsored mindfulness programming.	Resilience: Significant improvement from baseline to posttest and follow-up. Stress: Significant improvement from baseline to posttest and follow-up. Burnout: Significant improvement from baseline to posttest and follow-up In those who initially met burnout critera.
The Community Resiliency Model¬Æ to promote nurse well-being.	Resilience: Significant improvement over time. Group-by-time not significant. Burnout: No significant improvement over time. Group-by-time effect is not significant. Well-being: Significant improvement over time. Group-by-time effect is not significant.
Brief video-module administered mindfulness program for physicians: a pilot study	Stress: Significant decrease between baseline and follow-up at 8 weeks. Burnout: Significant improvement in PA between baseline and follow-up at 8 weeks. No significant changes in EE and DP.
Training on mind-body skills: Feasibility and effects on physician mindfulness, compassion, and associated effects on stress, burnout, and clinical outcomes	Stress: No significant improvements Burnout: Significant mean difference in burnout (EE, DP) after participating. Well-being: Significant mean difference in participants who completed at least 1 hour of training. Correlations: Number of modules completed explained a significant proportion of variance in EE and PWBI. The amount of completed modules and interactive sessions did not predict DP scores.
Effects of the Brief Simha Kriya Breathing Practice for Health Care Workers During the COVID-19 Pandemic.	Resilience: Significant improvement at 4-week follow-up. Stress: No significant changes.
A comparative study of well-being, resilience, mindfulness, negative emotions, stress, and burnout among nurses after an online mind-body based intervention during the first COVID-19 pandemic crisis	Resilience: No significant improvements Stress: Significant reduction Burnout: No significant improvements Well-being: No significant improvements
A compassion-based program to reduce psychological distress in medical students: A pilot randomized clinical trial	Resilience: No significant difference after the intervention compared to the waitlist control. Stress: Significant reduction in stress after the intervention. No significant changes in the waitlist control. Burnout (EE): Significant reduction following the intervention and at follow-up. No significant changes in the waitlist control. Depression, Anxiety: Significant reductions in depression and anxiety following the intervention, significance remained in anxiety at follow-up. No significant changes in the waitlist control. Well-being: No significant difference after the intervention compared to the waitlist control.
Effectiveness of an online mental health strengthening module to build resilience and overcome stress for transitional aged medical students	Resilience: Significant changes over time. No significant differences compared to the control group after completion of the intervention. Stress: Significant changes over time. Significant difference after completion of the intervention compared to the control group. Depression, Anxiety: Significant changes over time. No significant differences compared to the control group after completion of the intervention. Correlation: Significant interaction between intervention and time in resilience, stress, depression, and anxiety
Feasibility and acceptability of a culturally adapted psychological first aid training intervention (Preparing Me) to support the mental health and well-being of front-line healthcare workers in China: a feasibility randomized controlled trial	Resilience: Significant improvements over time. Stress: Significant reduction over time. Burnout: Significant reduction over time. Depression, Anxiety: Significant reduction over time. Correlation: Significant correlation between depression and burnout.
Efficacy of a Text-Based Mental Health Coaching App in Improving the Symptoms of Stress, Anxiety, and Depression: Randomized Controlled Trial	Resilience: No significant changes the intervention. Stress: Significant reduction in the intervention group among corporate staff. Depression, Anxiety: Significant reduction in depression and anxiety in the intervention group and in females compared with males.

##### 3.3.4.1 Resilience

Seven studies (68–71, 73, 76, 77) measured resilience, with four studies achieving statistical relevance (68, 70, 71, 76), three did not (69, 73, 77).

Post-intervention, one study showed a significant improvement in resilience (76), whereas four did not (69, 71, 73, 77). At follow-up, two studies reached significance (70, 71), two did not (76, 77). One study failed to significantly improve resilience postintervention and after four weeks but achieved it at 16 weeks follow-up (71). Another study demonstrated significant results immediately after the intervention but lost effects at the six months follow-up (76).

In summary, significant increases in resilience were found in studies involving mindfulness and mind-body skills training, as well as programs like “Preparing Me”, the “Transition and Adaptation Towards Resiliency module”, the “Community Resilience Model”, and the “iChill app”. The SMART program showed significant improvement in one of two studies, while “Compassion Cultivating Training” and “ThoughfullChat” did not yield significant changes.

Measurement tools included the CD-RISC (68, 70, 71, 73, 77), Brief Resilience Scale (69), and the Smith’s Brief Resilience Scale (76).

##### 3.3.4.2 Stress

Seven studies examined the outcome of stress (70–72, 74–77), with four achieving significant results (70, 71, 75, 76), and three not (72, 74, 77). Five studies measured perceived stress post- intervention (71, 72, 74–76), of which two reported significant reductions (75, 76). At follow-up, two studies showed significant improvements (70, 71), whereas two did not (76, 77). One study examining effects of mind-body skills achieved significant stress reduction post-intervention but could not sustain it at follow-up (76).

Overall, interventions incorporating mindfulness and mind-body skills achieved significant stress reductions, as did the “Compassion Cultivating Training”, the “Transition and Adaptation towards Resiliency” module, and the “Preparing Me” program. The SMART intervention reached significance in one of two studies, with only a non-significant trend in the other. The “Crew Resource Management” program and a yoga intervention did not yield significant results.

The Perceived Stress Scale (70–72, 74–77) was used to measure changes in stress.

##### 3.3.4.3 Burnout

Eight studies assessed burnout symptoms (68, 70–76), with six reporting significant reductions (70–72, 74–76), and two did not (68, 73).

Immediately after the intervention, five studies achieved significant reductions in burnout (71, 72, 74–76), while two did not (68, 73). Among the four studies that measured over an extended period (68, 70, 71, 76), three studies realized statistically significant improvement at follow-up (70, 71, 76). Romcevich et al. measured burnout subthemes EE, DP, and PA (76) following a mind-body skills intervention. While no significant reduction could be achieved in EE immediately after the intervention or at the follow-up, a significant improvement in DP was observed between baseline and follow-up. A significant increase in PA was also achieved between baseline and post-intervention, but this improvement was not sustained until the follow-up. Pflugeisen et al. could achieve significant improvements in PA between baseline and postintervention survey using a mindfulness intervention. However, decreases in EE and DP between baseline and postintervention remained non-significant (75).

A significant reduction in burnout symptoms was achieved by the SMART intervention, mindfulness and mind-body skills trainings, as well as the “Compassion Cultivating Training” and the “Preparing Me” program. The “Community Resilience Training” did not result in a significant reduction in burnout symptoms.

Measurement tools included the Burnout Inventory (68, 71), Burnout Clinical Subtype Questionnaire (BSCQ-12) (73), and the Maslach Burnout Inventory (70, 72, 74–76).

##### 3.3.4.4 Depression, anxiety

Two studies evaluated symptoms of depression and anxiety (71, 77). One study significantly reduced anxiety symptoms at postintervention and at follow-ups (71), while the other did not (77).

A significant reduction in depression and anxiety was achieved through the “Compassion Cultivating Training”, the “Transition and Adaptation Towards Resiliency” module, the “Preparing Me” program, and “ThoughtfullChat”. The SMART intervention reached significant in one of two studies, with a non-significant trend in the other.

The Generalized Anxiety Scale (71, 77) was used for measurement.

##### 3.3.4.5 Well-being

Four studies focused on the outcome of well-being (68, 69, 72, 74).

Henshall et al. could show a trend towards improvement; however, results could not reach statistical significance (69). In another study, significant improvements in well-being were made when training was performed at least once (72). Grabbe et al. succeeded to significantly improve well-being over time (68). When at least one hour of training was done, significant increases in well-being could be seen in the study of Nguyen et al. (74).

The “Crew Resource Management” training, mindfulness and mind-body skills training interventions, and the “Community Resiliency Model” achieved a significant increase in well-being. The Resolution program, on the other hand, showed only a non-significant trend toward improvement.

Measurement tools included the Warwick-Edinburgh Mental Wellbeing Scale (69), Physician Well-Being Index (PWI) (72, 74), and the WHO-5 Well-being Index (WHO-5) (68).

A detailed table with the results for each outcome is located in the appendix (**Supplementary Table 11**).

##### 3.3.4.6 Correlations

Three studies investigated various variables and their interrelationships (74, 79, 83). One study found a correlation between the number of completed modules and changes in EE and well-being scores (74). Another study demonstrated a significant interaction between time and intervention regarding outcomes such as resilience, stress, depression, and anxiety (79). Additionally, a connection was observed between depression and burnout (83).

##### 3.3.4.7 Students

Three studies (79–81) reported an effect on stress (79, 81), burnout (79), and depression and anxiety (79, 80) through the Use of the Transition and Adaptation towards Resiliency module, the ThoughFullChat intervention, and the Compassion Cultivating Training.

**Figure 2:**
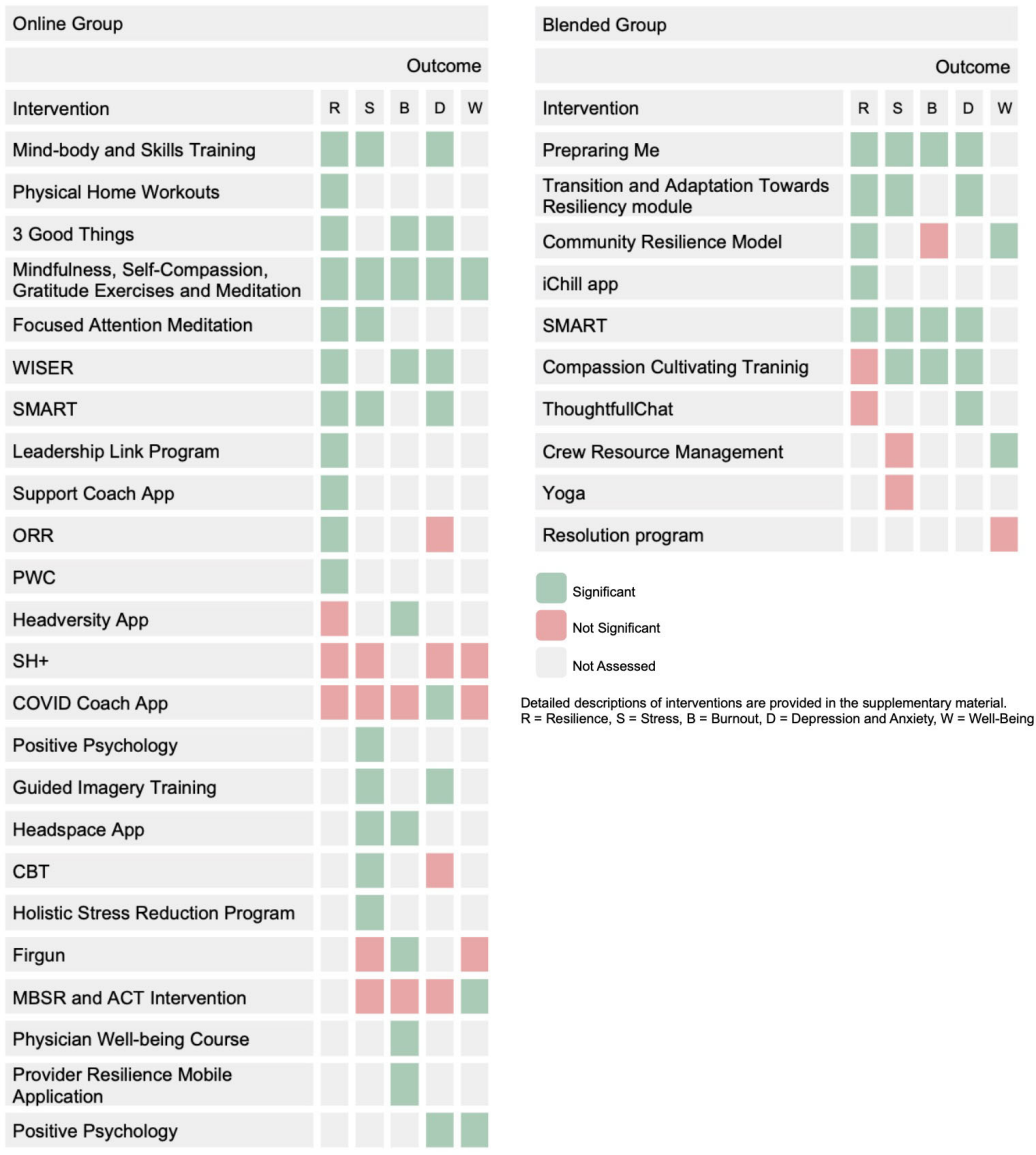
Outcome heatmap: online vs. blended group.

All outcomes of the included interventions, along with their reported significance, are visually summarized in the heatmap (**Figure 2**).

### 3.4 Risk of bias assessment

The Effect Public Health Practice Project (EPHPP) was used to assess the risk of bias.

Among the 55 included studies, only three studies achieved a strong global rating, while at least 12 attained a moderate global rating.

Regarding Selection Bias, all studies included a population likely to be representative of the target population, as this was an inclusion criterion from the outset. However, certain studies received a weak rating since only a specific portion of the initially approached individuals ultimately participated.

According to the inclusion criteria, all studies with an intervention design were included, regardless of whether they were randomized controlled trials (RCTs), uncontrolled trials, or pre-post intervention studies. As a result, only 23 studies received a strong rating in the study design section, as they were the only ones conducted as RCTs. The remaining studies, being either uncontrolled trials or using pre-post intervention designs, did not achieve the same rating.

Several studies were assessed as weak in the Confounders section, particularly if the study was designed with only one group, making an assessment in this regard impossible. The same applied to the Blinding section. In the Data Collection Methods, all studies demonstrated strength, as the use of a valid and quantitative measurement method was a criterion for inclusion. In the Dropouts section, one-third of all included studies had to be rated as weak.

Detailed results of the EPHPP are provided in the appendix (Effective Public Health Practice Project (EPHPP) Risk of bias assessment).

The three studies that achieved a strong global rating are summarized in the following table (**Table 10**).

**Table 10:** Studies with a strong global rating in the risk of bias assessment.

Title	Participants	Intervention	Time frame	Outcomes	Study design	Control group, blinding	Resilience	stress	Burnout	Depression, anxiety	Correlation
Impact of App-Delivered Mindfulness Meditation on Functional Connectivity, Mental Health, and Sleep Disturbances Among Physician Assistant Students: Randomized, Wait-list Controlled Pilot Study	N = 14, physician assistant students	Mobile app-delivered mindfulness meditation	1-year subscriptions to the app, program: 8 weeks. 12 min per day. Modules: 4–13 min each	Burnout (School Burnout Inventory 9 items), depression and anxiety (The Depression Anxiety and Stress Scale). Measured at baseline and > 8 weeks.	RCT	Yes (wait-list control). Study personnel was blinded to the group randomization	x	x	(1) No significant improvement in burnout after participating in the intervention.	(1) No significant improvements in depression or anxiety after completion of the intervention.	
Efficacy of the my health too online cognitive behavioral therapy program for healthcare workers during the COVID-19 pandemic: A randomized controlled trial.	N = 147, healthcare workers (nurses, nursing students, doctors, physiotherapists, midwives, psychologists)	Cognitive Behavioral therapy via the MyHealthToo app. The app consisted of 7 sessions: (1) psychoeducation, (2) functional behavioral and cognitive coping strategies, (3) mindfulness, (4) acceptance, (5) promoting action toward values, (6) addressing barriers and motivation to use self-compassion as a psychological gift, (7) sleep problems and problem-solving strategies.	8 weeks, 7 online sessions à 20 min each	Stress (Perceived Stress Scale - 10 items version (PSS-10)), depression (The Patient Health Questionnaire -2 items version (PHQ-2)), resilience (Connor-Davidson Resilience Scale - 2 items version (CD-RISC 2)). Measured pre-, mid- (4 weeks), post-treatment (8 weeks. Follow-up at 1 and 4 months.	RCT	yes (experimental vs. active control group (bibliotherapy)), single blinded (data manager was blinded to the allocation group	(1) No significant changes in resilience after the intervention.	(1) Significant stress reduction post-therapy at 8 weeks. (2) Significantly improved results in perceived stress at the 1-month follow-up (after 12 weeks). (3) Significantly reduced stress at the 4 months follow-up (after 24 weeks).	x	(1) No significant changes in depression after the intervention.	x
Guided self-help mindfulness-based intervention for increasing psychological resilience and reducing job burnout in psychiatric nurses: A randomized controlled trial.	N = 99, psychiatric nurses	Didactic information was delivered through audio files and text materials that were sent on WeChat on smartphones. The self-help mindfulness intervention aimed to guide participants to focus on the present moment and to learn how to practice in a non-judgmental fashion during stressful experiences. The program included didactic instructions and practice on mindfulness, concentration, and awareness. The practice included body scan, mindful walking, breathing meditation, and transposition exercise.	8 weeks, 5x per week, 20–30 min each time	Resilience (Connor-Davidson Resilience Scale), burnout (Maslach Burnout Inventory). Measured at baseline and after 4 and 8 weeks.	RCT	yes (psycho-educational brochure). Researchers and nurses were blinded to intervention allocation.	(1) Significant main effect of intervention, time, and a significant interaction between intervention and time. (2) No changes in resilience over time in the control group.	x	(1) Significant main effect of intervention, time, and a significant interaction between intervention and time. (2) No changes in burnout scores over time in the control group.	x	(1) Significant correlation between resilience and job burnout: score reduction of CD-RISC was negatively correlated with the score reduction of MBI.

All three studies were conducted as randomized controlled trials and performed moderately to strongly in the blinding section due to single or double blinding. A large proportion of the initially approached participants were recruited for the intervention and were likely to be representative of the target population, leading to strong performance in the selection bias section. Additionally, the attrition rate was low in all three studies, which was methodologically solid. Due to the relatively high number of participants in two out of these three studies, their findings are more robust and generalizable. In terms of content, the CBT intervention achieved a significant reduction in stress, while one of the two mindfulness interventions led to improvements in resilience and burnout.

## 4 Discussion

### 4.1 Summary of main findings

This systematic review screened existing literature on how to foster resilience in healthcare and finally included 55 studies that examined the effects of various online and blended interventions on resilience and related outcomes, designed as (randomized) controlled trials and cohort studies.

Overall, 7898 healthcare workers comprised of doctors, nurses, allied health personnel and healthcare students were able to receive interventions incorporating cognitive-behavioral therapy, acceptance and commitment therapy, mindfulness, mind-body skills, gratitude, meditation, leadership, support, positive psychology, guided imagery, and physical activities. Most studies reported immediate and significant improvements in various mental health outcomes, indicating that these resilience interventions have meaningful and lasting effects on well-being.

Some studies highlighted correlations between the frequency and time invested in training, and the extent of improvement in well-being, resilience, stress, and burnout. Furthermore, a relationship emerged between resilience and emotional distress, well-being, and empathy. This demonstrates how interconnected resilience is and how an improvement in it can influence other outcomes as well.

One strength of this review is the inclusion of student populations from diverse health disciplines. Findings suggest that fostering resilience during academic years may improve coping with stress in professional life, highlighting the interventions potential long-term benefits.

### 4.2 Comparison of blended *vs*. online group

Both studies that conducted interventions purely online and those that used a blended format were able to achieve significant results in the five outcomes examined.

A significant advantage of conducting the intervention purely online is that it allows participants to engage at their own pace and at a place and time of their choosing, seamlessly integrating into their individual schedules. Furthermore, there is no need to provide a location or instructors, which represents a financial advantage.

However, the added personal component in blended interventions was appreciated. For instance, participants appreciated the personal interaction and exchange with other participants. Furthermore, the instruction of interventions could be better explained, and any open questions were promptly addressed.

Generally, the comparatively higher attrition rate in pure online intervention studies (84–88) was also evident in this review where the blended group scored higher in the risk of bias assessment regarding withdrawals and dropouts compared to the online group.

In conclusion, both online and blended interventions significantly improve psychological outcomes, each offering distinct advantages. The choice of format should depend on factors like time constraints, location, needs, and costs.

### 4.3 Comparison with previous work

There has been previous work conducted regarding web-based resilience interventions in healthcare professions.

Three other systematic reviews were found that examined the effects of online interventions to enhance mental health in physicians, nurses, and allied healthcare staff.

Ladino et al. (20) conducted a systematic review involving four articles to examine the effects of internet-based psychosocial interventions on professional burnout. Despite a meta-analysis of two articles suggesting no significant reduction in professional burnout, and even though post-interventionally no significant differences compared to the control group could be observed, the review highlighted psychoeducation as a promising intervention for addressing this issue. It was also demonstrated in this review that psychoeducation is a way to improve mental health (29, 34, 41, 44, 45, 51, 55).

In a similar vein, Henshall et al. (26) conducted a systematic review encompassing eight studies, revealing a positive impact of web-based interventions on resilience and associated symptoms in health professionals in clinical practice settings. These results largely align with the findings of this review and illustrate the impact that online interventions can have not only on resilience, but on various psychological outcomes.

Further insights were gained from Lopez-Del-Hoyo et al. (27), who included 27 studies in their review to assess the effectiveness of eHealth interventions in reducing stress and promoting mental health. Thirteen studies reported a significant post-intervention reduction in stress, along with improvements in depression, anxiety, burnout, resilience, and mindfulness. Nevertheless, the study’s conclusions were constrained by the observed heterogeneity in the interventions. This study, which encompassed a similar volume of research, yielded comparable results to this review. However, what stood out with the inclusion of a larger number of studies, as observed in this review, is the challenge posed by the heterogeneity of interventions and measurement methods, highlighting the need for standardization to enhance comparability.

The present review contributes to the current research on interventions aimed at preserving psychological health across various healthcare professions to further progress in this rapidly evolving subject. The potential of these interventions was demonstrated through significant improvements in resilience, well-being, and the reduction of symptoms related to burnout, stress, depression, and anxiety.

### 4.4 Methodological quality

In the risk of bias assessment, only three out of the 55 included studies received a strong global rating. The three strong studies demonstrated that CBT and mindfulness interventions could improve resilience, stress, and burnout. Twelve studies achieved a moderate rating, while the remaining studies were rated as weak. Consequently, the findings from the majority of studies cannot be reliably analyzed, and even statistically significant results should be interpreted with serious caution.

Notably, all studies that received a strong rating were in the online group, suggesting that the results from the blended group are only partially reliable. This reduced reliability may stem from greater difficulties in implementing blinding for in-person components, the presence of confounding factors between groups, and selection bias.

The three studies with strong ratings achieved high methodological quality using randomized controlled trial (RCT) designs, single or double blinding, low attrition rates, and minimal selection bias. In contrast, the remaining studies faced various methodological issues. Although the study populations across all studies were representative of the target population according to the inclusion criteria, some studies struggled to recruit sufficient participants, with only a fraction of the initially approached individuals participating in the intervention and consequently received lower ratings. Additionally, some studies experienced higher attrition rates, further affecting their reliability.

The data collection methods were consistently rated as strong, as they met the criteria for inclusion. Regarding study designs, all intervention studies were included, with RCTs performing better than pre-post intervention designs. Blinding, particularly in psychological or behavioral interventions, was often not feasible due to the nature of the interventions, resulting in many studies receiving a weak rating in the blinding section. The lack of effective blinding may have contributed to performance bias, where various factors, such as participants’ awareness of the intervention or the mere attention from a professional, may influenced the participants’ behavior or reported outcomes. As a result, improvements cannot be clearly attributed to the intervention itself, complicating the assessment of its true efficacy. This raises the possibility that the strong effect sized reported in some studies may be inflated by placebo effects or participant expectations rather than reflecting the genuine impact of the intervention.

### 4.5 Limitations and implications for future research

Until today, there is no uniform definition of resilience. As a result, there are still no universally valid measurements methods for this. The included studies have used various tools to objectify resilience. Related symptoms such as stress, burnout, anxiety, and depression have been used as proxy indicators for resilience, which further complicates the interpretation.

This problem is accompanied by a further limitation. Due to the versatility of resilience, the included studies have implemented a variety of interventions with diverse content.

Additionally, the timing of measured changes following interventions varied significantly across the studies (post-intervention *vs*. follow-up). This variability suggests the possibility that some changes may have been missed if measurements were taken only after a certain period. While other studies may not provide insights into the long-term effects of the interventions. All this leads to challenges in the general interpretation and comparability of the studies. In the future, efforts should be made to establish a universally accepted definition of resilience and develop standardized, objective measurement tools. Such consensus is crucial to enhance the comparability of studies, particularly regarding the timing of assessments, which currently varies widely. A unified conceptual framework and consistent measurement would allow researchers to more accurately capture changes in resilience, compare intervention outcomes across different studies, and identify which interventions are most effective. Furthermore, standardized tools would facilitate meta-analyses and evidence synthesis, ultimately supporting the optimal design and tailoring of interventions specifically aimed at improving resilience in diverse populations.

An additional constraint to generalizability is the composition of the study populations. In the vast majority of studies, a significant proportion of participants are female. Given the findings of a recent study that approximately 77% of nurses worldwide are female (89), this is not surprising, considering that nurses constitute the largest proportion of all professions in this review. However, this gender distribution does not reflect all healthcare professions, many of which have a more balanced or different gender ratio. As a result, the findings of this review have limited generalizability across the broader range of healthcare workers and are even less applicable to the general population. To draw more robust and widely applicable conclusions about the effectiveness of resilience interventions, future studies should include samples that are more balanced and diverse in terms of gender and professional background, since intervention effects observed primarily in predominantly female nursing populations may not apply to other healthcare professions or demographic groups. Differences in occupational roles, stressors, and coping strategies could lead to varying outcomes, so caution is necessary when extrapolating these results beyond the studies groups.

Furthermore, several studies lacked a control group, which limits the conclusiveness regarding intervention effectiveness. Future studies should therefore be designed and conducted as randomized controlled trials.

The relatively high attrition rate is another limitation. Although many individuals could be initially reached, some were lost until follow-up surveys. Attrition is a well-known phenomenon especially in online interventions (90) and should be minimized in future studies.

Lastly, a significant limitation is the methodological quality constraints of many studies. Due to high attrition rates, selection bias, study designs, and challenges with blinding, only three studies achieved a strong global rating in the risk of bias assessment. Consequently, only the findings from these studies are considered decisive.

Overall, the previously mentioned heterogeneity among the studies continues to be a challenge, limiting full comparability.

To improve the validity of future studies, it is critical to address these methodological weaknesses to enhance the reliability and significance of their findings. Ensuring higher recruitment rates and lower attrition would help provide more representative results. Incorporating blinding techniques where feasible could reduce bias and lead to more reliable findings. Without these improvements, even statistically significant outcomes must be interpreted with caution, as they may be influenced by uncontrolled biases and limitations inherent in the study design.

Due to mentioned limitations, the results should be interpreted with caution until replicated.

### 4.6 Broader implications

As demonstrated in this review, numerous resilience interventions have shown a significant improvement in multiple mental health outcomes. This contributes to strengthening individual resilience and acts as a strategy against burnout symptoms and other manifestations related to stress.

Beyond structured training programs, there are various other individual-level workplace interventions that may contribute to strengthening resilience (91–96). These include, for example, employer-provided access to physical activity facilities such as on-site gyms, which may help employees enhance their mental well-being through exercise. Similarly, the availability of quiet or relaxation rooms can offer staff opportunities for mental rest and recovery during demanding workdays. Other supportive measures include designated contact persons or anonymous hotlines, which allow employees to express concerns and receive guidance in times of distress. However, such interventions tend to be reactive rather than preventive in nature and may lack the lasting effects that targeted resilience interventions aim to achieve. Moreover, their overall impact is often limited to the individual level and does not necessarily translate into broader organizational change.

However, as mentioned at the outset, there are organizational factors that cannot be solely influenced by individual actions and necessitate a broader approach. Employers and large institutions are thus encouraged to take measures, such as avoiding prolonged working hours, offering psychological support when needed, and emphasizing overall employee well-being as part of the institutional culture to contribute further to the preservation of the mental health of their workforce.

For future considerations, it is advisable to address the issue in two ways: firstly, through individual training sessions aimed at strengthening the resilience of individual persons, as seen in the included studies, and secondly, through organizational changes on the part of employers that can further positively influence mental health at the organizational level. Such measures may include implementing clear and manageable working hours, ensuring reasonable workload distribution, promoting supportive leadership and effective communication, providing access to psychological support services, and fostering a workplace culture that prioritizes employee well-being and work-life balance.

This review focused exclusively on assessing the impact of resilience interventions within healthcare personnel. It would be intriguing for future research to expand the investigation to the entire workforce, thereby revealing the potential for preserving and enhancing resilience and mental health, particularly in today’s world marked by pervasive shortages of skilled personal in various fields.

## 5 Conclusions

In summary, findings of this review suggest a positive impact of the analyzed interventions on overall resilience, consequently leading to a positive effect on symptoms of depression, anxiety, better coping with occupational stress, and a significant contribution to the maintenance and promotion of general well-being. The inclusion of long-term follow-up results also demonstrated the likely sustainability of the effects with a longer duration.

These comprehensive results emphasize the relevance of resilience promotion within interventions aimed at improving the mental health of healthcare professionals.

Future research is warranted to establish a universally applicable definition and measurement methodology for resilience, which would facilitate the development of more standardized interventions, promoting comparability across studies.

Further considerations for future studies include addressing limitations such as the composition of the study populations, study designs and attrition rates. Additionally, expanding the study population across diverse occupational domains is recommended to enhance the generalizability of findings. Moreover, institutional measures are imperative to contribute at the organizational level and address the issue comprehensively. Lastly, ensuring high methodological quality in future studies is essential to produce interpretable and meaningful results.

## Data Availability

The original contributions presented in the study are included in the article/**Supplementary Material**. Further inquiries can be directed to the corresponding author.
