# Supplementary material for: Digital resilience interventions for healthcare workers: a systematic review
Source: Front Psychiatry. 2025 Sep 10;16:1519670. doi: 10.3389/fpsyt.2025.1519670 (PMC12457347; doi:10.3389/fpsyt.2025.1519670)
Supplement: Supplementary file 1 [file Supplementaryfile1.docx]

Supplementary Material

# Resilience Definition and Measurement

Figure 1 Resilience definition and measurement

**RESILIENCE DEFINITION**

The definition of resilience has been a complex and evolving concept, with its understanding undergoing various changes over time. Despite considerable research efforts, there is still no universally accepted definition until today.

Numerous concepts of resilience have emerged, yet there is still disagreement which shows the diversity of resilience. The debate revolves around whether resilience is a state dynamically influenced by different factors and the environment, or if it is a trait in the sense of a stable characteristic of an individual.

So far, researchers generally agree on two key factors to understand resilience: facing a significant risk and experiencing positive growth despite the challenges (1).

The existing literature on resilience definitions has been summarized by Antonella Sisto et al. in a systematic review and categorized into five groups (2):

- Ability to recover
- Type of functioning that characterizes the individual
- Capacity to bounce back
- Dynamic process evolving over time
- Positive adaptation to life conditions

These five groups reflect the various aspects of resilience and highlight the versatility of this concept depending on the perspective from which it is analyzed. Based on all the definitions, they have outlined the following definition: «It can be affirmed that psychological resilience is the ability to adapt positively to life conditions. It is a dynamic process evolving over time that implies a type of adaptive functioning that specifically allows us to face difficulties by recovering an initial balance or bouncing back as an opportunity for growth.”

Further research is warranted to establish a universally valid, standardized definition of resilience.

**RESILIENCE MEASUREMENT**

Due to the lack of a uniform definition of resilience, the objective and universally applicable measurement of resilience is difficult. There are now many different methods that attempt to measure aspects of resilience, but none that has yet become fully established. This is reflected in the included studies, which use highly heterogeneous scales to objectify resilience.

To provide additional insight into resilience, one can select broader outcomes that may arise as a consequence of resilience or its absence.

In this work, resilience is used as a direct outcome, measured by several scales, and it is also indirectly described through resilience indicators. This study has focused on indicators such as well-being, stress, burnout, anxiety, and depression, as several studies have demonstrated their connection to resilience (3-13). Existing and enhanced resilience, for instance, is shown to lead to increased well-being. Conversely, studies have shown that reduced or absent resilience is associated with increased perceived stress, burnout, and symptoms of anxiety and depression.

# Search strategy

## Search terms used for literature search:

### PubMed:

*((Resilience) OR (“Resilience, Psychological”[Mesh])) AND ((online OR web OR web-based OR digital OR blended OR internet OR app OR smartphone OR mobile phone OR computer OR technolog* OR (multimedia OR multi-media) OR website OR e-learning OR (e-health OR electronic health)) AND (course OR training OR intervention OR promot* OR practice OR exerci* OR treatment OR therap* OR (“Internet-Based-Intervention”[Mesh]))) AND (healthcare OR health profession* OR doctor OR medical OR physician OR medical students OR nurses OR nursing students OR (“Physicians”[Mesh]) OR (“nurses”[Mesh]) OR (“Students, Medical”[Mesh]) OR (“Students, Nursing”[MESH]) OR (“Health Personnel”[Mesh])) NOT review NOT protocol NOT cross-sectional*

### Embase:

Resilience AND ((online OR web OR web-based OR digital OR blended OR internet OR app OR smartphone OR mobile phone OR computer OR technolog* OR (multimedia OR multi-media) OR website OR e-learning OR (e-health OR electronic health)) AND (course OR training OR intervention OR promot* OR practice OR exerci* OR treatment OR therap*)) AND (healthcare OR health profession* OR doctor OR medical OR physician OR medical students OR nurses OR nursing students) NOT systematic review NOT protocol NOT cross-sectional

### Web of Science:

Resilience AND ((online OR web OR web-based OR digital OR blended OR internet OR app OR smartphone OR mobile phone OR computer OR technolog* OR (multimedia OR multi-media) OR website OR e-learning OR (e-health OR electronic health)) AND (course OR training OR intervention OR promot* OR practice OR exerci* OR treatment OR therap*)) AND (healthcare OR health profession* OR doctor OR medical OR physician OR medical students OR nurses OR nursing students) NOT review NOT protocol NOT cross-sectional

## Reference studies used for training the AS Review algorithm:

### First search March 2023:

- decreasing stress and burnout in nurses: efficacy of blended learning with stress management and resilience training program. (<https://pubmed.ncbi.nlm.nih.gov/28727625/>)
- Impact of a Digital Intervention on Perceived Stress, Resiliency, Social Support, and Intention to Leave Among Newly Licensed Graduate Nurses: A Randomized Controlled Trial (<https://pubmed.ncbi.nlm.nih.gov/34324377/>)
- long-term beneficial effects of an online mind-body training program on stress and psychological outcomes in female healthcare providers: a non-randomized controlled study. (<https://pubmed.ncbi.nlm.nih.gov/32769863/>)
- the role of psychological variables in improving resilience: comparison of an online intervention with a face-to-face intervention. a randomised controlled clinical trial in students of health sciences. (<https://pubmed.ncbi.nlm.nih.gov/33540351/>)
- Acute Effects of Online Mind-Body Skills Training on Resilience, Mindfulness, and Empathy (<https://pubmed.ncbi.nlm.nih.gov/25783980/>)
- building personal resilience following an online resilience training program for bscn students. (https://pubmed.ncbi.nlm.nih.gov/33998338/)
- randomized controlled trial of the "wiser" intervention to reduce healthcare worker burnout (<https://pubmed.ncbi.nlm.nih.gov/34366432/>)
- Resilience Training for Work-Related Stress Among Health Care Workers: Results of a Randomized Clinical Trial Comparing In-Person and Smartphone-Delivered Interventions (<https://pubmed.ncbi.nlm.nih.gov/29370014/>)
- The effects of biofeedback training and smartphone-delivered biofeedback training on resilience, occupational stress, and depressive symptoms among abused psychiatric nurses (<https://pubmed.ncbi.nlm.nih.gov/32331460/>)
- An online mindfulness intervention for medical students in South Africa: A randomised controlled trial (<https://pubmed.ncbi.nlm.nih.gov/35747337/>)
- Effects of the Use of the Provider Resilience Mobile Application in Reducing Compassion Fatigue in Oncology Nursing (<https://pubmed.ncbi.nlm.nih.gov/27857259/>)
- Promoting resilience in healthcare workers during the COVID-19 pandemic with a brief online intervention (<https://pubmed.ncbi.nlm.nih.gov/34857369/>)
- Impact of a Blended Web-Based Mindfulness Programme for General Practitioners: a Pilot Study (<https://link.springer.com/article/10.1007/s12671-017-0752-8>)
- Implementation of a Web-Based Resilience Enhancement Training for Nurses: Pilot Randomized Controlled Trial. (<https://www.webofscience.com/wos/alldb/full-record/MEDLINE:36787181>)

### Second search April 2024:

- Feasibility, acceptability and preliminary efficacy of a mental health self-management app in clinicians working during the COVID-19 pandemic: A pilot randomised controlled trial (<https://pubmed.ncbi.nlm.nih.gov/37778231/>)
- A Smartphone App to Reduce Burnout in the Emergency Department: A Pilot Randomized Controlled Trial (<https://pubmed.ncbi.nlm.nih.gov/36373628/>)
- mHealth Gratitude Exercise Mindfulness App for Resiliency Among Neonatal Intensive Care Unit Staff: Three-Arm Pretest-Posttest Interventional Study. (<https://www-webofscience-com.ezproxy.uzh.ch/wos/alldb/full-record/MEDLINE:38363595>)
- Efficacy of the my health too online cognitive behavioral therapy program for healthcare workers during the COVID-19 pandemic: A randomized controlled trial. (<https://www-webofscience-com.ezproxy.uzh.ch/wos/alldb/full-record/MEDLINE:38617386>)
- Improving resilience and reducing stress in emergency medicine physicians and residents by online training: a pilot study. (<https://www-webofscience-com.ezproxy.uzh.ch/wos/alldb/full-record/MEDLINE:38661503>) (not published yet)
- Feasibility and acceptability of a culturally adapted psychological first aid training intervention (Preparing Me) to support the mental health and well-being of front-line healthcare workers in China: a feasibility randomized controlled trial (<https://pubmed.ncbi.nlm.nih.gov/38269751/>)

## Included studies with their ranking in the algorithm-based table:

### Results March 2023:

| Title | Rank Nr. |
| --- | --- |
| Online | |
| Long-term beneficial effects of an online mind-body training program on stress and psychological outcomes in female healthcare providers: A non-randomized controlled study. | 3 |
| Acute Effects of Online Mind-Body Skills Training on Resilience, Mindfulness, and Empathy | 5 |
| Building Personal Resilience following an Online Resilience Training Program for BScN Students. | 6 |
| Randomized controlled trial of the "WISER" intervention to reduce healthcare worker burnout. | 7 |
| Resilience Training for Work-Related Stress Among Health Care Workers: Results of a Randomized Clinical Trial Comparing In-Person and Smartphone-Delivered Interventions. | 8 |
| Promoting resilience in healthcare workers during the COVID-19 pandemic with a brief online intervention. | 11 |
| Brief Online Focused Attention Meditation Training: Immediate Impact. | 17 |
| What Is the Impact of Online Training in Mind-Body Skills? | 24 |
| Effectiveness of a bite-sized web-based intervention to improve healthcare worker wellbeing: A randomized clinical trial of WISER. | 36 |
| Online Training in Mind-Body Therapies: Different Doses, Long-term Outcomes | 50 |
| Impact of App-Delivered Mindfulness Meditation on Functional Connectivity, Mental Health, and Sleep Disturbances Among Physician Assistant Students: Randomized, Wait-list Controlled Pilot Study | 78 |
| Smartphone-based home workout program for shift-work nurses working during the COVID-19 pandemic. | 105 |
| Improving Healthcare Worker Resilience and Well-Being During COVID-19 Using a Self-Directed E-Learning Intervention. | 113 |
| Effectiveness of self-help plus (SH+) in reducing anxiety and post-traumatic symptomatology among care home workers during the COVID-19 pandemic: a randomized controlled trial. | 121 |
| A Smartphone App to Reduce Burnout in the Emergency Department: A Pilot Randomized Controlled Trial. | 150 |
| Evaluation of a Web-Based Holistic Stress Reduction Pilot Program Among Nurse-Midwives | 175 |
| Help in hand after traumatic events: a randomized controlled trial in health care professionals on the efficacy, usability, and user satisfaction of a self-help app to reduce trauma-related symptoms | 232 |
| The Nurse Empowerment Program for Nurses in Direct Care Positions | 305 |
| Can stoic training develop medical student empathy and resilience? A mixed-methods study. | 327 |
| Three good things: Promote work-life balance, reduce burnout, enhance reflection among newly licensed RNs | 350 |
| Reduction of Burnout in Mental Health Care Providers Using the Provider Resilience Mobile Application. | 358 |
| Well-being in Residency: Impact of an Online Physician Well-being Course on Resiliency and Burnout in Incoming Residents. | 1264 |
| Decreasing Burnout and Improving Work Environment: The Impact of <i>Firgun</i> on a Pediatric Hematopoietic Cell Transplant Team. | 1569 |
| The Effects of an Online Mind-Body Training Program on Stress, Coping Strategies, Emotional Intelligence, Resilience and Psychological State  Ref: Long-term beneficial effects of an online mind-body training program on stress and psychological outcomes in female healthcare providers | Reference |
| Exploring the effects of an online asynchronous mindfulness meditation intervention with nursing students on stress, mood, and cognition: a descriptive study  Ref: Long-term beneficial effects of an online mind-body training program on stress and psychological outcomes in female healthcare providers | Reference |
| Building personal resilience in paramedic students  Ref: Building Personal Resilience following an Online Resilience Training Program for BScN Students | Reference |
| Building personal resilience in primary care paramedic students, and subsequent skill decay  Ref: Building Personal Resilience following an Online Resilience Training Program for BScN Students | Reference |
| Forty-five good things: a prospective pilot study of the Three Good Things well-being intervention in the USA for healthcare worker emotional exhaustion, depression, work–life balance and happiness  Ref: Randomized controlled trial of the “WISER” intervention to reduce healthcare worker burnout | Reference |
| The Feasibility and Effectiveness of Online Guided Imagery Training for Health Professionals  Ref: Brief Online Focused Attention Meditation Training: Immediate Impact | Reference |
| Effectiveness of an online positive psychology intervention among Tunisian healthcare students on mental health and study engagement during the Covid-19 pandemic | 23 |
| Blended |  |
| Decreasing Stress and Burnout in Nurses: Efficacy of Blended Learning With Stress Management and Resilience Training Program. | 1 |
| Impact of a Blended Web-Based Mindfulness Programme for General Practitioners: a Pilot Study | 12 |
| Implementation of a Web-Based Resilience Enhancement Training for Nurses: Pilot Randomized Controlled Trial. | 13 |
| The impact of Stress Management and Resailience Training (SMART) on academic physicians during the implementation of a new Health Information System: An exploratory randomized controlled trial. | 19 |
| Mind-Body Skills Training for Resident Wellness: A Pilot Study of a Brief Mindfulness Intervention. | 33 |
| Interventions to reduce burnout and improve resilience: Impact on a health system's outcomes | 68 |
| Sustained resiliency building and burnout reduction for healthcare professionals via organizational sponsored mindfulness programming. | 77 |
| The Community Resiliency Model¬Æ to promote nurse well-being. | 174 |
| Brief video-module administered mindfulness program for physicians: a pilot study  Ref: Long-term beneficial effects of an online mind-body training program on stress and psychological outcomes in female healthcare providers | Reference |
| Training on mind-body skills: Feasibility and effects on physician mindfulness, compassion, and associated effects on stress, burnout, and clinical outcomes  Ref: Interventions to Reduce Burnout and Improve Resilience: Impact on a Health System’s Outcomes | Reference |

### Results April 2024:

| Title | Rank Nr |
| --- | --- |
| Online | |
| Leadership Link: Evaluation of an Online Leadership Curriculum for Certified Midwives and Certified Nurse-Midwives | 444 |
| Brief tele-mindfulness-based intervention: A multicenter randomized controlled trial. | 71 |
| A Mindfulness-Based Intervention for Acute Care Nursing Staff: A Pilot Study | 27 |
| Impact of an online training tool on individual and organizational resilience and mindfulness among radiological personnel in Norway | 24 |
| Feasibility, acceptability and preliminary efficacy of a mental health self-management app in clinicians working during the COVID-19 pandemic: A pilot randomised controlled trial | 1 |
| mHealth Gratitude Exercise Mindfulness App for Resiliency Among Neonatal Intensive Care Unit Staff: Three-Arm Pretest-Posttest Interventional Study. | 3 |
| Efficacy of the my health too online cognitive behavioral therapy program for healthcare workers during the COVID-19 pandemic: A randomized controlled trial. | 4 |
| Improving Resiliency in US Air Force Healthcare Personnel: A Randomized Preventive Trial | 49 |
| Guided self-help mindfulness-based intervention for increasing psychological resilience and reducing job burnout in psychiatric nurses: A randomized controlled trial. | 8 |
| Blended | |
| Effects of the Brief Simha Kriya Breathing Practice for Health Care Workers During the COVID-19 Pandemic. | 34 |
| A comparative study of well-being, resilience, mindfulness, negative emotions, stress, and burnout among nurses after an online mind-body based intervention during the first COVID-19 pandemic crisis | 26 |
| A compassion-based program to reduce psychological distress in medical students: A pilot randomized clinical trial | 15 |
| Effectiveness of an online mental health strengthening module to build resilience and overcome stress for transitional aged medical students | 16 |
| Feasibility and acceptability of a culturally adapted psychological first aid training intervention (Preparing Me) to support the mental health and well-being of front-line healthcare workers in China: a feasibility randomized controlled trial | 6 |
| Efficacy of a Text-Based Mental Health Coaching App in Improving the Symptoms of Stress, Anxiety, and Depression: Randomized Controlled Trial | 13 |

## Screening protocol:

1. Is the study written in German or English?

- Include, if yes.
- Exclude, if no.

2) Title and Abstract screening:

- What study design is used?
  - Include, if a controlled trial, randomized controlled trial, pre-post interventional design is used.
  - Exclude, if a study protocol, review, cross-sectional design is used.
- Who is the target population?
  - Include, if the target population consists of any healthcare professionals or students thereof.
  - Exclude, if the target population consists of any other profession.
- Which outcomes are measured and how?
  - Include, if resilience, stress, burnout, depression, anxiety ore wellbeing are measured using a quantitative measurement method.
  - Exclude, if none of the above-mentioned factors are measured or if no quantitative measurement is applied.
- What is the delivery of the intervention?
  - Include, if the intervention is completely asynchronous and online delivered.
  - Include in the second group, if the intervention is delivered in a blended format and contains in-person and online aspects.
  - Exclude, if the intervention is delivered in person or a remote format is used.

1. Full text screening

- If the above questions could not be fully answered based on the title or abstract alone.

# Outcomes

## Online group:

Table 10 detailed outcomes of the online group

| Title | Intervention | Measurement tool | Timepoint of assessment | Results | |
| --- | --- | --- | --- | --- | --- |
| Resilience | | | | | |
| Long-term beneficial effects of an online mind-body training program on stress and psychological outcomes in female healthcare providers: A non-randomized controlled study. | Mind-body training content and skills | The Korean version Connor-Davidson Resilience Scale (CDRS) | Baseline  8 weeks (post-intervention)  12 weeks (1 month after completion of training) | (1) Significant Time x group interaction found for the total resilience score.  (2) Greater increase in total resilience score in the MBT group that sustained over 1 month time. However, significant results achieved only for 1 subscale (strength for overcoming stress).  (3) Significant time x group across time found for T0-T1 and T0 bis T2. | |
| Acute Effects of Online Mind-Body Skills Training on Resilience, Mindfulness, and Empathy | Mind-body skills training; Curriculum: Mind-Body Skills Training for Resilience, Effectiveness, and Mindfulness | Smith's 6-item Brief Resilience Scale | at the beginning and end of each module | (1) Introduction to Stress and Resilience: Non-significant improvement  (2) Mindfulness in daily life: significant improvement | |
| Building Personal Resilience following an Online Resilience Training Program for BScN Students. | ORR = Online Resilience Resource (self-guided online training program which includes readings, videos, exercises, self-assessment tools) | Resilience Scale for Adults (RSA) | Baseline  1 month post intervention  3 months post intervention | (1) Significant improvement in resilience from baseline to post-training follow-up assessment.  (2) Significant improvement from baseline to 1 month post-intervention.  (3) Significant improvement from baseline to 3 months post-intervention. | |
| Promoting resilience in healthcare workers during the COVID-19 pandemic with a brief online intervention. | Online resilience-enhancement course focused on mindfulness, mentalization and self-compassion. | Brief Resilience Scale (BRS) | Baseline  1 month post-intervention  2 months post-intervention | (1) No significant changes in resilience levels from baseline to 1-month post-intervention in those (WHO completed the RT course and all 3 surveys.  (2) Significant change in resilience levels from baseline to 2-months post-intervention following the resilience training.  (3) Significant and sustained improvements in resilience were seen after 2 months in intervention completers compared to those who did not participate in the intervention. | |
| Brief Online Focused Attention Meditation Training: Immediate Impact. | Online focused attention meditation training | Smith's 6-item Brief Resilience Scale, Numeric rating scales (0-10) for resilience | Before and after a module | (1) Resilience on a numeric scale: Significant improvement between pre- and post-intervention. | |
| What Is the Impact of Online Training in Mind-Body Skills? | Mind-Body Skills training (focused attention meditation, mindfulness meditation, positive affect meditation, guided imagery/hypnosis) | Smith's 6-item Brief Resilience Scale | Baseline and 12 weeks post-intervention | (1) No significant differences in resilience score between groups. | |
| Effectiveness of a bite-sized web-based intervention to improve healthcare worker wellbeing: A randomized clinical trial of WISER. | WISER intervention (Web-based Implementation for the Science of Enhancing Resilience): a positive psychology program | numeric scales | Baseline, postintervention (1 week, 1 month, 6 months, 12 months) | (1) Significant improvements in emotional thriving at all timepoints.  (2) Significant improvements in emotional recovery at all timepoints. | |
| Online Training in Mind-Body Therapies: Different Doses, Long-term Outcomes | Mind-Body Skills Training for Resilience, Effectiveness, and Mindfulness | Smith's 6-item Brief Resilience Scale | Baseline, postintervention, 12 weeks postintervention | Mind-Body Skill practice frequency was associated with resilience levels. | |
| Smartphone-based home workout program for shift-work nurses working during the COVID-19 pandemic. | Smartphone-based home workouts | Connor-Davidson Resilience Scale (CD-RISC) | Baseline, week 6, 12, 18 | (1) Significant group-by-time difference that was increased in the intervention group and remained the same in the control group.  (2) Non-significant differences between groups in changes in resilience.  (3) Significant improvements over time in the intervention group.  (4) Non-significant differences in resilience between those who exercise 3-5 days a week and those who do less or stopped exercise. | |
| Improving Healthcare Worker Resilience and Well-Being During COVID-19 Using a Self-Directed E-Learning Intervention. | Psychological well-being, self-care (Physical, Mind, Relationships, Emotions, Work), mindfulness and meditation | Connor-Davidson Resilience Scale (CD-RISC) | Pre- and post-training | (1) Significant increase in resilience between pre- and posttraining. | |
| Effectiveness of self-help plus (SH+) in reducing anxiety and post-traumatic symptomatology among care home workers during the COVID-19 pandemic: a randomized controlled trial. | SH+: an individual self-help audio-visual tool: Doing what matters in times of stress. 5 core components: grounding, unhooking, acting on your values, being kind, making room. | Connor-Davidson Resilience Scale (CD-RISC) | Baseline  week 7 (1 week after training completion)  week 20 (14 weeks after training completion) | (1) No significant improvement in resilience at post-intervention or at follow-up. | |
| A Smartphone App to Reduce Burnout in the Emergency Department: A Pilot Randomized Controlled Trial. | Resilience curriculum on 6 domains:  mindfulness, self-expertise, mental fitness, mental health, hardiness, energy management | Connor-Davidson Resilience Scale (CD-RISC) | Pre- and post-training | (1) Non-significant improvement post-intervention in resilience scores. No changes at all in the control group. | |
| Help in hand after traumatic events: a randomized controlled trial in health care professionals on the efficacy, usability, and user satisfaction of a self-help app to reduce trauma-related symptoms | SUPPORT COACH app: psychoeducation about trauma, PTSS, professional care; support section to facilitate contact with one's personal network and professional care; self-test section: PTSD checklist for DSM-5; calendar section; manage symptoms section: exercises to self-manage PTSS | Resilience Evaluation Scale (RES) | Baseline (T1)  postintervention (T2)  1 month follow-up (T3) | (1) Significant difference in total resilience changes between groups. The intervention group showed a greater increase in resilience than the control group from T1 to T3.  (2) No significant differences between groups from T1 to T2.  (3) Significant increase in resilience from T1 to T2 and from T1 to T3 in the intervention group, but not in the control group. | |
| The Nurse Empowerment Program for Nurses in Direct Care Positions | The Nurse Empowerment Program: LinkedIn learning, nursing continuing professional development, critical conversations, asynchronous discussion platform | Connor-Davidson Resilience Scale (CD-RISC) 2-items | Baseline  post-intervention  3 months follow-up | (1) Significant increase in resilience between pre- and posttest.  (2) No significant differences between the follow-up score and the pretest or posttest scores. | |
| Can stoic training develop medical student empathy and resilience? A mixed-methods study. | Stoicism informed online training package: guided reflective diary | Brief Resilience Scale (BRS) | pre- and postintervention (semi-structured interviews posttest and after 2 months) | (1) Significant increase in resilience after completion of the intervention. | |
| Three good things: Promote work-life balance, reduce burnout, enhance reflection among newly licensed RNs | 3 good things - regarding joys, work, self-efficacy, self-care, relationships | 8-item Resilience scales (emotional thriving, emotional recovery) | Baseline  Posttest  6-months follow-up | (1) Significant improvement in emotional thriving between baseline and T1.  (2) Improvements made at T1 in emotional thriving decreased by T2.  (3) Significant improvement in emotional recovery at T2, but not at T1. BUT: When only participants that participated for 14 days were included, evidence was weaker. | |
| Reduction of Burnout in Mental Health Care Providers Using the Provider Resilience Mobile Application. | The Provider Resilience (PR) mobile app includes 2 assessment tools to help increase self-awareness of current levels of burnout: Professional Quality of Life Scale and the Burnout Visual Analog Scale. The app screen provides an overall graphic of the user's current resilience rating, customizable resilience builders and killers (encourages to be aware of factors that in-/decrease their resilience). Tools to help enhance resilience and reduce burnout, f.e. humorous cartoons, physical exercises, inspirational cards with motivational quotes), videos of consumers indication how their treatment impacted their lives, and video information on compassion fatigue. | Connor-Davidson Resilience Scale (CD-RISC) | Baseline  after 1 month | (1) No significant improvements in resilience. | |
| Well-being in Residency: Impact of an Online Physician Well-being Course on Resiliency and Burnout in Incoming Residents. | Physician Well-being Course (PWC): well-being (resilience, sleep, nutrition, mindfulness, exercise); resilience activities (gratitude, meditation, finding meaning) | Dispositional Resilience Scale  Connor-Davidson Resilience Scale (CD-RISC) | Pre- and postcourse | (1) Significant improvements in resilience scores. (CD-RISC)  (2) Significant improvements seen in dispositional resiliency (personality hardiness). | |
| The Effects of an Online Mind-Body Training Program on Stress, Coping Strategies, Emotional Intelligence, Resilience and Psychological State | MBT on resilience, coping strategies, anger, stress, emotional intelligence, positive/negative affect. Movement-based meditation. | Connor-Davidson Resilience Scale (CD-RISC) | Baseline  4 weeks and 8 weeks following onset | (1) Significant time x group interaction in resilience: Intervention group significantly improved in resilience scores over time compared to the control group. | |
| Building personal resilience in paramedic students | Online resiliency training program | Resilience Scale (RS) | Pre- and postintervention | (1) Significant improvement in total resilience and each of the sub scores except meaningfulness. | |
| Building personal resilience in primary care paramedic students, and subsequent skill decay | ORR: Online Resilience Resource | The Resilience Scale for Adults (RSA) | Baseline  3 /6 /9 months (three groups): all three phases of the study: n=34, 3-months: n=8, 6-months: n=20, 9-months: n=6 | (1) Slight, non-significant improvement in resilience from baseline to 3-months follow-up.  (2) Significant decrease in resilience scores at 6 or 9 months compared to baseline assessment.  GROUPS: **Completion of all three phases** (baseline, intervention, follow-up): Statistically significant reduction in resilience scores from baseline to follow-up. **3-months:** Non-significant improvement in resilience from baseline to follow-up at 3 months. **6-months:** Significant decrease in resilience from baseline to follow-up. **9-months**: Statistical decrease in resilience from baseline to follow-up. | |
| Leadership Link: Evaluation of an Online Leadership Curriculum for Certified Midwives and Certified Nurse-Midwives | The Leadership Link Program is an educational platform that aims to build leadership capacity and consists of 2 components: (1) 10 LinkedIn Learning leadership courses; (2) associated ACNM (American College of Nurse-Midwives) videos. The program improves leadership knowledge and skills to work effectively. Leadership domains: Understanding the context, professional expertise, self-awareness and self-development, communication as a change agent, operationalization and execution, transforming the future of midwifery. | Connor-Davidson Resilience Scale 2-item version (CD-RISC 2) | program use data throughout the program period, preprogram, immediately postprogram, 3 months follow-up | (1) Statistically significant improvements in CD-RISC2 from pre- to postprogram.  (2) Statistically significant improvements in resilience from preprogram to follow-up.  (3) No statistically significant difference between postprogram to follow-up. | |
| Brief tele-mindfulness-based intervention: A multicenter randomized controlled trial. | Brief Mindfulness-based interventions (MBI) and progressive muscle relaxation (PMR). MBI: 7 mediation commands. PMR: tensions and relaxing specific muscle groups in sequence. | Connor-Davidson Resilience Scale-10 (CD-RISC 10) | Pre- and postintervention | (1) No significant improvement in resilience. | |
| A Mindfulness-Based Intervention for Acute Care Nursing Staff: A Pilot Study | Mindfulness-based: Headspace consisted of modules with different meditations, Insight Timer comprised guided meditation with topics like presence and grounding awareness. | Brief Resilience Scale (BRS) | Preintervention  midpoint (after 15 days)  immediately postintervention (after 30 days) | (1) No statistically significant chances in resilience across the three timepoints. | |
| Impact of an online training tool on individual and organizational resilience and mindfulness among radiological personnel in Norway | Mindfulness and resilience training with 3 resilience exercises three good things, upside of stress and self-compassion), 3 mindfulness exercises (breath as anchor, body-scan, and walking meditation. Background Information on mindfulness, stress and stress management was delivered. | Connor-Davidson Resilience Scale (CD-RISC)  The Benchmark Resilience Tool | Pre- and postintervention | (1) Statistically significant lower resilience scores at follow-up compared to baseline in individual resilience. | |
| Feasibility, acceptability and preliminary efficacy of a mental health self-management app in clinicians working during the COVID-19 pandemic: A pilot randomised controlled trial | COVID Coach: self-management app comprising the following topics: Manage stress, Learn, Mood check, and find resources. These courses teach how to manage with mental wellbeing using relaxation and mindfulness exercises, deliver psychoeducational information and support by offering help links. | The Connor-Davidson Resilience Scale (CD-RISC) | Pre- and postintervention | (1) Non-significant improvement in resilience from pre to postintervention. | |
| Efficacy of the my health too online cognitive behavioral therapy program for healthcare workers during the COVID-19 pandemic: A randomized controlled trial. | Cognitive Behavioral therapy via the online MyHealthToo app. The app consisted of 7 sessions: (1) psychoeducation, (2) functional behavioral and cognitive coping strategies, (3) mindfulness, (4) acceptance, (5) promoting action toward values, (6) addressing barriers and motivation to use self-compassion as a psychological gift, (7) sleep problems and problem-solving strategies. | Connor-Davidson Resilience Scale - 2 items version (CD-RISC 2) | Pretreatment  mid-treatment (4 weeks), post-treatment (8 weeks)  1 month and 4 months follow-up | (1) No significant changes in resilience after the intervention. | |
| Improving Resiliency in US Air Force Healthcare Personnel: A Randomized Preventive Trial | Stress Management and Resilience Training (SMART). Online Training with 4 modules | Connor-Davidson-10 Resilience Scale (CD-RISC 10) | Baseline  at 12, 18, and 24 weeks | (1) Significant improvements in resilience were seen at all timepoints (12w, 18w, 24w).  (2) No significant differences between groups (online vs. In-person) | |
| Guided self-help mindfulness-based intervention for increasing psychological resilience and reducing job burnout in psychiatric nurses: A randomized controlled trial. | The self-help mindfulness intervention aimed to guide participants to focus on the present moment and to learn how to practice in a non-judgmental fashion during stressful experiences. The program included didactic instructions and practice with topics like Mindfulness, Concentration, and Awareness in Sports. It comprised principles of identifying habitual reaction patterns and cultivating non-judgmental awareness and acceptance of the present. The practice included body scan, mindful walking, breathing meditation, and transposition exercise. | Connor-Davidson Resilience Scale (CD-RISC) | Baseline  after 4 and 8 weeks | (1) Significant main effect of intervention, time, and a significant interaction between intervention and time.  (2) No changes in resilience over time in the control group. | |
| Stress | | | | | |
| Long-term beneficial effects of an online mind-body training program on stress and psychological outcomes in female healthcare providers: A non-randomized controlled study. | Mind-body training content and skills | The Korean Occupational Stress Scale (KOSS), Stress Response Inventory | Baseline  8 weeks (post-intervention)  12 weeks (1 month after completion of training) | (1) Occupational stress: no time x group interaction effect.  (2) Significant main effect of time (stress increased in both groups).  (3) No significant main effect of group (no difference between groups at baseline or follow-up).  (4) Significant time x group interaction in total stress when comparing T0 vs. T1/ T2 (MBT group with a greater decrease in stress response than the control group). | |
| Acute Effects of Online Mind-Body Skills Training on Resilience, Mindfulness, and Empathy | Mind-body skills training; Curriculum: Mind-Body Skills Training for Resilience, Effectiveness, and Mindfulness | Perceived Stress Scale | At the beginning and end of each module | (1) Introduction to Stress and Resilience: significant reduction in stress.  (2) Autogenic training: Perceived Stress Scale: Significant improvement | |
| Resilience Training for Work-Related Stress Among Health Care Workers: Results of a Randomized Clinical Trial Comparing In-Person and Smartphone-Delivered Interventions. | Mindfulness-based resilience training. Smartphone app: sleep, happiness and positivity, energy and focus, productivity; mindfulness and feeling less stressed. | Depression, Anxiety, and Stress Scale (DASS-21) | Baseline  6-weeks (after completion of intervention)  3-months post-intervention | (1) MBRT group: Significant decrease in stress after 6 weeks and 3 months.  (2) Smartphone group: Non-significant decrease in stress at 6 weeks and 3 months.  (3) control group: Non-significant decrease in stress at 6 weeks and 3 months. | |
| Brief Online Focused Attention Meditation Training: Immediate Impact. | Online focused attention meditation training | 10-item Perceived Stress Scale, Numeric rating scales (0-10) for stress | Before and after a module | Average PSS baseline scores were already high.  (1) Significant reduction in stress between pre- and post-intervention. | |
| What Is the Impact of Online Training in Mind-Body Skills? | Mind-Body Skills training (focused attention meditation, mindfulness meditation, positive affect meditation, guided imagery/hypnosis) | Cohen's 10-item Perceived Stress Scale | Baseline  12 weeks post-intervention | (1) Significant decrease in perceived stress after completion of MBS training. Increase in stress in those who did not engage in MBS. | |
| Online Training in Mind-Body Therapies: Different Doses, Long-term Outcomes | Mind-Body Skills Training for Resilience, Effectiveness, and Mindfulness | Cohen's Perceived Stress Scale (PSS) | Baseline  Postintervention  12 weeks postintervention | Negative association between the frequency of mind-body practice and perceived stress. Perceived Stress was associated with work missed in the past 30 days. | |
| Effectiveness of self-help plus (SH+) in reducing anxiety and post-traumatic symptomatology among care home workers during the COVID-19 pandemic: a randomized controlled trial. | SH+: an individual self-help audio-visual tool: Doing what matters in times of stress. 5 core components: grounding, unhooking, acting on your values, being kind, making room. | Perceived Stress Scale 10-item | Baseline  week 7 (1 week after training completion)  week 20 (14 weeks after training completion) | (1) No significant improvement in stress at post-intervention or at follow-up. | |
| Evaluation of a Web-Based Holistic Stress Reduction Pilot Program Among Nurse-Midwives | Web-based holistic stress reduction program using yoga, mindfulness-based stress reduction techniques and meditation | Perceived Stress Scale | Pre- & postintervention | (1) 25% reduction in the perceived stress score. | |
| Decreasing Burnout and Improving Work Environment: The Impact of <i>Firgun</i> on a Pediatric Hematopoietic Cell Transplant Team. | Meaningful recognition: creating a culture of recurring altruistic complements related to job performance. | Perceived Stress Scale | Baseline  post completion (after 8 weeks) | (1) No significant changes in perceived stress measured. | |
| The Effects of an Online Mind-Body Training Program on Stress, Coping Strategies, Emotional Intelligence, Resilience and Psychological State | MBT on resilience, coping strategies, anger, stress, emotional intelligence, positive/negative affect. Movement-based meditation. | Korean Occupational Stress Scale (KOSS) | Baseline  4 weeks and 8 weeks following onset | (1) Significant time x group interaction in stress: Intervention group significantly reduced stress scores over time compared to the control group. | |
| Exploring the effects of an online asynchronous mindfulness meditation intervention with nursing students on stress, mood, and cognition: a descriptive study | Online mindfulness intervention: Mindfulness-based stress reduction (MBSR) model components | Perceived Stress Scale (PSS) | Baseline  After 8 weeks, 24 weeks | (1) Significant reduction in perceived stress.  (2) Correlation between stress reduction and training frequency: the more practice, the greater the reduction in stress. | |
| The Feasibility and Effectiveness of Online Guided Imagery Training for Health Professionals | Brief online guided imagery training | Perceived Stress Scale | Pre- and postintervention | (1) Significant stress reduction following completion of the autogenic training module. | |
| Effectiveness of an online positive psychology intervention among Tunisian healthcare students on mental health and study engagement during the Covid-19 pandemic | The CARE program: Coherence, Attention, Relationship, Engagement | The Depression, Anxiety and Stress Scales (DASS-21) | Baseline  immediately postintervention  3 months follow-up | (1) Significant change in stress between pre- and posttest.  (2) No significant differences between posttest and follow-up that indicates that achieved effects could be maintained.  (3) No significant changes over time could be seen in the control group. | |
| A Mindfulness-Based Intervention for Acute Care Nursing Staff: A Pilot Study | Mindfulness-based: Headspace consisted of modules with different meditations, Insight Timer comprised guided meditation with topics like presence and grounding awareness. | Perceived Stress Scale | Preintervention  at midpoint (after 15 days)  immediately postintervention (after 30 days) | (1) Significant reductions in stress from baseline to both midpoint and follow-up. | |
| Feasibility, acceptability and preliminary efficacy of a mental health self-management app in clinicians working during the COVID-19 pandemic: A pilot randomised controlled trial | COVID Coach: self-management app comprising the following topics: Manage stress, Learn, Mood check, and find resources. These courses teach how to manage with mental wellbeing using relaxation and mindfulness exercises, deliver psychoeducational information and support by offering help links. | The Perceived Stress Scale | Pre- and postintervention | (1) Non-significant reduction in stress after completion of the intervention. | |
| Efficacy of the my health too online cognitive behavioral therapy program for healthcare workers during the COVID-19 pandemic: A randomized controlled trial. | Cognitive Behavioral therapy via the online MyHealthToo app. The app consisted of 7 sessions: (1) psychoeducation, (2) functional behavioral and cognitive coping strategies, (3) mindfulness, (4) acceptance, (5) promoting action toward values, (6) addressing barriers and motivation to use self-compassion as a psychological gift, (7) sleep problems and problem-solving strategies. | Perceived Stress Scale - 10 items version (PSS-10) | Pretreatment  mid-treatment (4 weeks), post-treatment (8 weeks)  1 month and 4 months follow-up | (1) Significant stress reduction post-therapy at 8 weeks.  (2) Significantly improved results in perceived stress at the 1-month follow-up (after 12 weeks).  (3) Significantly reduced stress at the 4 months follow-up (after 24 weeks). | |
| Improving Resiliency in US Air Force Healthcare Personnel: A Randomized Preventive Trial | Stress Management and Resilience Training (SMART). Online Training with 4 modules | Perceived Stress Scale (PSS) | Baseline  at 12, 18, and 24 weeks | (1) Significant reductions in perceived stress at all timepoints (12w, 18w, 24w).  (2) No significant differences between groups (online vs. In-person). | |
| Burnout | | | | | |
| Randomized controlled trial of the "WISER" intervention to reduce healthcare worker burnout. | 6 guided well-being modules | Maslach Burnout Inventory (5-item derivative of the emotional exhaustion scale) | Baseline  1 month post, 6 months post | | Primary outcome.  (1) Cohort 1: Significant reduction in EE scores compared to cohort 2.  (2) After 1 month, WISER was still associated with a significant decrease in emotional exhaustion in cohort 1. WISER was associated with lower emotional exhaustion at 1 month in cohort 2.  (3) At 6 months, effects of WISER on burnout still were significant and showed a similar pattern to 1-month post-intervention results in cohort 1.  (4) Combined cohort analyses: WISER significantly improved emotional exhaustion at 1 month and at 6 months. |
| Resilience Training for Work-Related Stress Among Health Care Workers: Results of a Randomized Clinical Trial Comparing In-Person and Smartphone-Delivered Interventions. | Mindfulness-based resilience training. Smartphone app: sleep, happiness and positivity, energy and focus, productivity; mindfulness and feeling less stressed. | MBI-Human Services Survey (MBI-HSS) | Baseline  6-weeks (after completion of intervention)  3-months post-intervention | | MBRT GROUP:  (1) EE: significant improvements at 6 weeks and 3 months.  (2) DP: non-significant improvements.  (3) PA: Non-significant improvements.  SMARTPHONE GROUP:  (1) EE: Non-significant improvements.  (2) DP: Non-significant improvements.  (3) PA: Non-significant improvements. CONTROL GROUP: Non-significant improvements. |
| Brief Online Focused Attention Meditation Training: Immediate Impact. | Online focused attention meditation training | Maslach Burnout Inventory (EE, DP, PA) | Before and after a module | | (1) 60% met at least one criterion for burnout. **No follow-up data**. |
| Effectiveness of a bite-sized web-based intervention to improve healthcare worker wellbeing: A randomized clinical trial of WISER. | WISER intervention (Web-based Implementation for the Science of Enhancing Resilience): a positive psychology program | Maslach Burnout Inventory (MBI) | Baseline  postintervention (1 week, 1 month, 6 months, 12 months) | | EE:  (1) Significant reductions at all timepoints in the combined cohort analysis. |
| Online Training in Mind-Body Therapies: Different Doses, Long-term Outcomes | Mind-Body Skills Training for Resilience, Effectiveness, and Mindfulness | 7-item Mayo Clinic Physician Well-Being Index (PWBI) (assesses burnout) | Baseline  Postintervention  12 weeks postintervention | | Negative association between the frequency of mind-body practice and perceived stress which was strongly correlated with burnout. |
| Impact of App-Delivered Mindfulness Meditation on Functional Connectivity, Mental Health, and Sleep Disturbances Among Physician Assistant Students: Randomized, Wait-list Controlled Pilot Study | Mobile app-delivered mindfulness meditation | School Burnout Inventory (9 items) | Baseline  >8 weeks | | (1) No significant improvement in burnout after participating in the intervention. |
| A Smartphone App to Reduce Burnout in the Emergency Department: A Pilot Randomized Controlled Trial. | Resilience curriculum on 6 domains:  mindfulness, self-expertise, mental fitness, mental health, hardiness, energy management | Emotional Exhaustion subscale of Maslach Burnout Inventory | Pre- and postintervention | | (1) Significant decrease in emotional exhaustion. No significant reduction in the control group.  (2) Non-significant decrease in DP in the intervention group and the control group.  (3) Non-significant improvements in PA in the intervention and control group. |
| Three good things: Promote work-life balance, reduce burnout, enhance reflection among newly licensed RNs | 3 good things - regarding joys, work, self-efficacy, self-care, relationships | 4-item Emotional Exhaustion (burnout) | Baseline  postintervention  6-months follow-up | | (1) Significant improvement in burnout between baseline and T1.  (2) No significant improvement at T2 sustained. |
| Reduction of Burnout in Mental Health Care Providers Using the Provider Resilience Mobile Application. | The Provider Resilience (PR) mobile app includes 2 assessment tools to help increase self-awareness of current levels of burnout: Professional Quality of Life Scale and the Burnout Visual Analog Scale. The app screen provides an overall graphic of the user's current resilience rating, customizable resilience builders and killers (encourages to be aware of factors that in-/decrease their resilience). Tools to help enhance resilience and reduce burnout, f.e. humorous cartoons, physical exercises, inspirational cards with motivational quotes), videos of consumers indication how their treatment impacted their lives, and video information on compassion fatigue. | Professional Quality of Life Scale (burnout) | Baseline  after 1 month | | (1) Significant decrease on the burnout subscale. |
| Well-being in Residency: Impact of an Online Physician Well-being Course on Resiliency and Burnout in Incoming Residents. | Physician Well-being Course (PWC): well-being (resilience, sleep, nutrition, mindfulness, exercise); resilience activities (gratitude, meditation, finding meaning) | Maslach Burnout Inventory | Pre- and postcourse | | (1) Significant improvement in burnout (EE, DP)  (2) Decline in the PA scale. |
| Decreasing Burnout and Improving Work Environment: The Impact of <i>Firgun</i> on a Pediatric Hematopoietic Cell Transplant Team. | Meaningful recognition: creating a culture of recurring altruistic complements related to job performance. | Maslach Burnout Inventory | Baseline  post completion (after 8 weeks) | | (1) No significant improvements in burnout measured with the ProQOL scale following the intervention.  (2) Significant improvements noted in EE after the intervention.  (3) No significant changes in DP or PA scores. |
| Forty-five good things: a prospective pilot study of the Three Good Things well-being intervention in the USA for healthcare worker emotional exhaustion, depression, work–life balance and happiness | 3 Good Things intervention | Maslach Burnout Inventory | Baseline  1 month, 6 months, 12 months | | (1) Significant reduction in EE from baseline to follow-up at 1 month, 6 months, 12 months. |
| A Mindfulness-Based Intervention for Acute Care Nursing Staff: A Pilot Study | Mindfulness-based: Headspace consisted of modules with different meditations, Insight Timer comprised guided meditation with topics like presence and grounding awareness. | The Copenhagen Burnout Inventory | Preintervention  at midpoint (after 15 days)  immediately postintervention (after 30 days) | | (1) Significant reductions in personal burnout over time. The reductions could be seen from baseline to both midpoint and follow-up.  (2) No statistically significant reduction in work-related burnout. |
| Feasibility, acceptability and preliminary efficacy of a mental health self-management app in clinicians working during the COVID-19 pandemic: A pilot randomised controlled trial | COVID Coach: self-management app comprising the following topics: Manage stress, Learn, Mood check, and find resources. These courses teach how to manage with mental wellbeing using relaxation and mindfulness exercises, deliver psychoeducational information and support by offering help links. | Copenhagen Burnout Inventory (CBI) | Pre- and postintervention | | (1) Non-significant improvement in burnout symptoms. |
| mHealth Gratitude Exercise Mindfulness App for Resiliency Among Neonatal Intensive Care Unit Staff: Three-Arm Pretest-Posttest Interventional Study. | mHealth intervention using a smartphone app: gratitude, exercise, and mindfulness smartphone app (GEM app). 3 evidence-based resilience interventions (a daily gratitude journal, regular exercise, or mindfulness meditation) were delivered, of which participants could choose one. | The Professional Quality of Life Scale | Pre- and postintervention | | (1) Overall, significant reduction in burnout was measured.  (2) The significant reduction in burnout was achieved in the gratitude intervention group. |
| Guided self-help mindfulness-based intervention for increasing psychological resilience and reducing job burnout in psychiatric nurses: A randomized controlled trial. | The self-help mindfulness intervention aimed to guide participants to focus on the present moment and to learn how to practice in a non-judgmental fashion during stressful experiences. The program included didactic instructions and practice with topics like Mindfulness, Concentration, and Awareness in Sports. It comprised principles of identifying habitual reaction patterns and cultivating non-judgmental awareness and acceptance of the present. The practice included body scan, mindful walking, breathing meditation, and transposition exercise. | Maslach Burnout Inventory | Baseline  after 4 and 8 weeks | | (1) Significant main effect of intervention, time, and a significant interaction between intervention and time.  (2) No changes in burnout scores over time in the control group. |
| Depression / anxiety | | | | | |
| Long-term beneficial effects of an online mind-body training program on stress and psychological outcomes in female healthcare providers: A non-randomized controlled study. | Mind-body training content and skills | Stress Response Inventory | Baseline  8 weeks (post-intervention)  12 weeks (1 month after completion of training) | | Depression measured as a subscale of stress response.  (1) MBT group with a significantly greater decrease in depression compared to the control group from T0 to T1 and T0 to T2 when analyzing time x group interaction. |
| Building Personal Resilience following an Online Resilience Training Program for BScN Students. | ORR = Online Resilience Resource (self-guided online training program which includes readings, videos, exercises, self-assessment tools) | Generalized Anxiety Disorder 7-item (GAD-7)  The Patient Health Questionnaire 9-item (PHQ-9) | Baseline  1 month post intervention  3 months post intervention | | (1) Non-significant decrease in scores of anxiety/depression.  (2) GAD-7 (anxiety): Non-significant reduction in anxiety symptoms.  (3) PHQ-9 (depression): Non-significant reduction in depressive symptoms. |
| Randomized controlled trial of the "WISER" intervention to reduce healthcare worker burnout. | 6 guided well-being modules | Center for Epidemiological Studies Depression Scale-10-item version (CES-D10) | Baseline  1 month post, 6 months post | | Depression = secondary outcome.  (1) No significant improvement in depression scores in cohort 1 compared to cohort 2.  (2) At 1 month, WISER was associated with significantly improved scores in depression in cohort 2.  (3) At 6 months, improvements in depression through WISER were significant in cohort 1.  (4) Combined cohort analyses: WISER significantly improved depression at 1 month and at 6 months. |
| Resilience Training for Work-Related Stress Among Health Care Workers: Results of a Randomized Clinical Trial Comparing In-Person and Smartphone-Delivered Interventions. | Mindfulness-based resilience training. Smartphone app: sleep, happiness and positivity, energy and focus, productivity; mindfulness and feeling less stressed. | Depression, Anxiety, and Stress Scale (DASS-21) | Baseline  6-weeks (after completion of intervention)  3-months post-intervention | | DEPRESSION:  (1) MBRT group: Non-significant decrease in depression.  (2) Smartphone group: Non-significant decrease in depression.  (3) Control group: Non-significant decrease in depression.  ANXIETY:  (1) MBRT group: Non-significant decrease in anxiety.  (2) Smartphone group: Non-significant decrease in anxiety.  (3) Control group: Non-significant decrease in anxiety. |
| Promoting resilience in healthcare workers during the COVID-19 pandemic with a brief online intervention. | Online resilience-enhancement course focused on mindfulness, mentalization and self-compassion. | Patient Health Questionnaire (PHQ-4) | Baseline  1 month and 2 months post-intervention | | EMOTIONAL DISTRESS:  (1) Significant decrease in emotional distress (PHQ-4) at 1 month and 2 months post-intervention.  (2) Significantly reduced emotional distress in those who completed the intervention compared to those who did not. |
| Effectiveness of a bite-sized web-based intervention to improve healthcare worker wellbeing: A randomized clinical trial of WISER. | WISER intervention (Web-based Implementation for the Science of Enhancing Resilience): a positive psychology program | Center for Epidemiological Studies Depression Scale-10-item version (CES-D10) | Baseline  postintervention (1 week, 1 month, 6 months, 12 months) | | Depressive symptoms:  (1) Significant reductions at all timepoints in the combined cohort analysis. |
| Impact of App-Delivered Mindfulness Meditation on Functional Connectivity, Mental Health, and Sleep Disturbances Among Physician Assistant Students: Randomized, Wait-list Controlled Pilot Study | Mobile app-delivered mindfulness meditation | The Depression Anxiety and Stress Scale | Baseline  >8 weeks | | (1) No significant improvements in depression or anxiety after completion of the intervention. |
| Effectiveness of self-help plus (SH+) in reducing anxiety and post-traumatic symptomatology among care home workers during the COVID-19 pandemic: a randomized controlled trial. | SH+: an individual self-help audio-visual tool: Doing what matters in times of stress. 5 core components: grounding, unhooking, acting on your values, being kind, making room. | Generalized Anxiety Disorder 7-item (GAD-7) | Baseline  week 7 (1 week after training completion)  week 20 (14 weeks after training completion) | | (1) No significant improvements in anxiety after completion of the intervention. |
| The Effects of an Online Mind-Body Training Program on Stress, Coping Strategies, Emotional Intelligence, Resilience and Psychological State | MBT on resilience, coping strategies, anger, stress, emotional intelligence, positive/negative affect. Movement-based meditation. | Stress Response Inventory (depression) | Baseline  4 weeks and 8 weeks following onset | | (1) Significant time x group interaction in depression. |
| Exploring the effects of an online asynchronous mindfulness meditation intervention with nursing students on stress, mood, and cognition: a descriptive study | Online mindfulness intervention: Mindfulness-based stress reduction (MBSR) model components | The Hospital Anxiety and Depression Scale (HADS) | Baseline  After 8 weeks, 24 weeks | | (1) Significant reduction in anxiety when training was performed weekly to daily.  (2) No significant reduction in depression following the intervention. |
| Forty-five good things: a prospective pilot study of the Three Good Things well-being intervention in the USA for healthcare worker emotional exhaustion, depression, work–life balance and happiness | 3 Good Things intervention | The Center for Epidemiological Studies Depression Scale-10 item | Baseline  After 1 month, 6 months, 12 months | | Depression:  (1) Significant improvement in depression symptoms at 1,6, 12 months |
| The Feasibility and Effectiveness of Online Guided Imagery Training for Health Professionals | Brief online guided imagery training | Patient Reported Outcomes Measurement Information System-Anxiety Scale | Pre- and postintervention | | Anxiety:  (1) Significant anxiety reduction following completion of the autogenic training module. |
| Effectiveness of an online positive psychology intervention among Tunisian healthcare students on mental health and study engagement during the Covid-19 pandemic | The CARE program: Coherence, Attention, Relationship, Engagement | The Depression, Anxiety and Stress Scales (DASS-21) | Baseline  immediately postintervention  3 months follow-up | | (1) Significant change in depression and anxiety between pre- and posttest.  (2) No significant differences in depression and anxiety between posttest and follow-up that indicates that achieved effects could be maintained.  (3) No significant changes over time could be seen in the control group. |
| Brief tele-mindfulness-based intervention: A multicenter randomized controlled trial. | Brief Mindfulness-based interventions (MBI) and progressive muscle relaxation (PMR). MBI: 7 mediation commands. PMR: tensions and relaxing specific muscle groups in sequence. | State-Trait Anxiety-20 Item Scale | Pre- and postintervention | | (1) Significant reduction of the state anxiety.  (2) No significant improvement in the trait anxiety. |
| Feasibility, acceptability and preliminary efficacy of a mental health self-management app in clinicians working during the COVID-19 pandemic: A pilot randomised controlled trial | COVID Coach: self-management app comprising the following topics: Manage stress, Learn, Mood check, and find resources. These courses teach how to manage with mental wellbeing using relaxation and mindfulness exercises, deliver psychoeducational information and support by offering help links. | The 10-item center for Epidemiological Studies Depression Scale, Spielberger State Trait Anxiety Inventory (STAI-S) | Pre- and postintervention | | (1) Significant reduction of anxiety from pre to postintervention. |
| Efficacy of the my health too online cognitive behavioral therapy program for healthcare workers during the COVID-19 pandemic: A randomized controlled trial. | Cognitive Behavioral therapy via the online MyHealthToo app. The app consisted of 7 sessions: (1) psychoeducation, (2) functional behavioral and cognitive coping strategies, (3) mindfulness, (4) acceptance, (5) promoting action toward values, (6) addressing barriers and motivation to use self-compassion as a psychological gift, (7) sleep problems and problem-solving strategies. | The Patient Health Questionnaire -2 items version (PHQ-2) | Pretreatment  mid-treatment (4 weeks), post-treatment (8 weeks)  1 month and 4 months follow-up | | (1) No significant changes in depression after the intervention. |
| Improving Resiliency in US Air Force Healthcare Personnel: A Randomized Preventive Trial | Stress Management and Resilience Training (SMART). Online Training with 4 modules | Generalized Anxiety Disorder Scale (GAD-7) | Baseline  at 12, 18, and 24 weeks | | (1) Significant reductions in anxiety at all timepoints.  (2) No significant differences between groups (In-person vs. Online) |
| Well-being | | | | | |
| Resilience Training for Work-Related Stress Among Health Care Workers: Results of a Randomized Clinical Trial Comparing In-Person and Smartphone-Delivered Interventions. | Mindfulness-based resilience training. Smartphone app: sleep, happiness and positivity, energy and focus, productivity; mindfulness and feeling less stressed. | WHO-5 Well-Being Index | Baseline  6-weeks (after completion of intervention)  3-months post-intervention | | (1) MBRT group: Non-significant improvement at 6 weeks, significant improvements made at 3 months.  (2) Smartphone group: Non-significant improvement at 6 weeks, significant improvements made at 3 months.  (3) Control group: Non-significant results. |
| Improving Healthcare Worker Resilience and Well-Being During COVID-19 Using a Self-Directed E-Learning Intervention. | Psychological well-being, self-care (Physical, Mind, Relationships, Emotions, Work), mindfulness and meditation | WHO-5 Well-Being Index | Pre- and postintervention | | (1) Significant increase in well-being between pre- and posttraining. |
| Effectiveness of self-help plus (SH+) in reducing anxiety and post-traumatic symptomatology among care home workers during the COVID-19 pandemic: a randomized controlled trial. | SH+: an individual self-help audio-visual tool: Doing what matters in times of stress. 5 core components: grounding, unhooking, acting on your values, being kind, making room. | WHO-5 Well-Being Index | Baseline  week 7 (1 week after training completion)  week 20 (14 weeks after training completion) | | (1) No significant improvement in well-being at post-intervention or at follow-up. |
| Decreasing Burnout and Improving Work Environment: The Impact of <i>Firgun</i> on a Pediatric Hematopoietic Cell Transplant Team. | Meaningful recognition: creating a culture of recurring altruistic complements related to job performance. | WHO-5 Well-being Index | Baseline  post completion (after 8 weeks) | | (1) No significant improvements in total well-being scores found. |
| Effectiveness of an online positive psychology intervention among Tunisian healthcare students on mental health and study engagement during the Covid-19 pandemic | The CARE program: Coherence, Attention, Relationship, Engagement | The Warwick-Edinburgh Mental Well-being Scale (WEMWBS) | Baseline  immediately postintervention  3 months follow-up | | (1) Significant improvement in well-being between baseline and follow-up.  (2) No changes are seen between posttest and follow-up suggesting that effects were stable over time.  (3) No significant changes over time could be seen in the control group. |
| Brief tele-mindfulness-based intervention: A multicenter randomized controlled trial. | Brief Mindfulness-based interventions (MBI) and progressive muscle relaxation (PMR). MBI: 7 mediation commands. PMR: tensions and relaxing specific muscle groups in sequence. | WHO-5 Well-Being Index | Pre- and postintervention | | (1) Significant improvement in well-being.  (2) The MBI group achieved significantly bigger improvements in well-being than the PMR group. |
| Feasibility, acceptability and preliminary efficacy of a mental health self-management app in clinicians working during the COVID-19 pandemic: A pilot randomised controlled trial | COVID Coach: self-management app comprising the following topics: Manage stress, Learn, Mood check, and find resources. These courses teach how to manage with mental wellbeing using relaxation and mindfulness exercises, deliver psychoeducational information and support by offering help links. | The World Health Organization Five Well-Being Index (WHO-5) | Pre- and postintervention | | (1) Non-significant improvement in well-being after completion of the intervention. |

## Blended group:

Table 11 detailed outcomes of the blended group

| Title | Intervention | Measurement tool | Timepoint of assessment | Results |
| --- | --- | --- | --- | --- |
| Resilience | | | | |
| Decreasing Stress and Burnout in Nurses: Efficacy of Blended Learning With Stress Management and Resilience Training Program. | SMART: Stress Management and Resiliency Training | Connor-Davidson Resilience Scale (CD-RISC) | Baseline (week 0)  week 8, 12, 24 | (1) Non-significant improvements in resilience at week 8 and 12.  (2) Significant improvement in resilience at week 24. |
| Impact of a Blended Web-Based Mindfulness Programme for General Practitioners: a Pilot Study | Mindfulness intervention based on the standard program by Kabat-Zinn | Connor-Davidson Resilience Scale (CD-RISC) | Pre- & postintervention | (1) Non-significant improvement in resilience when practicing once per week.  (2) Non-significant improvement in resilience when practicing at least twice per week. |
| Implementation of a Web-Based Resilience Enhancement Training for Nurses: Pilot Randomized Controlled Trial. | The REsOluTioN Program: Resilience Enhancement Online Training for Nurses | Brief Resilience Scale (BRS) | Baseline  after 6 weeks | (1) Positive trend toward improvement in resilience, however no significant differences between groups and time. |
| The impact of Stress Management and Resailience Training (SMART) on academic physicians during the implementation of a new Health Information System: An exploratory randomized controlled trial. | SMART: The Stress Management and Resilience Training program. Principles: gratitude, acceptance, compassion, higher meaning, forgiveness | Connor-Davidson Resilience Scale (CD-RISC) | Baseline  3 months follow-up, 6 months follow-up | (1) Non-significant improvements found in resilience at 3 months or 6 months follow-up. |
| Mind-Body Skills Training for Resident Wellness: A Pilot Study of a Brief Mindfulness Intervention. | MBST: Mind-Body Skills Training for Resilience, Effectiveness, and Mindfulness | Smith's Brief Resilience Scale | Baseline (T1)  postintervention (T2)  6 months follow-up (t3) | (1) Significant improvement in resilience between T1 and T2.  (2) Non-significant improvement in resilience between T1 and T3. |
| Sustained resiliency building and burnout reduction for healthcare professionals via organizational sponsored mindfulness programming. | Mindfulness Based Intervention (MBI): Mindfulness in Motion (MIM) | Connor-Davidson Resilience Scale (CD-RISC) | Pre- and postintervention  follow-up: 3 groups = 0-6M, 7-12M, 13-28M (mean: 12.2M) | (1) Significant improvement in resilience from baseline to follow-up.  (2) Difference in resilience between posttest and follow-up was non-significant.  (3) Participants that were surveyed at 0-6 months and those at 7-12 sustained a statistically significant increase in resilience between pretest and follow-up. |
| The Community Resiliency Model¬Æ to promote nurse well-being. | Community Resiliency Model training (CRM): sensory awareness techniques to improve emotional balance | The Connor-Davidson Resilience Scale-10 (CD-RISC) | Baseline  week 1, 3 months, 1 year | (1) Significant improvement in resilience over time.  (2) Group-by-time not significant. |
| Effects of the Brief Simha Kriya Breathing Practice for Health Care Workers During the COVID-19 Pandemic. | A brief pranayama yoga practice: breathwork practice. Simha Kriya yoga: forced exhalation with tongue sticking out, 21x at a normal pace; forced exhalation with tongue rolled upward, 21x, at a normal pace; breath retention for 30-60s; followed by a 2min meditation. Weekly support sessions via zoom. | Brief Resilient Coping Scale (BRCS) | Baseline  week 1, week 4 | (1) Significant improvement in resilience scores at 4-week follow-up. |
| A comparative study of well-being, resilience, mindfulness, negative emotions, stress, and burnout among nurses after an online mind-body based intervention during the first COVID-19 pandemic crisis | Mind-body based intervention that comprises 36 mind-body based micro practices. Nurse leaders provided support by sharing the exercises 3x per week in a group setting. Videos and audio files that teach techniques including relaxation response, mindfulness-based stress reduction, single-focus meditation, self-regulation exercises, breathing practices, awareness practices, spiritually and reframing strategies based of existential positive psychology, and journaling, were offered. | Brief Resilience Scale (BRS) | Baseline  follow-up at 6 months postintervention | (1) No significant improvement in resilience. |
| A compassion-based program to reduce psychological distress in medical students: A pilot randomized clinical trial | Compassion Cultivation Training (CCT): meditation program that aims to cultivate compassion and empathy to reduce psychological distress and promote well-being. The program includes the following 6 steps: (1) learning to focus and settle the mind; (2) experiencing compassion and loving-kindness for a loved one; (3) experiencing compassion and loving-kindness for oneself; (4) experiencing compassion towards others, premised in common humanity and interconnectedness; (5) experiencing compassion towards all beings; and (6) “active compassion” practice. | Brief Resilience Scale (BRS) | Pre-and postintervention  2-months follow-up | (1) No significant difference in resilience after the intervention compared to the waitlist control. |
| Effectiveness of an online mental health strengthening module to build resilience and overcome stress for transitional aged medical students | The Transition and Adaptation towards Resiliency module consists of 4 submodules: (1) Changes in life to become more independent and adapting to a new environment; (2) stress and ways to overcome stress; (3) mental health problems and symptoms of mental disorders; (4) mental health help-seeking | The Connor-Davidson Resilience Scale (CD-RISC) | Baseline  after 4, 8, 12 weeks | (1) No significant differences compared to the control group after completion of the intervention.  (2) Significant changes in resilience over time. |
| Feasibility and acceptability of a culturally adapted psychological first aid training intervention (Preparing Me) to support the mental health and well-being of front-line healthcare workers in China: a feasibility randomized controlled trial | Preparing me is a psychological first aid training intervention. READ-Y PFA (psychological first aid) training program consisted of elements of trauma recovery principles, specific techniques and case scenarios. READ-Y PFA: Rapport, Evaluation, Aid, Disposition, Care for Yourself and others. | Brief Resilience Scale (BRS) | Pre- and postintervention  3 months follow-up | (1) Statistically significant improvements in resilience over time. |
| Efficacy of a Text-Based Mental Health Coaching App in Improving the Symptoms of Stress, Anxiety, and Depression: Randomized Controlled Trial | ThroughFullChat: One-on-one, asynchronous, text-based coaching and self-guided tools | Brief Resilience Scale (BRS) | Baseline  after the 3 months intervention period | (1) No significant changes in resilience scores after the intervention. |
| Stress | | | | |
| Decreasing Stress and Burnout in Nurses: Efficacy of Blended Learning With Stress Management and Resilience Training Program. | SMART: Stress Management and Resiliency Training | Perceived Stress Scale (PSS) | Baseline (week 0)  week 8, 12, 24 | (1) Significant reduction in stress at week 24. |
| The impact of Stress Management and Resailience Training (SMART) on academic physicians during the implementation of a new Health Information System: An exploratory randomized controlled trial. | SMART: The Stress Management and Resilience Training program. Principles: gratitude, acceptance, compassion, higher meaning, forgiveness | 10-item Perceived Stress Scale (PSS) | Baseline  3 months follow-up, 6 months follow-up | (1) Non-significant improvements found in stress at 3 months or 6 months follow-up. |
| Mind-Body Skills Training for Resident Wellness: A Pilot Study of a Brief Mindfulness Intervention. | MBST: Mind-Body Skills Training for Resilience, Effectiveness, and Mindfulness | Cohen's Perceived Stress | Baseline (T1)  postintervention (T2)  6 months follow-up (t3) | (1) Significant improvement in perceived stress between T1 and T2.  (2) No significant change in stress between T1 and T3. |
| Interventions to reduce burnout and improve resilience: Impact on a health system's outcomes | CRM: Crew Resource Management  MIM: Mindfulness in Motion training, Gabbe health and wellness program, Mind-body skills | Perceived Stress Scale (PSS) | Baseline (1 week prior to intervention)  8 weeks, 9 months | (1) Non-significant improvements in stress after completion of at least 1 intervention.  (2) No significant improvements in stress when no intervention is done. |
| Sustained resiliency building and burnout reduction for healthcare professionals via organizational sponsored mindfulness programming. | Mindfulness Based Intervention (MBI): Mindfulness in Motion (MIM) | Perceived Stress Scale (PSS) | Pre- & postintervention  follow-up: 3 groups = 0-6M, 7-12M, 13-28M (mean: 12.2M) | (1) Significant improvement in perceived stress from baseline to follow-up.  (2) No significant difference in stress between posttest and follow-up.  (3) The only group that could sustain significant results from pretest to follow-up was the one surveyed at 13-28 months. |
| Brief video-module administered mindfulness program for physicians: a pilot study | Mindfulness training, body scan, guided meditations | Perceived Stress Scale (PSS) | Baseline  end-of-program, 8 weeks post-intervention | (1) Statistically significant decrease in stress between baseline and follow-up at 8 weeks. |
| Training on mind-body skills: Feasibility and effects on physician mindfulness, compassion, and associated effects on stress, burnout, and clinical outcomes | Online Mind-Body Skills Training and interactive discussion sessions | 10-item Perceived Stress Scale (PSS) | Pre- and postintervention | (1) No significant improvements in perceived stress following the intervention. |
| Effects of the Brief Simha Kriya Breathing Practice for Health Care Workers During the COVID-19 Pandemic. | A brief pranayama yoga practice: breathwork practice. Simha Kriya yoga: forced exhalation with tongue sticking out, 21x at a normal pace; forced exhalation with tongue rolled upward, 21x, at a normal pace; breath retention for 30-60s; followed by a 2min meditation. Weekly support sessions via zoom. | Perceived Stress Scale (PSS) | Baseline  week 1, week 4 | (1) No significant changes in perceived stress. |
| A comparative study of well-being, resilience, mindfulness, negative emotions, stress, and burnout among nurses after an online mind-body based intervention during the first COVID-19 pandemic crisis | Mind-body based intervention that comprises 36 mind-body based micro practices. Nurse leaders provided support by sharing the exercises 3x per week in a group setting. Videos and audio files that teach techniques including relaxation response, mindfulness-based stress reduction, single-focus meditation, self-regulation exercises, breathing practices, awareness practices, spiritually and reframing strategies based of existential positive psychology, and journaling, were offered. | Perceived Stress Scale (PSS) | Baseline  follow-up at 6 months postintervention | (1) Significant reduction in stress. |
| A compassion-based program to reduce psychological distress in medical students: A pilot randomized clinical trial | Compassion Cultivation Training (CCT): meditation program that aims to cultivate compassion and empathy to reduce psychological distress and promote well-being. The program includes the following 6 steps: (1) learning to focus and settle the mind; (2) experiencing compassion and loving-kindness for a loved one; (3) experiencing compassion and loving-kindness for oneself; (4) experiencing compassion towards others, premised in common humanity and interconnectedness; (5) experiencing compassion towards all beings; and (6) “active compassion” practice. | Depression Anxiety Stress Scales (DASS-21) | Pre- and post-intervention  2-months follow-up | (1) Significant reduction in stress after the intervention.  (2) No significant results in the waitlist control. |
| Effectiveness of an online mental health strengthening module to build resilience and overcome stress for transitional aged medical students | The Transition and Adaptation towards Resiliency module consists of 4 submodules: (1) Changes in life to become more independent and adapting to a new environment; (2) stress and ways to overcome stress; (3) mental health problems and symptoms of mental disorders; (4) mental health help-seeking | Perceived Stress Scale (PSS) | Baseline  after 4, 8, 12 weeks | (1) Significant difference in stress after completion of the intervention compared to the control group.  (2) Significant changes in stress over time. |
| Feasibility and acceptability of a culturally adapted psychological first aid training intervention (Preparing Me) to support the mental health and well-being of front-line healthcare workers in China: a feasibility randomized controlled trial | Preparing me is a psychological first aid training intervention. READ-Y PFA (psychological first aid) training program consisted of elements of trauma recovery principles, specific techniques and case scenarios. READ-Y PFA: Rapport, Evaluation, Aid, Disposition, Care for Yourself and others. | 21-Item Depression, Anxiety, and Stress Scale (DASS-21) | Pre- and postintervention  3 months follow-up | (1) Statistically significant reduction in stress over time. |
| Efficacy of a Text-Based Mental Health Coaching App in Improving the Symptoms of Stress, Anxiety, and Depression: Randomized Controlled Trial | ThroughFullChat: One-on-one, asynchronous, text-based coaching and self-guided tools | Depression, Anxiety, and Stress Scale-21 (DASS) | Baseline  after the 3 months intervention period | (1) Significant reduction in stress in the intervention group among corporate staff. |
| Burnout | | | | |
| Decreasing Stress and Burnout in Nurses: Efficacy of Blended Learning With Stress Management and Resilience Training Program. | SMART: Stress Management and Resiliency Training | Copenhagen Burnout Inventory (CBI) | Baseline (week 0)  week 8, 12, 24 | (1) Personal Burnout: Significant reduction at week 8,12, 24.  (2) Work-related burnout: Significant reduction at week 8, 12, 24.  (3) Client related burnout: Significant decrease at week 12 and 24 but not at week 8. |
| Impact of a Blended Web-Based Mindfulness Programme for General Practitioners: a Pilot Study | Mindfulness intervention based on the standard program by Kabat-Zinn | Burnout Clinical Subtype Questionnaire (BCSQ-12) | Pre- and postintervention | (1) Non-significant improvement in resilience when practicing once per week.  (2) Non-significant improvement in resilience when practicing at least twice per week. |
| Mind-Body Skills Training for Resident Wellness: A Pilot Study of a Brief Mindfulness Intervention. | MBST: Mind-Body Skills Training for Resilience, Effectiveness, and Mindfulness | Maslach Burnout Inventory (MBI) | Baseline (T1)  postintervention (T2)  6 months follow-up (t3) | (1) Non-significant improvement in EE between T1 & T2 and T1 and T3.  (2) Non-significant improvement in DP between T1 and T2. Significant improvement in DP between T1 and T3.  (3) Significant improvement in PA between T1 and T2, but not significant between T1 and T3. |
| Interventions to reduce burnout and improve resilience: Impact on a health system's outcomes | CRM: Crew Resource Management  MIM: Mindfulness in Motion training, Gabbe health and wellness program, Mind-body skills | Emotional Exhaustion, Depersonalization | Baseline (1 week prior to intervention)  8 weeks, 9 months | EE:  (1) Significant improvement in EE from pre- to postintervention when at least 1 intervention is done.  (2) No significant difference in EE between pre- and posttest when no intervention is completed. DP:  (3) Significant improvement in DP when at least 1 program is performed.  (4) No significant difference between pre and posttest in those who did not complete any intervention. |
| Sustained resiliency building and burnout reduction for healthcare professionals via organizational sponsored mindfulness programming. | Mindfulness Based Intervention (MBI): Mindfulness in Motion (MIM) | Maslach burnout inventory (MBI) | Pre- and postintervention  follow-up: 3 groups = 0-6M, 7-12M, 13-28M (mean: 12.2M) | (1) Significant improvement in burnout from baseline to follow-up in those who initially met burnout criteria.  (2) No statistically significant change from posttest to follow-up.  (3) The group that was surveyed at 0-6 months was the only group that sustained a significant decrease in burnout from pretest to follow-up. |
| The Community Resiliency Model¬Æ to promote nurse well-being. | Community Resiliency Model training (CRM): sensory awareness techniques to improve emotional balance | The Copenhagen Burnout Inventory (CBI) | Baseline  week 1, 3 months, 1 year | (1) No significant improvement in burnout over time.  (2) Group-by-time not significant. |
| Brief video-module administered mindfulness program for physicians: a pilot study | Mindfulness training, body scan, guided meditations | Maslach Burnout Inventory | Baseline  end-of-program, 8 weeks post-intervention | (1) Statistically significant improvement in personal accomplishment (PA) between baseline and follow-up at 8 weeks.  (2) Non-significant decrease in emotional exhaustion (EE) and depersonalization (DP) between baseline and follow-up at 8 weeks. |
| Training on mind-body skills: Feasibility and effects on physician mindfulness, compassion, and associated effects on stress, burnout, and clinical outcomes | Online Mind-Body Skills Training and interactive discussion sessions | Emotional Exhaustion, Depersonalization | Pre- and postintervention | (1) Significant mean difference in burnout (EE, DP) after participating.  (2) No significant changes in participants who did 0 hours of practice. |
| A comparative study of well-being, resilience, mindfulness, negative emotions, stress, and burnout among nurses after an online mind-body based intervention during the first COVID-19 pandemic crisis | Mind-body based intervention that comprises 36 mind-body based micro practices. Nurse leaders provided support by sharing the exercises 3x per week in a group setting. Videos and audio files that teach techniques including relaxation response, mindfulness-based stress reduction, single-focus meditation, self-regulation exercises, breathing practices, awareness practices, spiritually and reframing strategies based of existential positive psychology, and journaling, were offered. | The Burnout Clinical Subtypes Questionnaire (BCSQ-12) | Baseline  follow-up at 6 months postintervention | (1) No significant improvement in burnout scores. |
| A compassion-based program to reduce psychological distress in medical students: A pilot randomized clinical trial | Compassion Cultivation Training (CCT): meditation program that aims to cultivate compassion and empathy to reduce psychological distress and promote well-being. The program includes the following 6 steps: (1) learning to focus and settle the mind; (2) experiencing compassion and loving-kindness for a loved one; (3) experiencing compassion and loving-kindness for oneself; (4) experiencing compassion towards others, premised in common humanity and interconnectedness; (5) experiencing compassion towards all beings; and (6) “active compassion” practice. | Maslach Burnout Inventory | Pre- and postintervention  2-months follow-up | (1) Significant reduction in burnout scores (EE) following the intervention and at follow-up.  (2) No significant results in the waitlist control. |
| Feasibility and acceptability of a culturally adapted psychological first aid training intervention (Preparing Me) to support the mental health and well-being of front-line healthcare workers in China: a feasibility randomized controlled trial | Preparing me is a psychological first aid training intervention. READ-Y PFA (psychological first aid) training program consisted of elements of trauma recovery principles, specific techniques and case scenarios. READ-Y PFA: Rapport, Evaluation, Aid, Disposition, Care for Yourself and others. | Professional Quality of Life Scale (ProQOL) | Pre- and postintervention  3 months follow-up | (1) Statistically significant reduction in burnout over time. |
| Depresison, anxiety | | | | |
| Decreasing Stress and Burnout in Nurses: Efficacy of Blended Learning With Stress Management and Resilience Training Program. | SMART: Stress Management and Resiliency Training | Generalized Anxiety Scale | Baseline (week 0)  week 8, 12, 24 | (1) Significant decrease in anxiety at week 8, 12,24. |
| The impact of Stress Management and Resailience Training (SMART) on academic physicians during the implementation of a new Health Information System: An exploratory randomized controlled trial. | SMART: The Stress Management and Resilience Training program. Principles: gratitude, acceptance, compassion, higher meaning, forgiveness | Generalized Anxiety Disorder (GAD7) | Baseline  3 months follow-up, 6 months follow-up | (1) Non-significant improvements found in anxiety at 3 months or 6 months follow-up. |
| A compassion-based program to reduce psychological distress in medical students: A pilot randomized clinical trial | Compassion Cultivation Training (CCT): meditation program that aims to cultivate compassion and empathy to reduce psychological distress and promote well-being. The program includes the following 6 steps: (1) learning to focus and settle the mind; (2) experiencing compassion and loving-kindness for a loved one; (3) experiencing compassion and loving-kindness for oneself; (4) experiencing compassion towards others, premised in common humanity and interconnectedness; (5) experiencing compassion towards all beings; and (6) “active compassion” practice. | Depression Anxiety Stress Scales (DASS-21) | Pre-and post-intervention  2-months follow-up | (1) Significant reductions in depression and anxiety following the intervention and could be remained in anxiety at follow-up.  (2) No significant results in the waitlist control. |
| Effectiveness of an online mental health strengthening module to build resilience and overcome stress for transitional aged medical students | The Transition and Adaptation towards Resiliency module consists of 4 submodules: (1) Changes in life to become more independent and adapting to a new environment; (2) stress and ways to overcome stress; (3) mental health problems and symptoms of mental disorders; (4) mental health help-seeking | Depression Anxiety Scale (DASS) | Baseline  after 4, 8, 12 weeks | (1) No significant differences compared to the control group after completion of the intervention in depression and anxiety.  (2) Significant changes in depression and anxiety over time. |
| Feasibility and acceptability of a culturally adapted psychological first aid training intervention (Preparing Me) to support the mental health and well-being of front-line healthcare workers in China: a feasibility randomized controlled trial | Preparing me is a psychological first aid training intervention. READ-Y PFA (psychological first aid) training program consisted of elements of trauma recovery principles, specific techniques and case scenarios. READ-Y PFA: Rapport, Evaluation, Aid, Disposition, Care for Yourself and others. | 21-Item Depression, Anxiety, and Stress Scale (DASS-21) | Pre- and postintervention  3 months follow-up | (1) Statistically significant reduction in depression and anxiety over time. |
| Efficacy of a Text-Based Mental Health Coaching App in Improving the Symptoms of Stress, Anxiety, and Depression: Randomized Controlled Trial | ThroughFullChat: One-on-one, asynchronous, text-based coaching and self-guided tools | Depression, Anxiety, and Stress Scale-21 | Baseline  after the 3 months intervention period | (1) Significant reduction in depression and anxiety in the intervention group.  (2) Significant improvements in depression and anxiety in females compared with males. |
| Well-being | | | | |
| Implementation of a Web-Based Resilience Enhancement Training for Nurses: Pilot Randomized Controlled Trial. | The REsOluTioN Program: Resilience Enhancement Online Training for Nurses | Warwick-Edinburgh Mental Wellbeing Scale | Baseline  after 6 weeks | (1) Positive trend toward improvement in well-being, however no significant differences between groups and time. |
| Interventions to reduce burnout and improve resilience: Impact on a health system's outcomes | CRM: Crew Resource Management  MIM: Mindfulness in Motion training, Gabbe health and wellness program, Mind-body skills | PWI (physician well-being index) | Baseline (1 week prior to intervention)  8 weeks, 9 months | (1) Significant improvement in well-being from pre- to postintervention when at least 1 training is performed.  (2) No significant improvement in well-being when no intervention is completed. |
| The Community Resiliency Model¬Æ to promote nurse well-being. | Community Resiliency Model training (CRM): sensory awareness techniques to improve emotional balance | WHO-5 Well-being Index (WHO-5) | Baseline  week 1, 3 months, 1 year | (1) Significant improvement in well-being over time.  (2) Group-by-time not significant. |
| Training on mind-body skills: Feasibility and effects on physician mindfulness, compassion, and associated effects on stress, burnout, and clinical outcomes | Online Mind-Body Skills Training and interactive discussion sessions | The Physician Well-Being Index (PWBI) | Pre- and postintervention | (1) Significant mean difference in well-being in participants who completed at least 1 hour of training.  (2) No significant changes in participants who did 0 hours of practice. |
| A comparative study of well-being, resilience, mindfulness, negative emotions, stress, and burnout among nurses after an online mind-body based intervention during the first COVID-19 pandemic crisis | Mind-body based intervention that comprises 36 mind-body based micro practices. Nurse leaders provided support by sharing the exercises 3x per week in a group setting. Videos and audio files that teach techniques including relaxation response, mindfulness-based stress reduction, single-focus meditation, self-regulation exercises, breathing practices, awareness practices, spiritually and reframing strategies based of existential positive psychology, and journaling, were offered. | PERMA Profiler | Baseline  follow-up at 6 months postintervention | (1) No significant improvement in well-being was observed. |
| A compassion-based program to reduce psychological distress in medical students: A pilot randomized clinical trial | Compassion Cultivation Training (CCT): meditation program that aims to cultivate compassion and empathy to reduce psychological distress and promote well-being. The program includes the following 6 steps: (1) learning to focus and settle the mind; (2) experiencing compassion and loving-kindness for a loved one; (3) experiencing compassion and loving-kindness for oneself; (4) experiencing compassion towards others, premised in common humanity and interconnectedness; (5) experiencing compassion towards all beings; and (6) “active compassion” practice. | Pemberton Happiness Index (PHI) -11 items | Pre-and post-intervention  2-months follow-up | (1) No significant difference in well-being after the intervention compared to the waitlist control. |

# Effective Public Health Practice Project (EPHPP) Risk of bias assessment

| Title | Selection Bias | Study Design | Confounders | Blinding | Data Collection  Method | Withdrawals and  Dropouts | Global rating |
| --- | --- | --- | --- | --- | --- | --- | --- |
| Online | | | | | | | |
| Long-term beneficial effects of an online mind-body training program on stress and psychological outcomes in female healthcare providers: A non-randomized controlled study. | Strong | Moderate | Strong | Weak | Strong | Strong | Moderate |
| Acute Effects of Online Mind-Body Skills Training on Resilience, Mindfulness, and Empathy | Weak | Moderate | Weak | Weak | Strong | Weak | Weak |
| Building Personal Resilience following an Online Resilience Training Program for BScN Students. | Strong | Weak | Strong | Weak | Moderate | Weak | Weak |
| Randomized controlled trial of the "WISER" intervention to reduce healthcare worker burnout. | Weak | Strong | Strong | Weak | Strong | Weak | Weak |
| Resilience Training for Work-Related Stress Among Health Care Workers: Results of a Randomized Clinical Trial Comparing In-Person and Smartphone-Delivered Interventions. | Strong | Strong | Strong | Weak | Strong | Moderate | Moderate |
| Promoting resilience in healthcare workers during the COVID-19 pandemic with a brief online intervention. | Weak | Strong | Strong | Weak | Strong | Weak | Weak |
| Brief Online Focused Attention Meditation Training: Immediate Impact. | Moderate | Moderate | Weak | Weak | Strong | Strong | Weak |
| What Is the Impact of Online Training in Mind-Body Skills? | Weak | Moderate | Strong | Weak | Strong | Weak | Weak |
| Effectiveness of a bite-sized web-based intervention to improve healthcare worker wellbeing: A randomized clinical trial of WISER. | Weak | Strong | Strong | Weak | Strong | Moderate | Weak |
| Online Training in Mind-Body Therapies: Different Doses, Long-term Outcomes | Strong | Moderate | Weak | Weak | Strong | Strong | Weak |
| Impact of App-Delivered Mindfulness Meditation on Functional Connectivity, Mental Health, and Sleep Disturbances Among Physician Assistant Students: Randomized, Wait-list Controlled Pilot Study | Strong | Strong | Strong | Moderate | Strong | Strong | Strong |
| Smartphone-based home workout program for shift-work nurses working during the COVID-19 pandemic. | Strong | Moderate | Strong | Weak | Strong | Strong | Moderate |
| Improving Healthcare Worker Resilience and Well-Being During COVID-19 Using a Self-Directed E-Learning Intervention. | Weak | Moderate | Strong | Weak | Strong | Moderate | Weak |
| Effectiveness of self-help plus (SH+) in reducing anxiety and post-traumatic symptomatology among care home workers during the COVID-19 pandemic: a randomized controlled trial. | Moderate | Strong | Weak | Moderate | Strong | Moderate | Moderate |
| A Smartphone App to Reduce Burnout in the Emergency Department: A Pilot Randomized Controlled Trial. | Strong | Strong | Weak | Weak | Strong | Weak | Weak |
| Evaluation of a Web-Based Holistic Stress Reduction Pilot Program Among Nurse-Midwives | Moderate | Moderate | Weak | Weak | Weak | Moderate | Weak |
| Help in hand after traumatic events: a randomized controlled trial in health care professionals on the efficacy, usability, and user satisfaction of a self-help app to reduce trauma-related symptoms | Weak | Strong | Strong | Weak | Strong | Moderate | Weak |
| The Nurse Empowerment Program for Nurses in Direct Care Positions | Weak | Moderate | Weak | Weak | Strong | Strong | Weak |
| Can stoic training develop medical student empathy and resilience? A mixed-methods study. | Strong | Moderate | Weak | Weak | Strong | Strong | Weak |
| Three good things: Promote work-life balance, reduce burnout, enhance reflection among newly licensed RNs | Moderate | Moderate | Weak | Weak | Strong | Weak | Weak |
| Reduction of Burnout in Mental Health Care Providers Using the Provider Resilience Mobile Application. | Strong | Moderate | Weak | Weak | Strong | Strong | Weak |
| Well-being in Residency: Impact of an Online Physician Well-being Course on Resiliency and Burnout in Incoming Residents. | Moderate | Moderate | Weak | Weak | Strong | Strong | Weak |
| Decreasing Burnout and Improving Work Environment: The Impact of <i>Firgun</i> on a Pediatric Hematopoietic Cell Transplant Team. | Weak | Moderate | Weak | Weak | Strong | Moderate | Weak |
| The Effects of an Online Mind-Body Training Program on Stress, Coping Strategies, Emotional Intelligence, Resilience and Psychological State | Weak | Strong | Strong | Weak | Strong | Weak | Weak |
| Exploring the effects of an online asynchronous mindfulness meditation intervention with nursing students on stress, mood, and cognition: a descriptive study | Strong | Moderate | Weak | Weak | Strong | Strong | Weak |
| Building personal resilience in paramedic students | Weak | Strong | Weak | Weak | Strong | Strong | Weak |
| Building personal resilience in primary care paramedic students, and subsequent skill decay | Weak | Moderate | Weak | Weak | Strong | Weak | Weak |
| Forty-five good things: a prospective pilot study of the Three Good Things well-being intervention in the USA for healthcare worker emotional exhaustion, depression, work–life balance and happiness | Weak | Moderate | Weak | Weak | Strong | Weak | Weak |
| The Feasibility and Effectiveness of Online Guided Imagery Training for Health Professionals | Strong | Moderate | Weak | Weak | Strong | Strong | Weak |
| Effectiveness of an online positive psychology intervention among Tunisian healthcare students on mental health and study engagement during the Covid-19 pandemic | Strong | Strong | Strong | Weak | Strong | Strong | Moderate |
| Leadership Link: Evaluation of an Online Leadership Curriculum for Certified Midwives and Certified Nurse-Midwives | Moderate | Moderate | Weak | Weak | Strong | Strong | Weak |
| Brief tele-mindfulness-based intervention: A multicenter randomized controlled trial. | Weak | Strong | Strong | Strong | Strong | Strong | Moderate |
| A Mindfulness-Based Intervention for Acute Care Nursing Staff: A Pilot Study | Strong | Moderate | Weak | Weak | Strong | Weak | Weak |
| Impact of an online training tool on individual and organizational resilience and mindfulness among radiological personnel in Norway | Weak | Moderate | Weak | Weak | Strong | Weak | Weak |
| Feasibility, acceptability and preliminary efficacy of a mental health self-management app in clinicians working during the COVID-19 pandemic: A pilot randomised controlled trial | Strong | Strong | Strong | Weak | Strong | Strong | Moderate |
| mHealth Gratitude Exercise Mindfulness App for Resiliency Among Neonatal Intensive Care Unit Staff: Three-Arm Pretest-Posttest Interventional Study. | Weak | Moderate | Strong | Weak | Strong | Moderate | Weak |
| Efficacy of the my health too online cognitive behavioral therapy program for healthcare workers during the COVID-19 pandemic: A randomized controlled trial. | Strong | Strong | Strong | Moderate | Strong | Strong | Strong |
| Improving Resiliency in US Air Force Healthcare Personnel: A Randomized Preventive Trial | Strong | Strong | Strong | Weak | Strong | Strong | Moderate |
| Guided self-help mindfulness-based intervention for increasing psychological resilience and reducing job burnout in psychiatric nurses: A randomized controlled trial. | Strong | Strong | Strong | Strong | Strong | Strong | Strong |
| Blended | | | | | | | |
| Decreasing Stress and Burnout in Nurses: Efficacy of Blended Learning With Stress Management and Resilience Training Program. | Weak | Moderate | Weak | Weak | Strong | Weak | **Weak** |
| Impact of a Blended Web-Based Mindfulness Programme for General Practitioners: a Pilot Study | Weak | Moderate | Weak | Weak | Strong | Strong | **Weak** |
| Implementation of a Web-Based Resilience Enhancement Training for Nurses: Pilot Randomized Controlled Trial. | Strong | Strong | Strong | Moderate | Strong | Weak | **Moderate** |
| The impact of Stress Management and Resailience Training (SMART) on academic physicians during the implementation of a new Health Information System: An exploratory randomized controlled trial. | Weak | Strong | Strong | Weak | Strong | Strong | **Weak** |
| Mind-Body Skills Training for Resident Wellness: A Pilot Study of a Brief Mindfulness Intervention. | Weak | Moderate | Weak | Weak | Strong | Strong | **Weak** |
| Interventions to reduce burnout and improve resilience: Impact on a health system's outcomes | Moderate | Moderate | Weak | Weak | Strong | Moderate | **Weak** |
| Sustained resiliency building and burnout reduction for healthcare professionals via organizational sponsored mindfulness programming. | Weak | Moderate | Weak | Weak | Strong | Strong | **Weak** |
| The Community Resiliency Model¬Æ to promote nurse well-being. | Weak | Strong | Strong | Weak | Strong | Weak | **Weak** |
| Brief video-module administered mindfulness program for physicians: a pilot study | Weak | Moderate | Weak | Weak | Strong | Strong | **Weak** |
| Training on mind-body skills: Feasibility and effects on physician mindfulness, compassion, and associated effects on stress, burnout, and clinical outcomes | Moderate | Moderate | Weak | Weak | Strong | Moderate | **Weak** |
| Effects of the Brief Simha Kriya Breathing Practice for Health Care Workers During the COVID-19 Pandemic. | Weak | Moderate | Weak | Weak | Strong | Moderate | **Weak** |
| A comparative study of well-being, resilience, mindfulness, negative emotions, stress, and burnout among nurses after an online mind-body based intervention during the first COVID-19 pandemic crisis | Strong | Moderate | Strong | Weak | Strong | Weak | **Weak** |
| A compassion-based program to reduce psychological distress in medical students: A pilot randomized clinical trial | Strong | Strong | Weak | Moderate | Strong | Strong | **Moderate** |
| Effectiveness of an online mental health strengthening module to build resilience and overcome stress for transitional aged medical students | Moderate | Strong | Strong | Weak | Strong | Strong | **Moderate** |
| Feasibility and acceptability of a culturally adapted psychological first aid training intervention (Preparing Me) to support the mental health and well-being of front-line healthcare workers in China: a feasibility randomized controlled trial | Weak | Strong | Strong | Strong | Strong | Strong | **Moderate** |
| Efficacy of a Text-Based Mental Health Coaching App in Improving the Symptoms of Stress, Anxiety, and Depression: Randomized Controlled Trial | Strong | Strong | Weak | Weak | Strong | Strong | **Moderate** |

# References:

1. Luthar S. Resilience in Development: A Synthesis of Research across Five Decades. Developmental Psychopathology. 2006;3.

2. Sisto A, Vicinanza F, Campanozzi LL, Ricci G, Tartaglini D, Tambone V. Towards a Transversal Definition of Psychological Resilience: A Literature Review. Medicina (Kaunas). 2019;55(11).

3. Hao S, Hong W, Xu H, Zhou L, Xie Z. Relationship between resilience, stress and burnout among civil servants in Beijing, China: Mediating and moderating effect analysis. Personality and Individual Differences. 2015;83:65-71.

4. McCain RS, McKinley N, Dempster M, Campbell WJ, Kirk SJ. A study of the relationship between resilience, burnout and coping strategies in doctors. Postgrad Med J. 2017.

5. Sullivan V, Hughes V, Wilson DR. Nursing Burnout and Its Impact on Health. Nurs Clin North Am. 2022;57(1):153-69.

6. West CP, Dyrbye LN, Shanafelt TD. Physician burnout: contributors, consequences and solutions. J Intern Med. 2018;283(6):516-29.

7. West CP, Dyrbye LN, Sinsky C, Trockel M, Tutty M, Nedelec L, et al. Resilience and Burnout Among Physicians and the General US Working Population. JAMA Netw Open. 2020;3(7):e209385.

8. Lyu C, Ma R, Hager R, Porter D. The relationship between resilience, anxiety, and depression in Chinese collegiate athletes. Frontiers in Psychology. 2022;13.

9. Wang M, Li J, Yan G, Lei T, Rong W, Sun L. The relationship between psychological resilience, neuroticism, attentional bias, and depressive symptoms in college Chinese students. Frontiers in Psychology. 2022;13.

10. García-León MÁ, Pérez-Mármol JM, Gonzalez-Pérez R, García-Ríos MdC, Peralta-Ramírez MI. Relationship between resilience and stress: Perceived stress, stressful life events, HPA axis response during a stressful task and hair cortisol. Physiology & Behavior. 2019;202:87-93.

11. Rahimi B, Baetz M, Bowen R, Balbuena L. Resilience, stress, and coping among Canadian medical students. Can Med Educ J. 2014;5(1):e5-e12.

12. Klainin-Yobas P, Vongsirimas N, Ramirez DQ, Sarmiento J, Fernandez Z. Evaluating the relationships among stress, resilience and psychological well-being among young adults: a structural equation modelling approach. BMC Nursing. 2021;20(1):119.

13. Li Z-S, Hasson F. Resilience, stress, and psychological well-being in nursing students: A systematic review. Nurse Education Today. 2020;90:104440.
